# Stable luminescent iridium(iii) complexes with bis(N-heterocyclic carbene) ligands: photo-stability, excited state properties, visible-light-driven radical cyclization and CO_2_ reduction, and cellular imaging†
†Electronic supplementary information (ESI) available: Additional experimental details, figures and tables. CCDC 1428476–1428479. For ESI and crystallographic data in CIF or other electronic format see DOI: 10.1039/c5sc04458h


**DOI:** 10.1039/c5sc04458h

**Published:** 2016-01-20

**Authors:** Chen Yang, Faisal Mehmood, Tsz Lung Lam, Sharon Lai-Fung Chan, Yuan Wu, Chi-Shun Yeung, Xiangguo Guan, Kai Li, Clive Yik-Sham Chung, Cong-Ying Zhou, Taotao Zou, Chi-Ming Che

**Affiliations:** a State Key Laboratory of Synthetic Chemistry , Institute of Molecular Functional Materials , HKU-CAS Joint Laboratory on New Materials and Department of Chemistry , The University of Hong Kong , Pokfulam Road , Hong Kong , China . Email: cmche@hku.hk; b HKU Shenzhen Institute of Research and Innovation , Shenzhen , China; c The Hong Kong Polytechnic University Shenzhen Research Institute , Shenzhen , PR China; d Department of Applied Biology and Chemical Technology , The Hong Kong Polytechnic University , Hung Hom , Hong Kong , China . Email: sharonlf.chan@polyu.edu.hk

## Abstract

A new class of cyclometalated Ir(iii) complexes supported by various bidentate C-deprotonated (C^N) and *cis*-chelating bis(N-heterocyclic carbene) (bis-NHC) ligands has been synthesized. These complexes display strong emission in deaerated solutions at room temperature with photoluminescence quantum yields up to 89% and emission lifetimes up to 96 μs. A photo-stable complex containing C-deprotonated fluorenyl-substituted C^N shows no significant decomposition even upon irradiation for over 120 h by blue LEDs (12 W). These, together with the strong absorption in the visible region and rich photo-redox properties, allow the bis-NHC Ir(iii) complexes to act as good photo-catalysts for reductive C–C bond formation from C(sp^3^/sp^2^)–Br bonds cleavage using visible-light irradiation (*λ* > 440 nm). A water-soluble complex with a glucose-functionalized bis-NHC ligand catalysed a visible-light-driven radical cyclization for the synthesis of pyrrolidine in aqueous media. Also, the bis-NHC Ir(iii) complex in combination with a cobalt catalyst can catalyse the visible-light-driven CO_2_ reduction with excellent turnover numbers (>2400) and selectivity (CO over H_2_ in gas phase: >95%). Additionally, this series of bis-NHC Ir(iii) complexes are found to localize in and stain endoplasmic reticulum (ER) of various cell lines with high selectivity, and exhibit high cytotoxicity towards cancer cells, revealing their potential uses as bioimaging and/or anti-cancer agents.

## Abstract 2

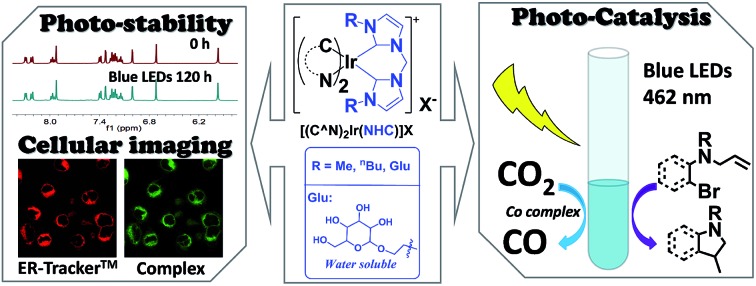
Excited state properties, photo-catalysis and cellular imaging of photo-stable bis-NHC Ir(iii) complexes are described.

## Introduction

Luminescent organometallic complexes of 3rd row transition metals, such as Ir,^1^–^5^ Pt,^6^–^11^ and Au^12^–^19^ are currently receiving burgeoning interest due to their profound applications in materials science,^20^–^22^ biology,^23^,^24^ and organic synthesis.^25^–^27^ In particular, the favourable emission properties of cyclometalated Ir(iii) complexes have been harnessed for applications by many research groups with examples such as the development of high performance OLEDs by Thompson and co-workers^3^–^5^,^28^–^31^ and the design of bioimaging and cellular probes by Lo and co-workers.^32^–^36^ Over the past decade, as a result of extensive work most notably by MacMillan,^37^,^38^ Yoon,^27^,^39^–^41^ and Stephenson and co-workers,^42^–^45^ luminescent cyclometalated iridium(iii) complexes are widely used in photo-redox catalysis, which has been shown to have useful applications in organic synthesis.

In 2008, Yoon and co-workers^40^ reported [2 + 2] enone cycloadditions, and MacMillan and co-workers^38^ published the alkylation of aldehydes, both of which were catalysed by triplet metal-to-ligand-charge-transfer (^3^MLCT) excited state of [Ru(bpy)_3_]^2+^ generated upon visible-light irradiation. Subsequently, Stephenson and co-workers achieved the reductive dehalogenation of activated alkyl halides catalysed by [Ru(bpy)_3_]^2+^,^44^ and reductive dehalogenation of alkyl, alkenyl and aryl iodides by using *fac*-Ir(ppy)_3_ as photo-redox catalyst.^45^ Compared to [Ru(bpy)_3_]^2+^ and *fac*-Ir(ppy)_3_, the application of luminescent platinum(ii) photo-catalysts is still nascent. Recently, our group and Wu's group demonstrated that pincer Pt(ii) complexes are capable of catalysing light induced C–C bond formation.^46^,^47^

The important features that allow luminescent transition metal complexes to act as useful photo-redox catalysts or photo-sensitizers for light induced reactions include: (1) the long lifetime of their electronic excited states, thus allowing bimolecular reaction to proceed in solution; (2) their electronic excited states as both strong reducing and oxidizing reagents with reduction potentials systematically varied by the auxiliary ligands.^41^ For the photo-catalysis to have practical interest, the design of highly stable photo-redox catalysts with long-lived electronic excited states in solution is desirable.

Our endeavour to develop transition metal photo-catalysis is to use visible light for activation of small molecules such as CO_2_, described in this work, and for C–X bond functionalization. A major consideration is to utilize visible light, which falls within the solar spectrum and avoids deleterious high-energy UV-initialized photochemical side reactions.^48^,^49^ In the literature, platinum group metal complexes and semi-conductors are usually used as photo-redox catalysts for the photochemical reduction of CO_2_.^50^ Earlier examples of transition metal photo-catalysts used for the photochemical reduction of CO_2_ include cobalt porphyrins,^51^ Re(bpy)(CO)_3_Cl^52^,^53^ and Ir(terpy)(ppy)Cl.^54^ More recently, systems comprising *fac*-Ir(ppy)_3_ in conjunction with [Ni(^Pr^bimiq1)]^2+^,^55^ Fe(porphyrin),^56^ [Co(TPA)Cl]Cl,^57^ (TPA = tris(2-pyridylmethyl)amine) and [Co(N5)]^2+^ (N5 = 2,13-dimethyl-3,6,9,12,18-pentaazabicyclo-[12.3.1]octadeca-1(18),2,12,14,16-pentaene)^58^ were reported for photochemical reduction of CO_2_.

The stability of photo-catalysts is an important issue for the practical application of transition metal photochemistry. Numerous studies revealed that the photochemically active excited states of [Ru(bpy)_3_]^2+^,^59^–^61^ [Ir(ppy)_2_(bpy)]^+^,^60^,^62^ and *fac*-Ir(ppy)_3_^55^,^57^,^63^ are not stable under light irradiation for a long period of time as a result of dissociation of coordinated ligand(s) presumably *via* low lying d–d excited state(s). To address the photo-stability issue, we considered the use of N-heterocyclic carbene (NHC) ligands which have been receiving burgeoning attention in coordination chemistry due to their strong σ-donor strength to develop robust metal photo-sensitizers and photo-catalysts.^64^–^70^ Also, the *N*-substituent of NHC ligands can be used to tune both the physical and chemical properties of the resultant photo-active transition metal complexes such as their solubility in various solvents including water. NHC ligands functionalized with carboxylate, sulfonate, amine/ammonium, and alcohol motifs have been reported for the development of water-soluble transition metal catalysts for Suzuki coupling, hydrosilylation, hydrogenation, olefin metathesis and CO_2_ reduction.^65^,^71^

**Chart 1:**
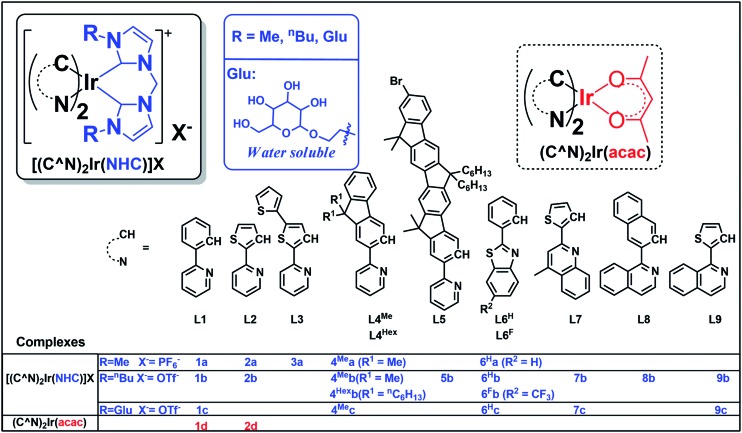
Iridium(iii) complexes in this work. Complexes **1a**,^73^,^74^ **1b**,^73^**1d**^28^ and **2d**^31^ have been reported in the literature.

Compared with Ir(iii) complexes supported by bidentate acetylacetonate^28^ and/or 2,2′-bypyridine^1^ ligands, the photophysical and application studies of the related bis-NHC Ir(iii) complexes are relatively scarce. In 2010, cationic bis-NHC Ir(iii) complexes were reported by De Cola and co-workers^72^ to have application in blue-light emitting electrochemical cells; subsequently, a number of bis-NHC Ir(iii) complexes were reported for photophysical and biological studies.^65^,^67^,^72^–^74^ In this work, a series of strongly luminescent Ir(iii) complexes (Chart 1) containing bis-NHC ligands and visible light absorbing C-deprotonated (C^N) ligands was synthesized and their photophysical and electrochemical properties were examined. These complexes display high photo-stability and are strongly emissive with long lifetimes of up to 96 μs in solution at room temperature. The water-soluble luminescent Ir(iii) complexes, containing the glucose-functionalized NHC ligand, were found to be active photo-catalysts for radical cyclization leading to the formation of 5-membered pyrrole rings in aqueous media with high substrate conversions and yields. One of the photo-stable Ir(iii) complexes was utilized as a photo-sensitizer and in conjunction with a recently reported catalyst [Co(TPA)Cl]Cl to convert CO_2_ into CO with a turnover number (TON) > 2400, selectivity in gas phase > 95% and yield of 5.6% (1 mL out of 18 mL of CO_2_ was converted into CO at 5 μM concentration of Co complex). Some of the complexes were also demonstrated as potential bioimaging and/or anti-cancer agents.

## Results

### Synthesis, characterization and photo-stability of [(C^N)_2_Ir(NHC)]X complexes

The structures of 18 bis-NHC Ir(iii) complexes synthesized in this work (**1c** and **2a**, **2b**, **3–9**), together with previously reported Ir(iii) complexes **1a**,^73^,^74^ **1b**,^73^**1d**^28^ and **2d**,^31^ are depicted in Chart 1. Complexes **1–9** were synthesized in good yields by refluxing [(C^N)_2_Ir(μ-Cl)]_2_ with bis(imidazolium) salts in the presence of silver(i) oxide in 2-methoxylethanol. Details of synthesis and characterization data of ligands and complexes are provided in the ESI.† Notably, ^1^H NMR spectra of **7b** and **7c** show poorly resolved peaks in the aromatic region (6.7–8.2 ppm) at ambient temperature, and hence ^1^H NMR spectra are recorded from 238 K to 300 K (Fig. S1, ESI†) in order to assure the purity of **7b** and **7c**.

Complexes **1a–4^Me^a**, **1b–4^Me^b**, **4^Hex^b–9b** with *N*-methyl or *N*-butyl substituent on bis-NHC ligands are soluble in most common aprotic solvents, but not in protic solvents *e.g.* methanol (MeOH) or water. Complexes **1c**, **4^Me^c**, **6^H^c**, **7c** and **9c** with glucose functionalized bis-NHC ligand are soluble in MeOH, ethanol (EtOH) and water.

**Fig. 1:**
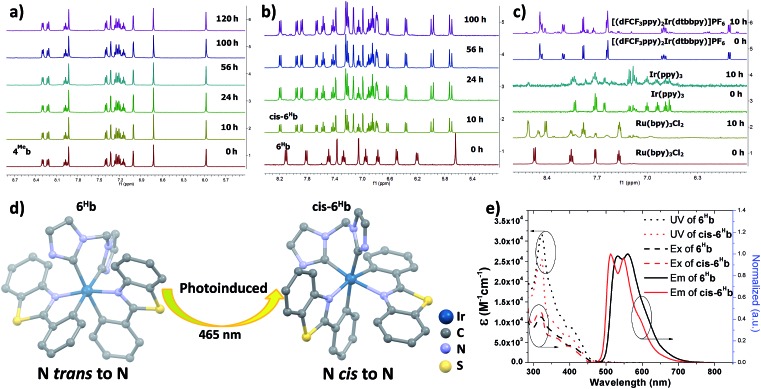
^1^H NMR spectra for (a) **4^Me^b**; (b) **6^H^b**; (c) Ru(bpy)_3_Cl_2_, *fac*-Ir(ppy)_3_ and [(dFCF_3_ppy)_2_Ir(dtbbpy)]PF_6_ in deuterated MeCN solution for irradiating by blue light (12 W, *λ*_max_ = 462 nm)^46^ (all solutions are degased by nitrogen gas for 10 min); (d) crystal structure diagrams showing the photoinduced transformation of coordination for Ir metals in **6^H^b** and ***cis*-6^H^b** (butyl group on bis-NHC ligand and hydrogen atoms are omitted for clarity); (e) UV/Vis absorption (dotted line), excitation (dashed line) and emission (solid line) spectra of solutions of **6^H^b** and ***cis*-6^H^b** (concentration of 2.0 × 10^–5^ M) in degassed DCM at 298 K.

The photo-stability of these Ir(iii) complexes with bis-NHC ligands was examined by using **4^Me^b** and **6^H^b** as representative examples. **4^Me^b** and **6^H^b** in degassed deuterated MeCN were irradiated using blue light (12 W blue LEDs) for 5 days. The photolysis was monitored by ^1^H NMR spectroscopy. As depicted in Fig. 1a, in the case of **4^Me^b**, less than 5% of the complex was observed to undergo photochemical decomposition after irradiation of 120 h, revealing its outstanding photo-stability. Under the same conditions, Ru(bpy)_3_Cl_2_, *fac*-Ir(ppy)_3_ and [(dFCF_3_ppy)_2_Ir(dtbbpy)]PF_6_ (dFCF_3_ppy = 3,5-difluoro-2-[5-(trifluoromethyl)-2-pyridinyl]phenyl; dtbbpy = 4,4′-bis(1,1-dimethylethyl)-2,2′-bipyridine) were found to decompose after 10 h irradiation as revealed by the changes of their ^1^H NMR spectra (Fig. 1c).

Interestingly, irradiation of **6^H^b** using blue LEDs for 10 h led to a clean and quantitative conversion to a new species which did not show further changes upon subsequent irradiation for another 90 h (Fig. 1b, this species is noted as ***cis*-6^H^b**). The yield of scale synthesis of ***cis*-6^H^b** from the irradiation of degassed MeCN solution of **6^H^b** (100 mg in 4 mL MeCN) was 94%. ***cis*-6^H^b** was found to be stable upon standing in solution in the dark for another 40 h, or exposed to air for another 20 h (Fig. S2, ESI†). To verify that the transformation of **6^H^b** to ***cis*-6^H^b** was caused by visible-light irradiation, a negative control experiment was conducted by keeping **6^H^b** in deuterated MeCN in the dark (Fig. S2, ESI†), and no structural changes were detected by ^1^H NMR spectroscopy.

The UV-Vis absorption, emission and excitation spectra of ***cis*-6^H^b** measured in DCM solution at 298 K are different from those of **6^H^b** with hypsochromic shifts in peak maxima (Fig. 1e). The emission band for ***cis*-6^H^b** displays vibronic spacings of 1245 cm^–1^ while that of **6^H^b** shows spacings of 908 cm^–1^. Taking into consideration the ESI-MS and spectroscopic data of ***cis*-6^H^b**, ***cis*-6^H^b** is likely a structural isomer for **6^H^b**. The exact structure of ***cis*-6^H^b** has been determined by X-ray crystallography.

### X-Ray crystallography

Crystals of **4^Me^b** (with PF_6_^–^ counter anion), **6^H^b**, ***cis*-6^H^b** and **7b** suitable for X-ray crystallographic analysis were obtained by slow diffusion of diethyl ether into DCM (**6^H^b**), MeCN (**4^Me^b** and **7b**) and chloroform (***cis*-6^H^b**) solution of these complexes, respectively. Perspective views of **6^H^b** and ***cis*-6^H^b** are shown in Fig. 1d (those of **4^Me^b**, **7b** are shown Fig. S3 in ESI†). Selected bond lengths and angles are compiled in Table S1 (ESI†). Similar to the reported examples,^72^**6^H^b** adopts a distorted octahedral geometry (Fig. 1), the iridium atom is coordinated by two cyclometalated C^N ligands and one bis-NHC ligand. The two C^N ligands adopt a mutually eclipsed configuration, with the two N atoms (N1 and N2) *trans* to each other and with Ir–N bond length of 2.067(2) and 2.071(2) Å. The two substituted phenyl rings are oriented *cis* to each other with Ir–C bond length of 2.054(2) and 2.077(2) Å. The C^NHC^ atoms of bis-NHC ligand are *trans* to two C atoms from C^N ligands. The Ir–C^NHC^ distances of 2.105(2) Å and 2.128(2) Å are comparable to the literature values of Ir–C^NHC^ bonds *trans* to phenyl groups.^72^ The C^NHC^–Ir–C^NHC^ bite angles of **4^Me^b** (84.7(3)^o^), **6^H^b** (85.10(9)^o^), **7b** (85.58(6)^o^) and that of reported [(dfppy)_2_Ir(NHC^Me^)]PF_6_ (85.28(15)^o^) (dfppy = 4,6-difluoro-phenylpyridine) are consistent.^72^ The structures of **4^Me^b** and **7b** are in line with that of **6^H^b**. Interestingly, for ***cis*-6^H^b** the N atoms from the two C^N ligand are *cis* to each other (Fig. 1d), indicating that **6^H^b** and ***cis*-6^H^b** are structural isomers. The crystallographic refinement parameters for **4^Me^b**, **6^H^b**, ***cis*-6^H^b** and **7b** are summarized in Table S2 (ESI†).

### Electronic absorption and emission spectroscopy

**Fig. 2:**
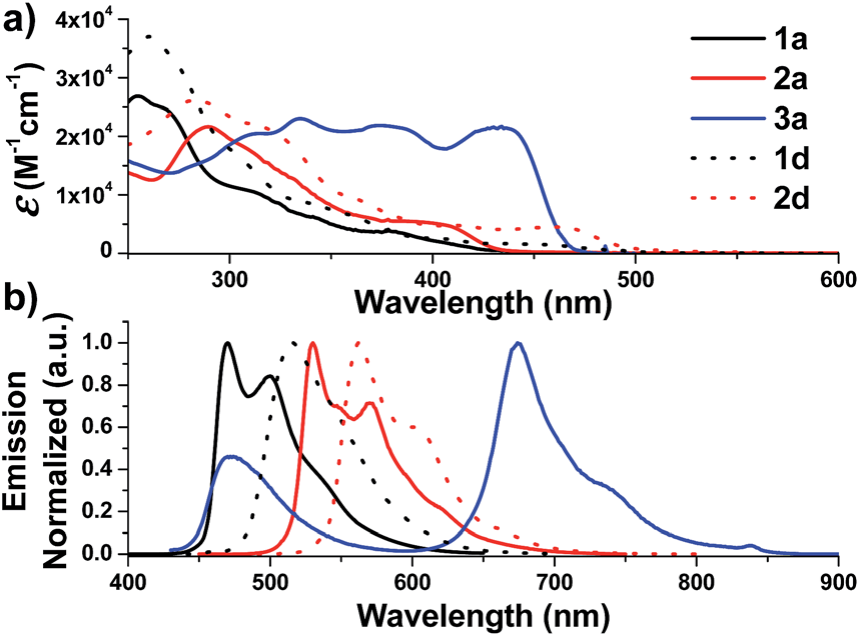
UV-Vis absorption (top) and emission (bottom) spectra of solutions of **1a–3a** and **1d–2d** in degassed DCM (concentration of 2.0 × 10^–5^ M) at 298 K.

**Fig. 3:**
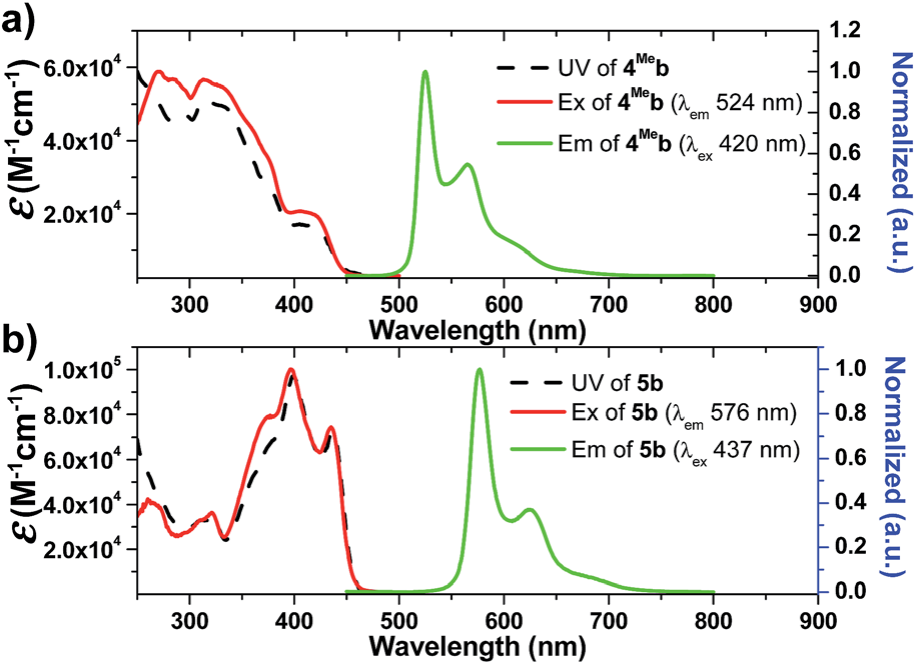
UV/Vis absorption (black dashed line), excitation (red solid line) and emission (green solid line) spectra of solutions of (a) **4^Me^b** (5.0 × 10^–6^ M); (b) **5b** (1.0 × 10^–6^ M) in degassed DCM at 298 K.

Photophysical data of **1–9** including their UV/Vis absorption and emission maxima, emission lifetimes and photo-luminescent quantum yields are tabulated in Table 1. All the complexes show intense high-energy absorptions ranging from 250 to 380 nm and lower-energy absorptions at 416–494 nm (Fig. 2, 3 and S4 in ESI†). The high-energy absorptions are attributed to the ^1^π → π* transition of the ligands, whereas the low energy absorption bands should be ascribed to the admixture of metal-to-ligand charge transfer (MLCT) transitions and ligand centered (LC) π–π* transitions.^72^ The charge transfer (CT) assignment is supported by the energy trend observed in the complexes with different C^N ligands. For example, the low-energy absorptions of **8b** and **5b** reveal significant red-shifts from those of **1b** and **4^Me^b** respectively. This can be rationalized by the extended π-conjugation of the C^N ligand in **8b** and **5b**, leading to the lowering of the π*(C^N) orbitals and hence decreases in CT energy. These red-shifts in the UV-Vis absorptions, together with the large molar absorptivities in the visible region (*e.g.*, **5b**: 7.2 × 10^4^ M^–1^ cm^–1^), allow the complexes to show strong absorption in the visible region. This is believed to be crucial for harvesting visible light in the solar spectrum as well as avoiding UV-initiated side reactions due to the use of UV in the photo-catalysis by the Ir(iii) complexes. As revealed by the UV absorption spectra of **6^F^b** and **6^H^b** (Fig. S4b, ESI†), a bathochromic shift about 500 cm^–1^ is observed for the lower-energy absorption band(s). These could be attributed to the electron-withdrawing group of CF_3_ on the cyclometalated ligand which lowers the C^N based LUMO level.

**Table 1:** Photophysical data of complexes **1–9**

Complex	Medium^*a*^	Absorption (*λ*_max_/nm) (10^–3^*ε*/M^–1^ cm^–1^)	Emission *λ*_em_/nm (*τ*/μs)	*Φ* ^*d*^/%
**1a**	CH_2_Cl_2_	255 (26.8), 267 (24.9), 311 (10.6), 342 (6.1), 380 (3.7), 416 (1.2)	470 (2.1), 499, 534	89
**1b**	CH_2_Cl_2_	254 (36.8), 266 (35.5), 311 (15.5), 342 (9.1), 381 (5.82), 416 (2.16)	470 (2.1), 500, 534	89
**1c**	H_2_O	254 (36.8), 266 (35.5), 311 (15.5), 342 (9.1), 381 (5.82), 416 (2.16)	469 (2.0), 498, 534	89
**2a**	CH_2_Cl_2_	252 (13.5), 290 (21.7), 332 (12.6), 369 (6.07), 405 (4.92)	530, 547, 570 (3.03)	11.1
**3a**	CH_2_Cl_2_	252 (15.5), 282 (15.1), 313 (20.5), 335 (23.1), 375 (21.9), 389 (20.9), 440 (20.9)	472, 674 (4.8), 824	0.2
**4^Me^a**	CH_2_Cl_2_^*b*^	260 (31.4), 297 (30.0), 316 (33.5), 334 (32.3), 360 (22.4), 378 (16.8), 421 (10.5)	524, 564 (28.6), 614	65
**4^Me^b**	CH_2_Cl_2_^*b*^	267 (52.2), 297 (46.8), 316 (50.9), 335 (49.0), 358 (36.3), 376 (27.3), 421 (16.2)	525, 566 (28.7), 614	75
**4^Hex^b**	CH_2_Cl_2_^*b*^	268 (42.1), 299 (42.3), 320 (50.4), 338 (49.2), 364 (32.6), 379 (25.6), 421 (14.6)	527, 568 (32.7), 616	82
**5b**	CH_2_Cl_2_^*c*^	260 (54.1), 273 (42.5), 310 (32.9), 323 (34.8), 377 (67.2), 398 (97.0), 436 (71.6)	576 (96.1), 625, 683	22.6
**6^H^b**	CH_2_Cl_2_	271 (23.0), 321 (25.0), 377 (9.57), 407 (6.81), 436 (3.38)	531, 560 (6.4)	78
**6^H^c**	H_2_O	268 (21.4), 321 (22.1), 377 (8.06), 407 (5.49), 436 (2.42)	536, 560 (5.0)	66
**6^F^b**	CH_2_Cl_2_	270 (23.0), 323 (32.1), 383 (10.7), 415 (7.88), 447 (4.08)	530, 558 (3.2)	68
**7b**	CH_2_Cl_2_	296 (26.8), 333 (18.5), 388 (9.31), 437 (7.76), 467 (4.60)	582, 623 (6.2)	36
**7c**	H_2_O	293 (25.9), 330 (17.4), 386 (9.63), 432 (6.92), 464 (3.71)	583, 622 (5.9)	32
**8b**	CH_2_Cl_2_	260 (80.1), 290 (48.5), 315 (35.4), 376 (34.5), 429 (7.52), 485 (3.47), 494 (2.88)	618, 659 (5.2)	3
**9b**	CH_2_Cl_2_	313 (24.2), 334 (23.8), 384 (12.7), 428 (10.7), 457 (8.20), 485 (3.47)	617, 667 (7.4)	13
**9c**	H_2_O	315 (18.4), 380 (9.69), 420 (7.6), 454 (4.95), 475 (2.15)	620 (4.5), 668	9

^*a*^Measured in degassed CH_2_Cl_2_ and water (2.0 × 10^–5^ M) at 298 K.

^*b*^ **4^Me^a**, **4^Me^b** and **4^Hex^b** were measured at the concentration of 5 × 10^–6^ M.

^*c*^ **5b** was measured at the concentration of 1 × 10^–6^ M.

^*d*^Phosphorescence quantum yields were measured by using [Ru(bpy)_3_](PF_6_)_2_ (*Φ* = 0.062 in MeCN) as standard.

On the other hand, the *N*-alkyl substituents on the bis-NHC ligand are found to have only minor effects, if any, on the UV/Vis absorption spectra of the Ir(iii) complexes, as revealed by the overlaid spectra of the group of **1a**, **1b**, **1c** (Fig. S4a, ESI†). Complex **1a** absorbs weakly at the wavelength from 430 nm to 500 nm with molar absorptivity less than 500 M^–1^ cm^–1^ in contrast to the high values of 2500 M^–1^ cm^–1^ for **1d** with the same C^N luminophore. The calculated wavelength for ground state HOMO → LUMO (S_0_ → S_1_ transition) is 369 nm and 414 nm for **1a** and **1d**, respectively (Fig. S4e and f, ESI†). These calculated values are in reasonable agreement with the corresponding experimental absorption *λ*_max_ values 410 and 460 nm respectively.

Complexes **1–9** display strong phosphorescence in deaerated solution at room temperature (Fig. 2, 3 and S4 (ESI†); Table 1). All the complexes show vibronic-structured emissions, and their emission lifetimes are found to be in the microsecond regime. For example, **4^Me^b** exhibits structured emission bands with vibrational spacings of ∼1380 cm^–1^ and a long emission lifetime of 28.2 μs. Only small negative solvatochromic effects on the emission at 524 nm are found (±5 nm; Fig. S4d, ESI†). These findings, together with TD-DFT calculations, suggest that the photoluminescence of the complexes is derived from triplet metal-perturbed ligand-centred (^3^LC) π–π* excited states. On the other hand, the structureless emission of **1d** (the acetylacetonate (acac) analogous of **1a**) at 516 nm should be ascribed to the ^3^MLCT/LLCT emission.^2^ Interestingly, complex **3a** (*Φ* = 0.2%; *τ* = 4.8 μs) displays dual emission (Fig. 2) in contrast to the single emission of the acac analogue **3d**,^75^ suggesting the possibility of modulation of photophysical properties of Ir(iii) complexes by the NHC ligands.

Changes in chemical structure of the cyclometalated ligands also result in a profound effect on the photophysical properties of the Ir(iii) complexes. For example, **5b** displays a significant red-shift in emission maximum (576 nm) when compared to **4^Me^b** and **4^Hex^b** (525 and 527 nm respectively). This can be rationalized by the extended π-conjugation of the C^N ligands, leading to the decrease in energy of metal-perturbed ^3^LC emission. Moreover, for **4^Me^a**, **4^Me^b** and **4^Hex^b**, their photoluminescence quantum yields in solution are affected by the substituents on the fluorenyl moiety as well as the *N*-alkyl groups of the bis-NHC ligands, *e.g.* complex **4^Hex^b** exhibits a higher photoluminescence quantum yield than **4^Me^a** and **4^Me^b**. This is probably attributed to the fact that the long hexyl and *N*-butyl chains disfavour intermolecular stacking interactions among the planar C^N ligands, leading to reduced triplet–triplet annihilation and a higher photoluminescence quantum yield.

In addition, **5b** shows a significantly longer emission lifetime (96.1 μs) than **4^Me^b** (28.7 μs). This might be due to the reduced metal character in the electronic excited state of **5b**, and hence slower triplet radiative decay. Similarly, with a smaller parentage of metal character in the frontier molecular orbitals, **6^H^b** (6.4 μs) shows a longer emission lifetime than **6^F^b** (3.2 μs). These long-lived triplet excited states allow the Ir(iii) NHC complexes to undergo a variety of photochemical reactions, notably for visible-light-driven photo-catalytic reductive C–Br bond cleavage and CO_2_ reduction which will be illustrated later.

### Electrochemistry

The electrochemical data of **1b–9b** are summarized in Table 2 (all values *versus* Ag/AgNO_3_, scan rate of 100 mV s^–1^, 0.1 M ^*n*^Bu_4_NPF_6_ in MeCN as supporting electrolyte) and Table S3† (values *vs.* Cp_2_Fe^+/0^, ESI†). These complexes display one irreversible oxidative wave at *E*_pa_ = 0.62–1.04 V and one irreversible reductive wave at *E*_pc_ = –2.52 to –1.94 V (*vs.* Ag/AgNO_3_). The cyclic voltammograms of **1a** have been reported elsewhere^74^ with the oxidation potential of +1.16 V (*vs.* Ag/AgNO_3_) in CH_2_Cl_2_. By comparing with the electrochemical data of **1d** and **2d**^2^, both of which have the ancillary acac ligand, the first oxidation wave of **1a** should originate from Ir(iii)-centred/C^N ligand-centred oxidation. This assignment is further supported by the observation of the more positive oxidation potential of **6^F^b** (+1.04 V) than **6^H^b** (+0.98 V). The electron-withdrawing –CF_3_ group of **6^F^b** will lower the energy levels of the π(C^N) orbitals, leading to the more positive oxidation potential of **6^F^b**. On the other hand, **4^Me^b** and **5b** have more extended π-conjugated C^N ligands than **1b**. This will result in an increase in the energy level of π(C^N) orbitals, thus **4^Me^b** and **5b** can undergo oxidation more readily than **1b** and hence less positive oxidation potentials are found.

**Table 2:** Electrochemical data^*a*^ of bis-NHC Ir(iii) complexes

Complex	*E*_pa_ ^*b*^/V	*E*_pc_ ^*c*^/V
**1b**	0.87	–2.52
**2a**	0.64	–2.24
**4^Me^a**	0.74	–2.41
**4^Me^b**	0.79	–2.40
**4^Hex^b**	0.74	–2.44
**5b**	0.72	–2.25
**6^H^b**	0.98	–2.14
**6^F^b**	1.04	–1.94
**7b**	0.69	–2.09
**8b**	0.62	–1.94
**9b**	0.62	–2.16

^*a*^Supporting electrolyte: 0.1 M ^*n*^Bu_4_NPF_6_ in MeCN and values are recorded *vs.* Ag/AgNO_3_ (0.1 M) in MeCN; Cp_2_Fe^+/0^ occurs at the range of 0.05–0.08 (V) *vs.* Ag/AgNO_3_.

^*b*^Values refer to oxidation peak potential (*E*_pa_) at 25 °C for irreversible couples at a scan rate of 100 mV s^–1^.

^*c*^Values refer to reduction peak potential (*E*_pc_) for the irreversible reduction waves.

**Fig. 4:**
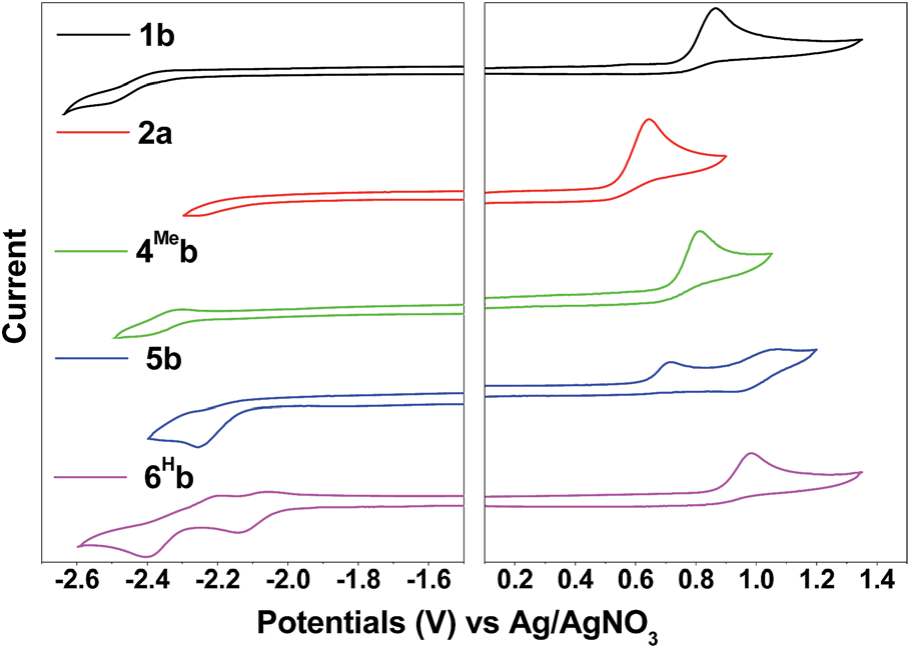
Cyclic voltammograms of **1b**, **2a**, **4^Me^b**, **5b** and **6^H^b** in MeCN with ^*n*^Bu_4_NPF_6_ (0.1 M) as supporting electrolyte. Conditions: glass-carbon, working electrode, scan rate: 100 mV s^–1^.

Compared with **1b**, [(dfppy)_2_Ir(bis-NHC^Bu^)]PF_6_ (ref. 72) displays a more anodic oxidation potential of *E*_ox_ = 1.04 V and a similar reduction potential of *E*_re_ = –2.37 V (*vs.* Cp_2_Fe^+/0^), which is attributed to stabilization of HOMOs as a result of the presence of highly electron-withdrawing F substitution on the C^N ligand (dfppy). For the irreversible reduction wave, a less negative reduction potential is found for **6^H^b** (*E*_pc_ = –2.14 V *vs.* Ag/AgNO_3_, Table 2, Fig. S4†), as compared to that of **1b** (*E*_pc_ = –2.52 V *vs.* Ag/AgNO_3_, Table 2, Fig. 4) which has a less extended π-conjugation of the C^N ligand. The reductive wave is further anodically shifted in the case of **6^F^b** (*E*_pc_ = –1.94 V *vs.* Ag/AgNO_3_, Table 2, Fig. S5a, ESI†). Therefore, the reduction process should be localized on the C^N ligands.

### Excited state properties

**Fig. 5:**
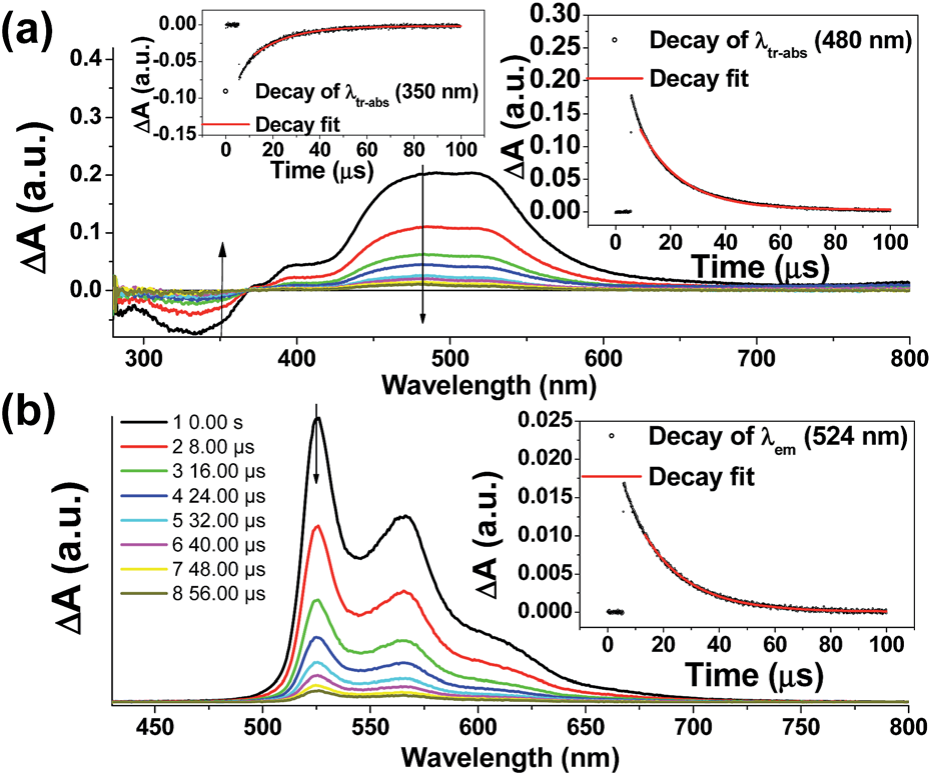
Time-resolved spectra of **4^Me^b** (a) tr-abs (insets: decay of tr-abs at *λ* = 350 nm and 480 nm); (b) tr-em (decay of tr-em at *λ* = 524 nm) spectra recorded at specified times after laser pulse excitation (355 nm) in degassed MeCN at 298 K.

**Fig. 6:**
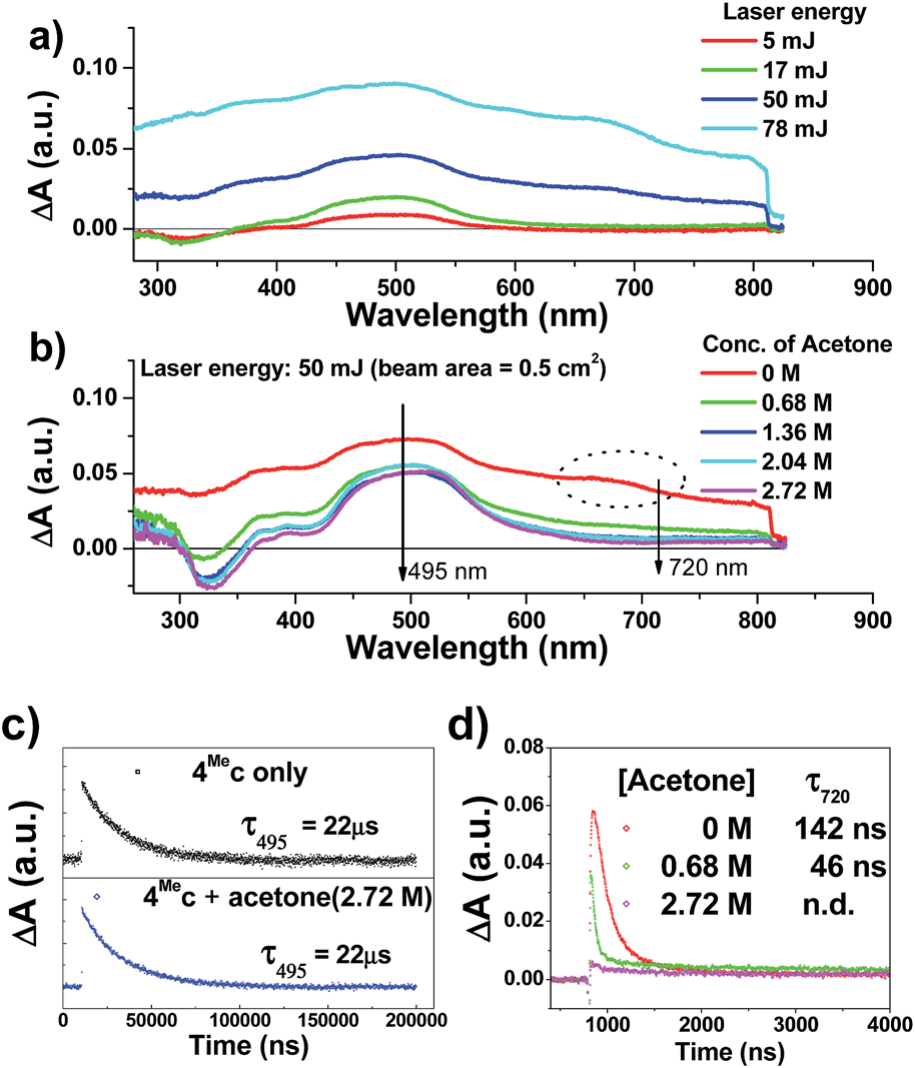
Transient absorption spectra recorded (a) under specified laser energy; (b) in presence of specified concentrations of acetone; of a degassed aqueous solution of **4^Me^c** (about 1 × 10^–5^ M). Kinetic studies of (c) λ_tr-abs_ (495 nm) and (d) λ_tr-abs_ (720 nm) in the absence/presence of specified concentrations of acetone.

Nano-second transient absorption and emission spectroscopic (tr-abs and tr-em) measurements were undertaken for the highly emissive bis-NHC Ir(iii) complexes with long-lived electronic excited states *i.e.***4^Me^b** (Fig. 5) and **4^Me^c** (Fig. 6a) in MeCN and water respectively. These two complexes have the same lumophore but different functionalized NHC groups, leading to a different solubility in solvents, such as one is soluble in organic solvents and the other in water. The tr-abs and tr-em spectra of the other bis-NHC Ir(iii) complexes *e.g.***5b** in MeCN (Fig. S6a, ESI†), and **6^H^c** (Fig. S6b, ESI†) in water were also recorded and the results can be found in the ESI.†

The time-resolved absorption and emission spectra of **4^Me^b** recorded at various time intervals after excitation at 355 nm are depicted in Fig. 5. The kinetic decay analysis of bleaching of ground-state of **4^Me^b** (*τ*_350 nm_ = 15.4 μs, Fig. 5a, left inset) matches well with the growth of the absorption of triplet excited state (*τ*_480 nm_ = 15.2 μs, Fig. 5a, right inset) as well as the emission lifetime (*τ*_524 nm_ = 16.5 μs, Fig. 5b, right inset) in MeCN at 298 K.

The transient absorption spectra of **4^Me^c** in aqueous solution recorded at different energies of laser beams (355 nm) reveal different spectral changes. As depicted in Fig. 6a, in addition to the growth of the absorption of triplet excited state of **4^Me^c** from 380 to 700 nm, the emergence of an absorption band from 650 to 730 nm was observed at laser pulse energy ≥ 50 mJ (beam area: 0.5 cm^2^). This absorption band could be quenched upon addition of acetone (Fig. 6b and S7a and b, ESI†), but no quenching of transient absorption at 495 nm and emission at 524 nm were observed in the presence of acetone (Fig. 6c and S7, ESI†). The decay rate constant monitored at 720 nm of **4^Me^c** (Fig. 6d, absence of acetone) was much faster than that measured at 495 nm in Fig. 6c/S7c† and decay of emission at 524 nm (Fig. S7d, ESI†). In view of these different kinetic behaviours, the transient absorption from 650 to 730 nm depicted in Fig. 6b might originate from hydrated electrons e_aq_^–^,^76^,^77^ which were formed by the photo-induced ionization of **4^Me^c** in aqueous solution upon excitation with high energy laser beams. This is in line with a reported photoionization of [Pt_2_(POP)_4_]^4–^,^78^ as well as the findings in the studies of solvated electron with acetone.^79^,^80^ Similarly, complex **6^H^c** in aqueous solution was also observed to undergo photo-ionization as revealed by the increase in transient absorption in the region of 600 to 700 nm (Fig. S8b, ESI†). For **4^Me^b**, its transient absorption spectra monitored at high laser pulse energy in MeCN exhibit similar profiles as for lower energy (Fig. S8b and c, ESI†). However, newly generated long-lived species have been observed at high laser pulse energies after 100 μs (Fig. S8b–e, ESI†), revealing that photo-ionization of **4^Me^b** with the generation of [**4^Me^b**]^+^ likely occurs. The accompanying solvated electrons are not observed in this case due to ready quenching by MeCN.^81^

Based on the electrochemical data and the determination of *E*_0–0_ from the spectroscopic measurements, the excited state redox properties of the bis-NHC Ir(iii) complexes can be estimated (Table S3, ESI†). The triplet excited states of the complexes are found to be powerful oxidants and reductants, and some of them are even more reactive towards photoredox reactions than [Ru(bpy)_3_]^2+^ and *fac*-Ir(ppy)_3_. A representative example is **4^Me^b** (*E*(Ir^IV/III*^) = –1.51 V *vs.* SCE) (here Ir^IV^ is a simple notation to denote the oxidized Ir^III^ species, the site of the oxidation can be the metal and/or C^N ligand), which is a stronger reductant than [Ru(bpy)_3_]^2+^(*E*(Ru^III/II*^) of –0.81 *vs.* SCE).^37^,^82^ As a result, it is anticipated that the bis-NHC Ir(iii) complexes described herein, upon photoexcitation in the visible-light region, can catalyse a number of reactions which are not feasible by the widely used [Ru(bpy)_3_]^2+^.

### Photo-catalysis

**Chart 2:**
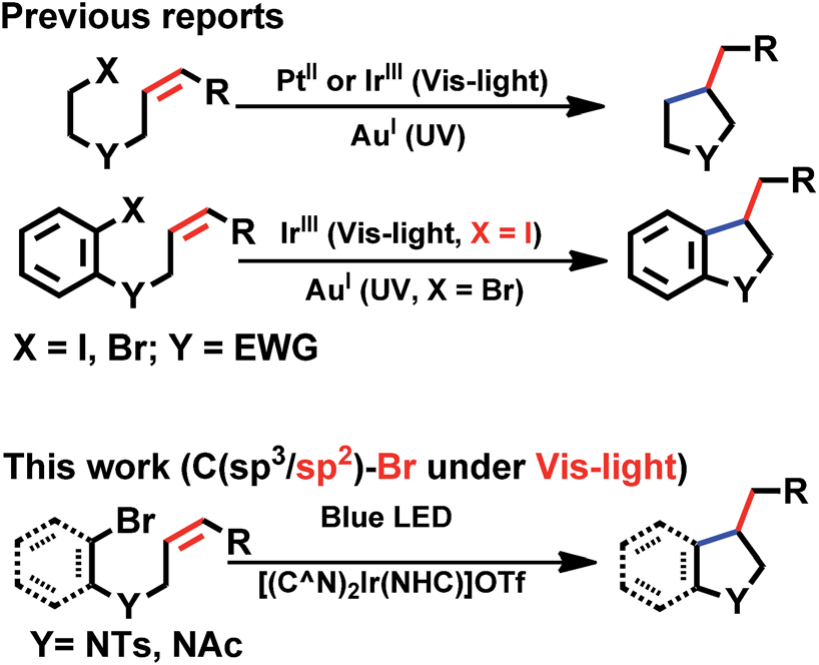
General photo-catalytic reactions by cyclometalated complexes. EWG = electron-withdrawing group.

With good photo-stability, long excited state lifetime, favourable absorption in the spectral region of blue LED and tunable photo-redox properties, these bis-NHC Ir(iii) complexes have been investigated for photo-redox reactions (Chart 2), examples of which are described in the following section.

We considered a recent work by Lee and co-workers^83^ on visible-light-induced reductive cyclization of aromatic iodides and bromides to form indoline using [(ppy)_2_Ir(dtbbpy)]PF_6_ as photo-catalyst (PC). Yet, aryl bromides are found to be less reactive than iodides.^84^ Barriault and co-workers addressed this issue by using dinuclear gold(i) complexes ([Au_2_(dppm)_2_]OTf_2_) as photo-catalysts.^85^ However, this gold complex only shows absorption in the high-energy UV region (*λ* < 300 nm), which may result in destructive effects on the products and/or lead to undesired side reactions.

As the present bis-NHC Ir(iii) complexes show strong absorption at *λ* > 400 nm, they were used to photo-catalyse the reductive cyclization of aryl bromides using blue LEDs. Among the bis-NHC Ir(iii) complexes examined, **1b**, **4^Me^b** and **6^H^b** displayed good photo-catalytic activity for reductive cyclization of aryl bromide (**A1**) in terms of both substrate conversion and product yield (Table 3).

**Table 3:** Screening bis-NHC Ir(iii) complexes for photo-catalysis^*a*^

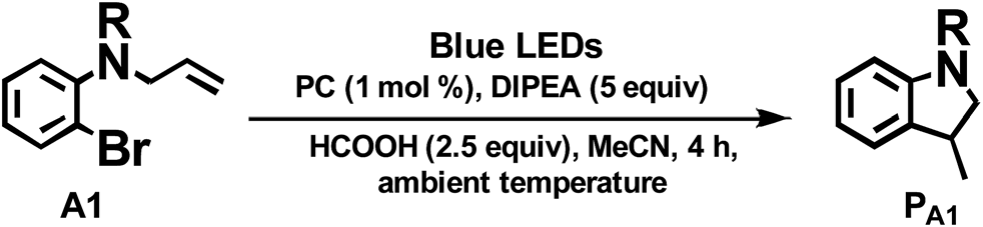			
Entry^*b*^	PC	Conversion^*c*^/%	Yield^*c*^/%
1	**1b**	93	59
2	**2b**	1	0
3	**4^Me^b**	90	59
4	**5b**	90	52
5	**6^H^b**	93	59
6	**6^F^b**	80	44
7	**7b**	49	24
8	**8b**	11	7.3
9	**9b**	0.1	0

^*a*^Complex **3a** was not tested because of the low quantum yield (0.2%, see Table 1).

^*b*^Procedure: substrate 50 μmol, **PC** (1 mol%), DIPEA (5 equiv.), HCOOH (2.5 equiv.) in 4 mL MeCN solution was degassed by nitrogen, and irradiated by blue light (12 W, *λ*_max_ = 462 nm) at ambient temperature for 4 h.

^*c*^Determined by ^1^H NMR spectroscopy by adding internal standard of 5,5′-dimethyl-2,2′-bipyridine.

In the case of another aryl bromide substrate **A2**, **4^Me^b** showed both good substrate conversion and product yield (Table 4, entries 4–6). It was chosen as photo-sensitizer for optimization of the reaction conditions. A number of control experiments were performed, and no reaction was observed in the absence of amine or light (entries 13 and 14). Lowering the loading of PC (Table 3, entry 4), the absence of HCOOH or exposure to air (entries 11 and 12) were observed to decrease the substrate conversion of this reaction. Interestingly, using 1,8-diazabicyclo[5.4.0]undec-7-ene (DBU, entry 10, *E*_onset_^+/0^ = 0.60 V *vs.* Cp_2_Fe^+/0^), also led to excellent substrate conversion and good product yield comparable to what was obtained with tetramethylethylenediamine (TMEDA, entry 9, *E*_onset_^+/0^ = 0.11 V *vs.* Cp_2_Fe^+/0^, Fig. S9, ESI†). In contrast, the widely used photo-catalysts [Ru(bpy)_3_]Cl_2_ and *fac*-Ir(ppy)_3_ (Table 4, entries 15 and 16) showed little or no conversion under similar conditions.

**Table 4:** Visible-light-induced C–C bond formation for aryl halide

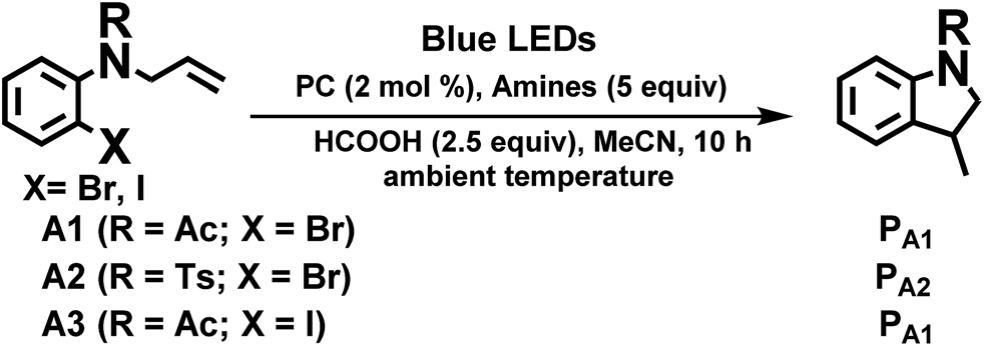				
Entry^*a*^	PC (substrate)	Amines	Conversion^*b*^/%	Yield^*b*^/%
1	**1b** (**A1**)	DIPEA	97	67
2	**4^Me^b** (**A1**)	DIPEA	97	64
3	**6^H^b** (**A1**)	DIPEA	96	64
4	**4^Me^b** (**A2**)	DIPEA	98	54
5	**1b** (**A2**)	DIPEA	99	<15
6	**6^H^b** (**A2**)	DIPEA	84	51
7^*c*^	**4^Me^b** (**A3**)	TEA	97	79
8	**4^Me^b** (**A1**)	TEA	91	64
9	**4^Me^b** (**A1**)	TMEDA	52	32
10	**4^Me^b** (**A1**)	DBU	96	72
11^*d*^	**4^Me^b** (**A1**)	DIPEA	85	54
12^*e*^	**4^Me^b** (**A1**)	DIPEA	62	28
13^*f*^	**4^Me^b** (**A1**)	—	0	0
14^*g*^	**4^Me^b** (**A1**)	DIPEA	0	0
15	Ru(bpy)_3_Cl_2_ (**A1**)	DIPEA	0	0
16	*fac*-Ir(ppy)_3_ (**A1**)	DIPEA	27	16

^*a*^Procedure: substrate 50 μmol, **PC** (2 mol%), amine (5 equiv.), HCOOH (2.5 equiv.) in 4 mL aqueous solution was degassed by nitrogen, and irradiated by blue light (12 W, *λ*_max_ = 462 nm) at ambient temperature for 10 h.

^*b*^Determined by ^1^H NMR spectroscopy by adding internal standard of 5,5′-dimethyl-2,2′-bipyridine.

^*c*^Irradiated for 4 h.

^*d*^Absence of HCOOH.

^*e*^Presence of air (no degassing).

^*f*^Absence of amine.

^*g*^Absence of light.

The radical cyclization of the alkyl bromides, **B1** and **B2**, catalysed by **1b**, **4^Me^b** or **6^H^b** proceeded smoothly, with reasonable to excellent substrate conversion and product yields. The yield of cyclization of **B1** was improved to up to 90% (entry 5, Table 3) when formic acid was added and **6^H^b** was used as a photo-catalyst (PC). When 2,2,6,6-tetramethyl-1-piperidinyloxy (TEMPO) was added, the reaction was totally inhibited, indicating the involvement of a radical intermediate in the reaction (entry 12).

The photo-catalytic reaction could be initiated from the oxidative quenching of 4^Me^b* with aryl/alkyl halides. This is because the excited state reduction potential of 4^Me^b* (*E*(Ir^IV/III*^)_pc_ = –1.51 V (*vs.* SCE), Table S3, Fig. S9†) can allow a direct one electron reduction of aryl/alkyl halides by 4^Me^b*, leading to carbon–halogen (σ*(C–X)) bond cleavage to give alkyl radical in the case of sp^3^ carbon or radical anion intermediate for sp^2^ carbon.^86^ Subsequent reactions of the alkyl radical or radical anion intermediate with C(sp^2^)–H bond lead to C–C bond formation. An aminium radical cation generated from the oxidation of amine by [**4^Me^b**]^+^^83^,^85^ could serve as an electron donor to complete the reductive process. In the case of [(ppy)_2_Ir(dtbbpy)]PF_6_,^83^ its triplet excited state ([(ppy)_2_Ir(dtbbpy)]^+^*) reacts with DIPEA *via* a reductive quenching mechanism to generate Ir(ii) species (*E*(Ir^III/II^)_pc_ = –1.51 V (*vs.* SCE)^1^, Fig. S9, ESI†), which initiates the subsequent reducing catalytic reaction (Table 5).

**Table 5:** Visible-light-induced C–C bond formation of alkyl bromide

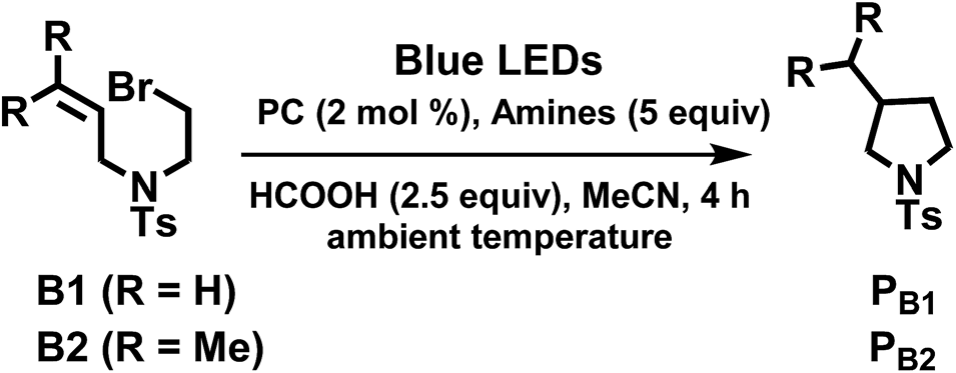				
Entry^*a*^	PC	Amines	Conversion^*b*^/%	Yield^*b*^/%
1	Ru(bpy)_3_Cl_2_	DIPEA	19	5
2	*fac*-Ir(ppy)_3_	DIPEA	90	72
3	**1b**	DIPEA	99	85
4	**4^Me^b**	DIPEA	99	76
5	**6^H^b**	DIPEA	99	90
6	**4^Me^b**	TEA	99	72
7	**4^Me^b**	TMEDA	84	55
8	**4^Me^b**	DBU	99	64
9	**4^Me^b**	—	0	0
10^*c*^	**4^Me^b**	DIPEA	27	15
11^*d*^	**4^Me^b**	DIPEA	0	0
12^*e*^	**4^Me^b**	DIPEA	0	0
13^*f*^	**4^Me^b**	DIPEA	99	59

^*a*^Entry 1–12: R = H (substrate **B1**); procedure: substrate 50 μmol, PC (2 mol%), amine (5 equiv.) and HCOOH (2.5 equiv.) in 4 mL MeCN solution was degassed by nitrogen, and irradiated by blue light (12 W, *λ*_max_ = 462 nm) at 25 °C.

^*b*^Determined by ^1^H NMR spectroscopy by adding an internal standard of 5,5′-dimethyl-2,2′-bipyridine.

^*c*^Absence of HCOOH (20 equiv.).

^*d*^Absence of light.

^*e*^Presence of TEMPO (radical trapping reagent, 2 equiv.).

^*f*^Entry 13: R = Me (substrate **B2**).

Interestingly, modifying the *N*-substituent of bis-NHC ligand from an alkyl group to a glucose moiety renders photo-catalyst **6^H^c** soluble in aqueous media. At the outset, we examined the **6^H^c**-catalysed reductive cyclization of **B1** in a mixture of H_2_O/MeOH (3 : 1) with ascorbic acid as reductant, but both the substrate conversion and product yield were low. The use of diisopropylethylamine (DIPEA) as reductant instead of ascorbic acid and addition of tetrabutylammonium chloride improved the conversion and yield to 98% and 49%, respectively. Increasing the vol% of methanol in aqueous solution to 75% leads to 99% conversion and 87% product yield. To the best of our knowledge, this is the first example of visible-light-driven radical cyclization for synthesis of pyrrolidine in aqueous media (Table 6).

**Table 6:** Visible-light-induced radical cyclization in aqueous solution

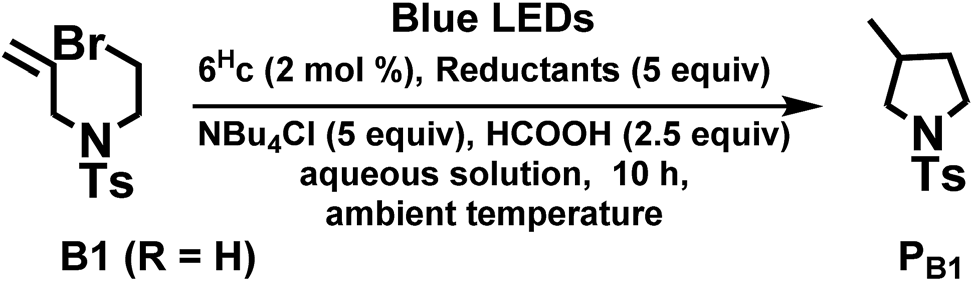				
Entry^*a*^	Solvents (H_2_O/MeOH)^*b*^	Reductant	Conversion^*c*^/%	Yield^*c*^/%
1	3/1	Ascorbic acid	23	10
2^*d*^	3/1	Ascorbic acid	20	14
3	3/1	DIPEA	98	49
4^*d*^ ^,^^*e*^	3/1	DIPEA	90	21
5^*d*^	3/1	DIPEA	79	31
6	1/1	DIPEA	99	64
7	1/3	DIPEA	99	87
8	0/1	DIPEA	99	66

^*a*^Procedure: substrate 50 μmol, **6^H^c** (2 mol%), reductant (5 equiv.), ^*n*^Bu_4_NCl (5 equiv.) in 4 mL aqueous solution was degassed by nitrogen, and irradiated by blue light (12 W, *λ*_max_ = 462 nm) at 25 °C.

^*b*^Solvent system used is water/methanol in volume ratio (v/v).

^*c*^Determined by ^1^H NMR spectroscopy by adding internal standard of 5,5′-dimethyl-2,2′-bipyridine.

^*d*^Absence of HCOOH.

^*e*^Absence of ^*n*^Bu_4_NCl.

### Visible-light-driven CO_2_ reduction

There is a surge of interest in developing photo-catalytic CO_2_ reduction using earth abundant metal complexes as catalysts and luminescent cyclometalated Ir(iii) complexes particularly *fac*-Ir(ppy)_3_ as PC. Nevertheless, several recent reports drew attention to the instability of *fac*-Ir(ppy)_3_, which is a challenge for achieving efficient light-driven CO_2_ reduction in the long run.^55^,^57^,^63^ In view of the good photo-stability of **4^Me^b**, we investigated the visible-light-driven CO_2_ reduction by utilizing **4^Me^b** in combination with the recently reported [Co(TPA)Cl]Cl complex.^57^

A CO_2_-saturated MeCN/triethylamine solution (4 : 1, v/v; 4 mL) containing catalytic amounts of **4^Me^b** and [Co(TPA)Cl]Cl was irradiated by blue LEDs (12 W) for a specified time period, and the evolved gases were separated and identified by GC-TCD equipped with a molecular sieve column. The volume of H_2_ and CO gases were calculated by using CH_4_ as the internal standard.

**Fig. 7:**
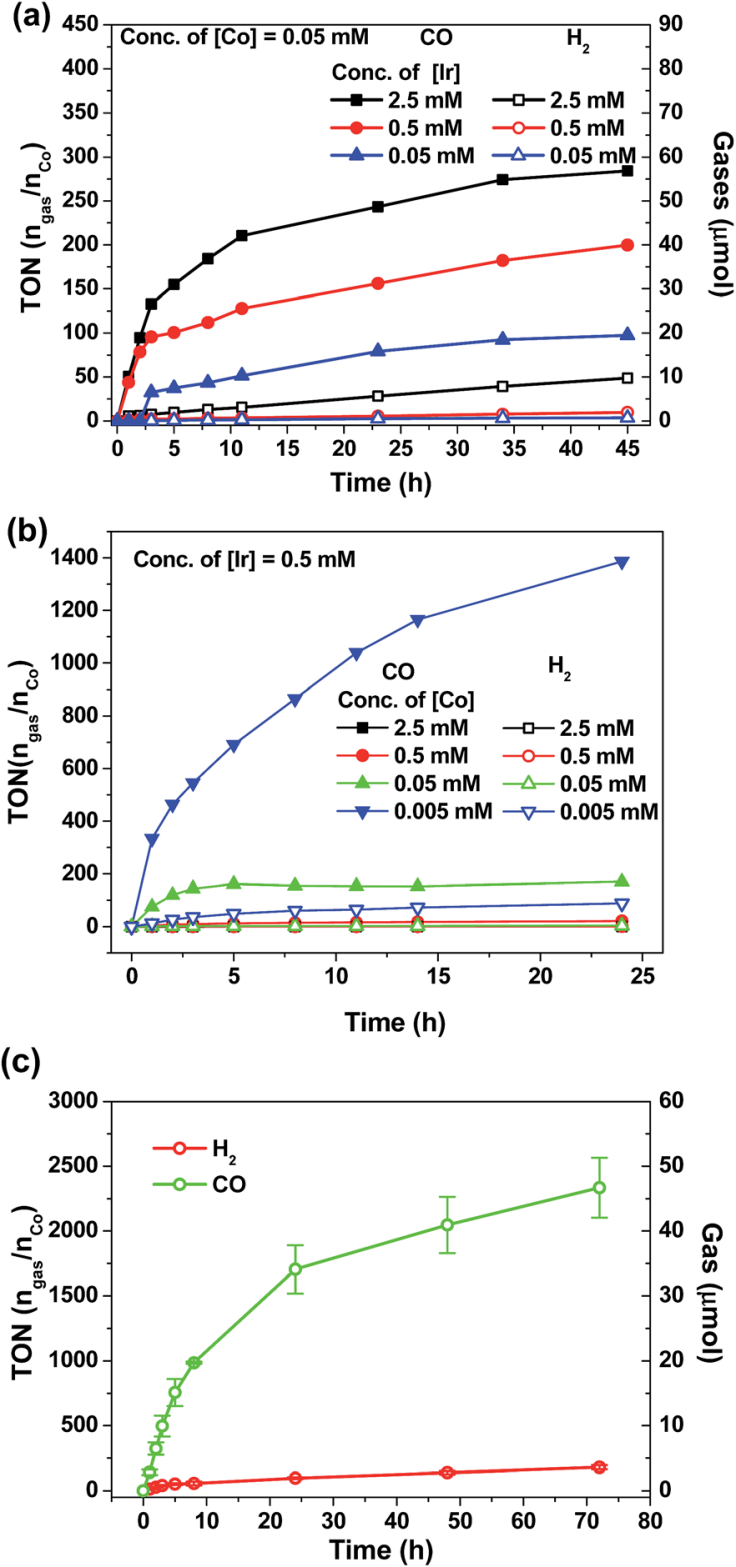
TON value and amounts of gases (CO and H_2_) generation from CO_2_ in a CO_2_-saturated MeCN/TEA (4/1, v/v, 4 mL in total) solution as a function of irradiation time: concentration dependence of (a) PC **4^Me^b** and (b) catalyst [Co(TPA)Cl]Cl; (c) a solution containing 0.005 mM [Co(TPA)Cl]Cl, 0.5 mM **4^Me^b** was irradiated using blue LEDs (12 W) based on the averaged results from three parallel reaction runs.

As shown in Fig. 7a and b, the amount of CO and H_2_ generated from the reaction mixture is found to show strong dependence on the concentrations of **4^Me^b** and Co(ii) catalysts. Particularly, the highest TON value of over 5000 can be accomplished at 0.5 μM of Co(ii), while no generation of gases is found at low concentration of Co(ii) (50 nM; Fig. S11, ESI†). Similarly, only negligible amount of product gases can be detected after irradiation for 24 h when [Ir] (0.005 mM) is lower than [Co] (0.05 mM). With the representative system containing **4^Me^b** (0.5 mM) and Co(ii) (0.005 mM), (Fig. 7c) the visible-light-driven CO_2_ reduction in three parallel reaction runs gives TON (CO) > 2400 (conversion of about 18 mL of CO_2_ into 1 mL of CO) with excellent selectivity in generating CO over H_2_ in the gaseous phase (>95%) after reaction for 72 h. This result is better than for the system utilizing *fac*-Ir(ppy)_3_ as PC, which reveals only TON (CO) > 900 and selectivity (CO) of 85% under similar reaction conditions.^57^

In order to confirm the roles of catalysts in photo-driven CO_2_ reduction, several control experiments were performed (Table S5, ESI†). Firstly, in the absence of Co(ii) complex, no CO gas was observed in a CO_2_-saturated MeCN/TEA (4 : 1, v/v; 4 mL) solution after irradiation for 24 h. On the other hand, in the absence of light, sacrificial amine or PC, the reaction mixture only gives negligible amounts of CO. To ascertain the catalytic role of the Co(ii) complex in the reaction, mercury was added to the reaction mixture in order to exclude the possibility of CO generation from heterogeneous Co nanoparticles. To specify, 29.2 μmol of CO (0.714 mL, TON 146) could be generated from the solution with [Ir] (0.5 mM), [Co] (0.05 mM) and elementary Hg (1 mL) after irradiation for 18 h, and this result is comparable with that using the solution mixture without Hg (28.4 μmol of CO formed, TON 142).

### Cellular imaging and cell viability assay

We have a long-standing interest in luminescent transition metal complexes, particularly those supported by NHC ligands that display anti-cancer activities.^9^,^16^,^87^–^90^ The luminescence would allow direct monitoring of cellular uptake and tracking of cellular location in cancer cells by fluorescence microscopy, and such properties of transition metal NHC complexes have been demonstrated to be useful in the elucidation of the anti-cancer mechanism of action. In this work, in view of their favourable photophysical properties, the *in vitro* cytotoxicity of the bis-NHC Ir(iii) complexes against HeLa cells was investigated by MTT assay. As shown in Table S6 (ESI†), the Ir(iii) complexes display potent cytotoxicity, with IC_50_ values ranging from 0.5 to 56.1 μM depending on the lipophilicity and structures of the Ir(iii) complexes. Those complexes with butyl groups on the bis-NHC ligand are more cytotoxic than those with glucose units.

**Fig. 8:**
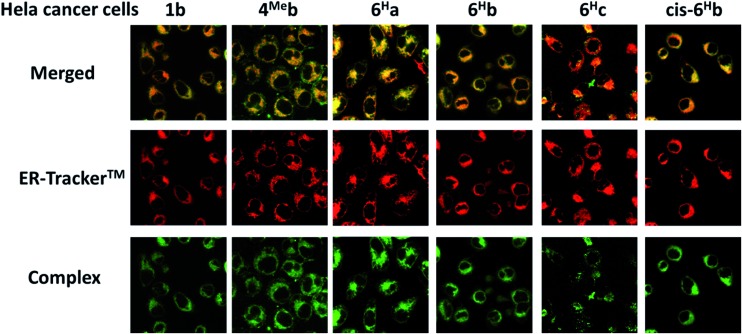
Fluorescence microscopy images of HeLa cancer cells incubated with the Ir(iii) NHC complexes: merged (top), ER-tracker™ (middle) and complexes (bottom). Complexes were excited at 340 nm using an emission filter of 510 nm. ER-tracker™ was excited at 546 nm using an emission filter of >580 nm.

Since complexes **1b**, **4^Me^b**, **6^H^a**, **6^H^b** and **6^H^c** demonstrate outstanding photophysical properties *i.e.* high quantum yield with long-lived electronic excited states, cellular imaging of these complexes in HeLa cells were performed. After treatment of human cervical cancer cells (HeLa) with the Ir(iii) complexes for 15 min, strong green/yellow luminescence was observed in the cytoplasm (but not nucleus) of cancer cells, as revealed by fluorescence microscopy (Fig. 8). Co-localization analyses indicate that the emission of these complexes are mainly localized in the endoplasmic reticulum (ER), which is stained by red-emissive ER-specific ER-Tracker™; a high Pearson's correlation coefficient (*R*) between the emissions of complexes and ER-Tracker™ is found (for example, **6^H^b** shows a high *R* value of 0.80). Consistently, these complexes do not accumulate in other organelles such as lysosome or mitochondria, as shown by the poor co-localization of the emissions of the complexes with the emission of Lyso-Tracker® and Mito-Tracker® respectively (Fig. S13, ESI†). Noticeably, **1b^+^** (both the counter anions of triflate and chloride) was found in our laboratory to be specifically localized in the ER of cancer cells (Fig. S14†), but not, as reported elsewhere, in mitochondria.^73^ With the specific accumulation of the complexes in ER, the cytotoxic properties of the complexes may originate from the induction of ER stress^88^ and immunogenic cell death.^91^

## Discussion

This work reveals the potential impact and usefulness of bis-NHC ligands for the development of robust metal photo-catalysts. Compared with the well-known complexes *fac*-Ir(ppy)_3_, this series of bis-NHC Ir(iii) complexes exhibits: (i) outstanding photo-stability under visible light irradiation; (ii) good photo-catalytic performance for several photo-electrochemically reactions; and (iii) long-lived emissive excited states.

### Stability of NHC metal complexes

The most important feature of metal–ligand bonding between transition metals and NHCs can be rationalized as the σ-coordination (sp^2^-hybridized lone pair electron) from NHCs. The contribution of both π-back-bonding into carbene p-orbital and π-donation from the carbene p-orbital might be considered as less significant for NHC metal complexes. These behaviours are similar to the coordination characteristics of phosphines, but NHCs are in general better electron-donors than phosphines. Thus, the stronger metal–ligand interaction renders NHC–metal coordination less labile than metal–phosphine bonding and the NHC complexes are more thermally stable.^92^ The distinct electronic properties and coordination chemistry of NHCs can also lead to improved catalytic activity of the metal complexes, owing to the increased catalyst stability and consequently lower rates of catalyst decomposition.^68^ In the previous section, the studies on the photo-stability of **4^Me^b** and *fac*-Ir(ppy)_3_ have revealed the outstanding stabilization contribution from bis-NHC carbene ligands. Thus this might be one reason that explains the better performance for photo-catalytic reactions using the present bis-NHC Ir(iii) complexes as PC.

### Role of bis-NHC ligands in tuning emission energy and lifetimes

A comparison between the photophysical data of bis-NHC Ir(iii) complexes with two notable Ir(iii) complexes (*fac*-Ir(ppy)_3_ and (ppy)_2_Ir(acac) (**1d**)) will be helpful in understanding the role of NHC on the strong absorptivities, high emission efficiencies and long lifetimes of the present bis-NHC Ir(iii) complexes. On the basis of DFT calculations on **1a** as the representative example (Fig. S4, ESI†), the lowest-energy absorption bands of **1a**, **1d** and *fac*-Ir(ppy)_3_ are ascribed to the HOMO → LUMO transitions, the energy of which is sensitive to the charge of ancillary ligands. The transition energy of *fac*-Ir(ppy)_3_ and **1d** with the negatively charge ancillary ligands (C^N and acac) are quite close with wavelengths of 416 and 414 nm respectively. However, for **1a** with the neutral ancillary ligand (bis-NHC), the HOMO → LUMO transition is markedly hypsochromically shifted to 369 nm, which is consistent with the experimental observations. Fig. S10 (ESI†) shows the surfaces and the energies of HOMO and LUMO of **1a**, *fac*-Ir(ppy)_3_ and **1d**. On the whole, the HOMO contains comparable components of iridium and C^N ligand, and the LUMO is mainly localized on the C^N ligand. Thus, the transition can be assigned as an admixture of metal-to-ligand charge transfer (MLCT) and ligand centered (LC) π–π* transition, which is in accordance with the assignments reported in the literature.

The different amounts of metal character in the frontier molecular orbitals of **1a**, **1d** and Ir(ppy)_3_, as deduced by TD-DFT calculations, can account for the photophysical properties and long emission lifetimes of the Ir(iii) NHC complexes. As shown in Fig. S10 (ESI†), *fac*-Ir(ppy)_3_ and **1d** have similar energy levels in HOMO and LUMO (around –4.5 eV and –2.0 eV). By simple substitution of the negatively charged ancillary ligands (C^N and acac) in Ir(ppy)_3_ and **1d** by neutral NHC ligand, **1a** is found to show lower energy levels of HOMO and LUMO (about –5.2 eV and –2.4 eV). This can be explained by the less electron-donating effect of the neutral NHC ligand compared to the negatively charged auxiliary ligands, as well as a certain contribution of the stabilization of dπ(Ir) by the π-acceptor orbitals of NHC ligand of **1a**.^68^ As a result, the energy level of the HOMO of **1a** is 700 mV lower than for **1d**, which coincides with the experimental observation that the oxidation potential of **1a** is 410 mV more positive than that of **1d**. On the other hand, the transition energy of HOMO → LUMO in **1a** is estimated to be 0.3 eV larger than in Ir(ppy)_3_ and **1d**, which may account for the blue shift of low-energy absorption of **1a** (369 nm *vs.* 410 nm), as compared to that of **1d** and Ir(ppy)_3_, in UV-Vis absorption spectra. Notably, TD-DFT calculation reveals that frontier molecular orbitals of **1a** show a smaller metal character than those of **1d** (Ir character in the HOMO of **1a** and **1d** are found to be ∼30 and ∼40% respectively), and hence the triplet excited states of **1a** are likely to have smaller metal character. This would probably slow down the spin–orbit coupling, resulting in slower radiative and non-radiative T_1_ → S_0_ decay for **1a**. As a result, **1a** shows a longer emission lifetime than **1d**.

### Triplet excited states for photo-catalytic reactivity

In addition to the photo-stability and strong absorptivity in the visible light region, the long-lived triplet excited states, especially their propensity to lose an electron, are to a large extent believed to be crucial for the photo-catalytic properties of the complexes. The long triplet excited state lifetime of **4^Me^b** is beneficial for bi-molecular photochemical reactions allowing the electron-transfer pathways to have sufficient time to take place prior to the decay of the excited state to the ground state.

Considering the fact that the excited state of [Ru(bpy)_3_]^2+^ (*E*(Ru^II*/I^) = 0.77 V *vs.* SCE) has a more powerful oxidative potential than for **4^Me^b** (*E*(Ir^III*/II^) = 0.51 V *vs.* SCE, Table S3†) and no radical cyclization products are obtained when using [Ru(bpy)_3_]^2+^, the photo-catalytic route *via* reductive quenching cycle is not feasible. The photo-catalytic reaction would possibly be initialized *via* an oxidative quenching cycle of excited states of the photo-catalyst. By carefully examining the transient-absorption spectra of triplet state of **4^Me^b** in MeCN, newly generated long-lived species were observed by using higher energy laser beams (Fig. S8b–g†). These long-lived species were found to be increased in the presence of substrate **A1**, and could be quenched by DIPEA. This long-lived species might be Ir(iv), which is generated by single electron transfer from [**4^Me^b**]* to **A1**. The calculated excited-state reduction potential of **4^Me^b** (*E*(Ir^IV/III*^) = –1.51 V *vs.* SCE, Table S3†) reveals that **4^Me^b** is not a stronger photo-reductant than *fac*-Ir(ppy)_3_ (*E*(Ir^IV/III*^) = –1.73 V *vs.* SCE).^37^,^93^ However, the performance of **4^Me^b** in visible-light-driven radical cyclization and CO_2_ reduction prove to be better than for *fac*-Ir(ppy)_3_. For example, the conversion of substrates **A1**/**B1** to indoline/pyrrolidine by **4^Me^b** (97%/99%) are higher than those by *fac*-Ir(ppy)_3_ (27%/90%). Therefore, there should be other reasons for the good performance of **4^Me^b** in photo-catalysis. Plausible reasons could be: (i) the generated Ir(iv) [**4^Me^b**]^+^ (*E*(Ir^IV/III^) = 0.96 V *vs.* SCE, Table S3†) are more easily reduced by amines than [*fac*-Ir(ppy)_3_]^+^ (*E*(Ir^IV/III^) = 0.77 V *vs.* SCE); and (ii) the higher photo-stability of **4^Me^b** compared with that of *fac*-Ir(ppy)_3_.

Consequently, the excellent photo-stability, strong absorptivity, long lifetimes and the photo-ionization behaviours of our bis-NHC Ir(iii) complexes possibly enable the photolysis of long-lived excited states of Ir(iii)* to Ir(iv) more easily and thus promote the radical cyclization in a catalytic cycle.

### Diversities of biological activities of Ir(iii) complexes with different *N*-substituents on NHC ligands

Although *N*-substituents of NHC ligands are found to show negligible effects on the luminescent properties of the bis-NHC Ir(iii) complexes, they have a determinant role on the physical properties and hence the anti-cancer activities of the complexes. For example, complexes with *N*-butyl substituents on the bis-NHC ligands generally display lower IC_50_ values than those with *N*-methyl substituents (Table S6 in ESI†). This is probably attributed to the increase in lipophilicity of the complexes, resulting in better permeability through cellular membrane and higher accumulation of the complexes in cancer cells. The fast cellular uptake of the complexes is supported by the strong luminescence observed in HeLa cells after incubation of the cells with the complexes for 15 min (Fig. 8 and S12 in ESI†). On the other hand, the glucose-functionalized NHC ligands give the complexes good aqueous solubility and a more hydrophilic nature, leading to likely slower cellular uptake as well as reduced cytotoxicity toward cancer cells. It is noteworthy that the complexes accumulate in cellular ER as revealed by fluorescence microscopy images of the co-staining experiments. This can consequently induce ER stress^88^ and immunogenic cell death, which probably accounts for the high cytotoxicity of the complexes. Further manipulations of the functionalities on NHC ligands of the Ir(iii) complexes may realize the development of a new class of diagnostic and/or therapeutic agents.

## Conclusions

A new series of cyclometalated Ir(iii) complexes bearing bis-NHC ligands has been demonstrated as strongly luminescent materials and promising photo-catalysts for visible-light-driven radical cyclization and CO_2_ reduction and as biological theranostic agents. Owing to the high stability of the Ir–C^NHC^ bond, these bis-NHC Ir(iii) complexes show excellent photo-stability compared with widely-used PC, such as *fac*-Ir(ppy)_3_ and [Ru(bpy)_3_]^2+^. With long-lived triplet excited states and rich photoredox properties, **4^Me^c** can undergo photo-ionization in aqueous media as supported by transient absorption experiments, while **4^Me^b** is found to be a more effective catalyst for radical cyclization and CO_2_ reduction than *fac*-Ir(ppy)_3_ under visible-light irradiation. Interestingly, water-soluble **6^H^c**, which contains a glucose moiety on the bis-NHC ligands, has been demonstrated as the first PC for the synthesis of pyrrolidine in aqueous media. In addition, through modulations of the chemical structures of the cyclometalated ligands and/or *N*-substituents on the bis-NHC ligands, the Ir(iii) complexes have been found to show different luminescent properties as well as anti-cancer activities, indicating the potential of the complexes as theranostic agents.

## Author contribution

Chen Yang, Faisal Mehmood, Tsz-Lung Lam, Yuan Wu, Chi-Shun Yeung, Xiangguo Guan, Kai Li, Clive Yik-Sham Chung, Cong-Ying Zhou and Taotao Zou carried out all the experiments and performed data analysis. Chen Yang, Chi-Ming Che and Sharon Lai-Fung Chan designed the experiments, analysed the data and wrote the manuscript. All authors reviewed the manuscript.

## Supplementary Material

Supplementary information

Crystal structure data
