# Supplementary material for: Stable luminescent iridium(iii) complexes with bis(N-heterocyclic carbene) ligands: photo-stability, excited state properties, visible-light-driven radical cyclization and CO2 reduction, and cellular imaging
Source: Chem Sci. 2016 Jan 20;7(5):3123–36. doi: 10.1039/c5sc04458h (PMC6003676; doi:10.1039/c5sc04458h)
Supplement: Supplementary file 1 [file SC-007-C5SC04458H-s001.pdf]

Electronic Supplementary Material (ESI) for Chemical Science.
This journal is © The Royal Society of Chemistry 2016

Chemical Science RSCPublishing

ARTICLE

Bis(N-heterocyclic carbene) ligands-containing luminescent iridium(III) complexes: photo-stability, excited state properties, visible-light-driven radical cyclization and CO_2 reduction, and cellular imaging†

Chen Yang,[ab] Faisal Mehmood,[a] Tsz Lung Lam,[cd] Sharon Lai-Fung Chan,*[cd] Yuan Wu,[a] Chi-Shun Yeung,[a] Xiangguo Guan,[a] Kai Li,[ab] Clive Yik-Sham Chung,[a] Cong-Ying Zhou,[ab] Taotao Zou,[a] and Chi-Ming Che*[ab]

[a] State Key Laboratory of Synthetic Chemistry, Institute of Molecular Functional Materials, HKU-CAS Joint Laboratory on New Materials and Department of Chemistry, The University of Hong Kong, Pokfulam Road, Hong Kong, China
[b] HKU Shenzhen Institute of Research and Innovation, Shenzhen, China
[c] The Hong Kong Polytechnic University Shenzhen Research Institute, Shenzhen, PR China
[d] Department of Applied Biology and Chemical Technology, The Hong Kong Polytechnic University, Hung Hom, Hong Kong, China

TABLE OF CONTENTS

**Supporting figures and tables in text (except those of photo-catalysis and biological studies):**	1
**Fig. S1**. ^{1}H NMR spectra of **7b** (left, CD_3CN) and **7c** (right, CD_3OD) at various low temperatures.	1
**Fig. S2**. Comparison of ^{1}H NMR of **6^Hb** (CD_3CN) and **cis-6^Hb** (CD_3CN); the ESI-MS pattern for **cis-6^Hb** and **6^Hb**.	1
**Fig. S3.** Perspective view of crystal structures of **4^{Me}b** (left) and **7b** (right)	1
**Fig. S4.** UV/Vis absorption and emission spectra of a) **1a** – **1c**, b) **$6^{H/F}$b** – **9b** c) **4^{Me}a**, **4^{Me}b**, **4^{Hex}b**, **5b** and **L5**, d) solvent dependence of **4^{Me}b**; The calculated UV-Vis profile of e) **1a**, f) **Ir(ppy)$_3$** and g)**1d** in DCM	2
**Fig. S5.** Cyclic voltammograms of **1a**, **4^{Hex}b, 6^Fb**, and **7b** – **9b**.	2
**Fig. S6** Time resolved spectra of **5b** and **6^Hc**	3
**Fig. S7** Kinetic studies of tr-abs and tr-em spectra of **4^{Me}c** and presence of acetone.	3
**Fig. S8** Transient absorption spectra of **6^Hc**, **4^{Me}b** by different energy of laser.	4
**Fig. S9** Cyclic voltammograms of amines and reaction equation and potentials of Ir(III)* and amines.	4
**Fig. S10** MO diagram of **1a, 1d** and *fac*-**Ir(ppy)$_3$** based on the optimized ground state geometries.	5
**Table S1.** Selected bond length and angles of **4^{Hex}b, 6^Hb, 7b** and cis-**6^Hb**.	5
**Table S2.** Crystal data and structure refinement for crystals **4^{Hex}b, 6^Hb, 7b** and **cis-6^Hb**	6
**Table S3.** Electrochemical data and excited state redox properties of complexes **1** – **9**.	6
**Experimental Section**	7
General procedure for preparation of iridium(III) complexes **1** – **9**:	8
Charaterization of complexes **1** – **9**:	8-11
Synthesis and characterization of **HL3**, **HL4**, **HL5** and **HL9.**	12-16
Synthesis and characterization of bis-NHC ligands: **DiGluBr$_2$**.	16-17
**Visible-light-driven radical cyclization**	17
Synthesis of substrates and general procedure for visible-light-induced radical cyclization.	17
**Table S4**. Characterization of substrates and products in photocatalysis.	18-19
**Visible-light-driven CO_2 Reduction**	19
Procedure and measurements of CO_2 reduction.	19
**Table S5.** Control experiments of CO_2 reduction.	19
**Fig. S11** Plots of moles of gases produced versus concentration of **Co** and **4^{Me}b**.	20
**Fig. S12** Stern-volmer properties of **4^{Me}b** in the presence of **[Co(TPA)Cl]Cl**.	20
**Biological Studies**	21
**Table S6.** IC_{50} (μM) values of test compounds towards HeLa cell line.	21
**Fig. S13.** Fluorescence microscopic analysis of **1b, 4^{Me}b**, **6^Hb** and **1bCl** in cell lines with various trackers**.**	22-26
**Fig. S14.** Fluorescence microscopic analysis of **1b**, **4^{Me}b** and **6^Hb** with ER-tracker^{TM}, and **1bCl** with Mito-tracker® in cancer cells.	27-28
**References**	29
**NMR spectra**	30-46

Supporting figures and tables in text:

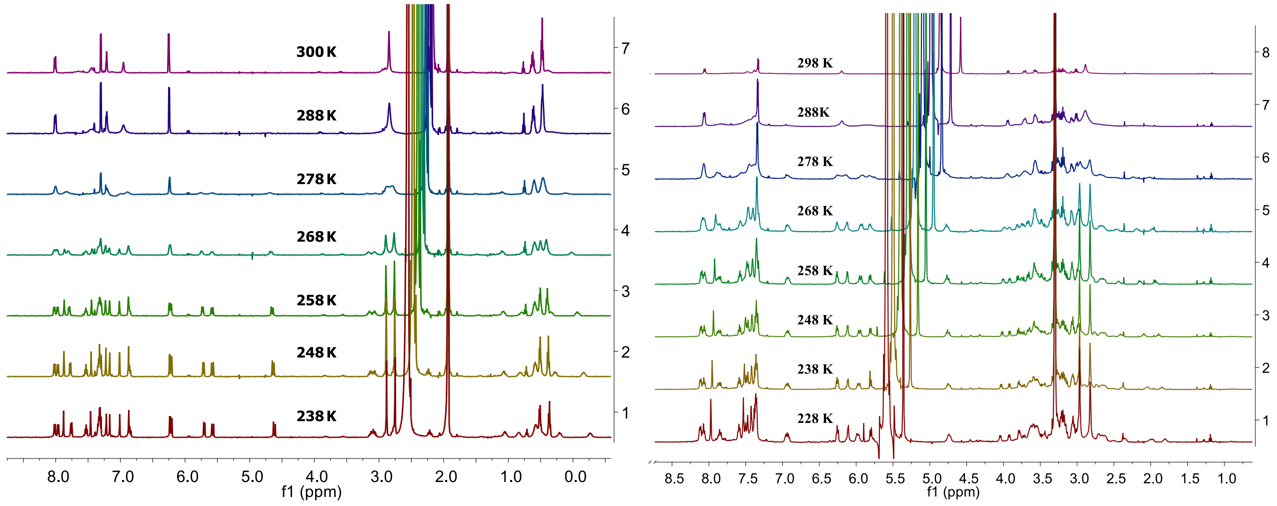

Fig. S1. ^{1}H NMR spectra of **7b** (left, CD_3CN) and **7c** (right, CD_3OD) in various low temperatures.

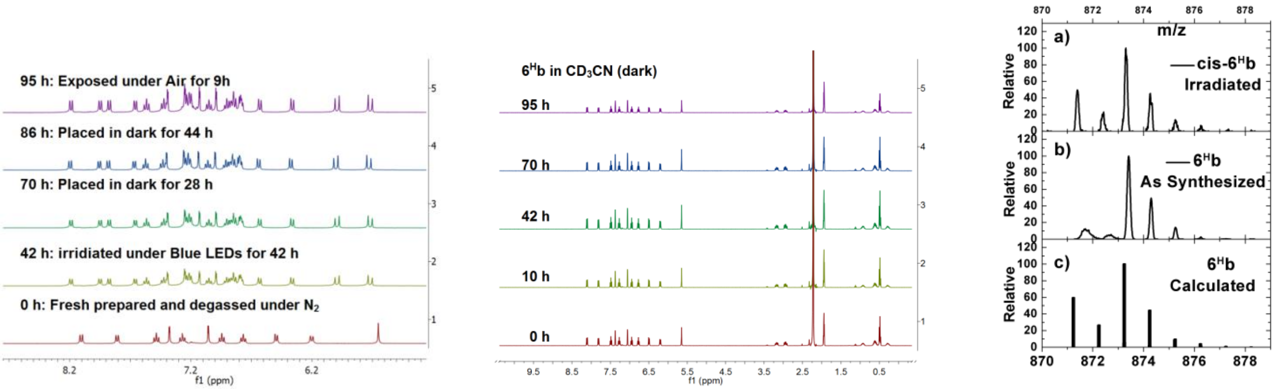

Fig. S2. Left: ^{1}H NMR spectral traces of **6^{H}b** in degassed deuterated acetonitrile (CD_3CN) upon irradiation by blue light (12 W) at room temperature. After irridiation, the formation of **cis-6^{H}b** is stable in solution, even exposed to air; **Middle**: ^{1}H NMR spectra traces of **6^{H}b** in deuterated acetonitrile in dark. **Right**: a) the ESI-MS pattern for parent ion **cis-6^{H}b** (the final solution from NMR tube); b) the ESI-MS pattern for parent ion of as-prepared **6^{H}b**; c) the calculated isotope pattern for the cation ion of **6^{H}b** (ESI-MS: *m/z* 873.5 [*M-OTf*]$^+$; Calcd. for $C_{41}H_{40}IrN_6S_2$, 873.2.).

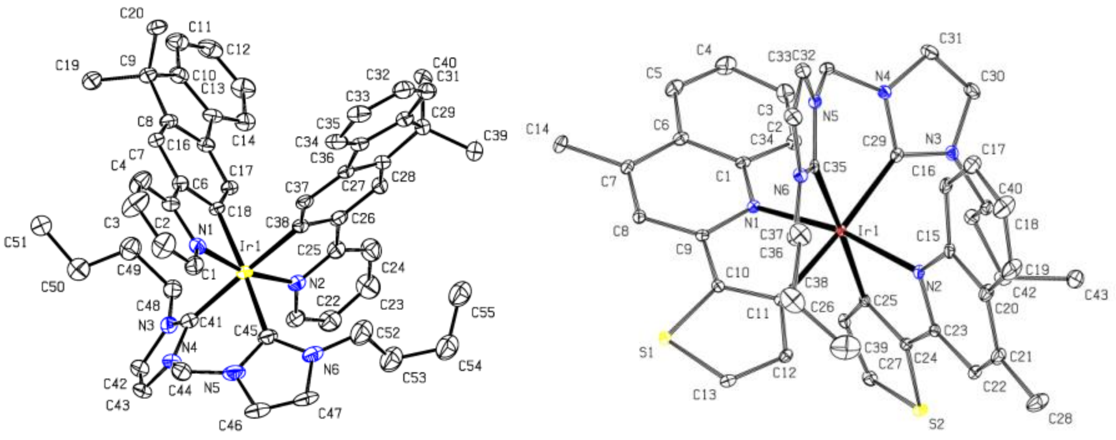

Fig. S3. Perspective view of **4^{Me}b** (left) and **7b** (right) (all hydrogen atoms, the CH_3CN solvent molecules and counteranions (PF_6^- or OTf^-) are omitted for clarity). Thermal ellipsoids are drawn at the 30% probability level.

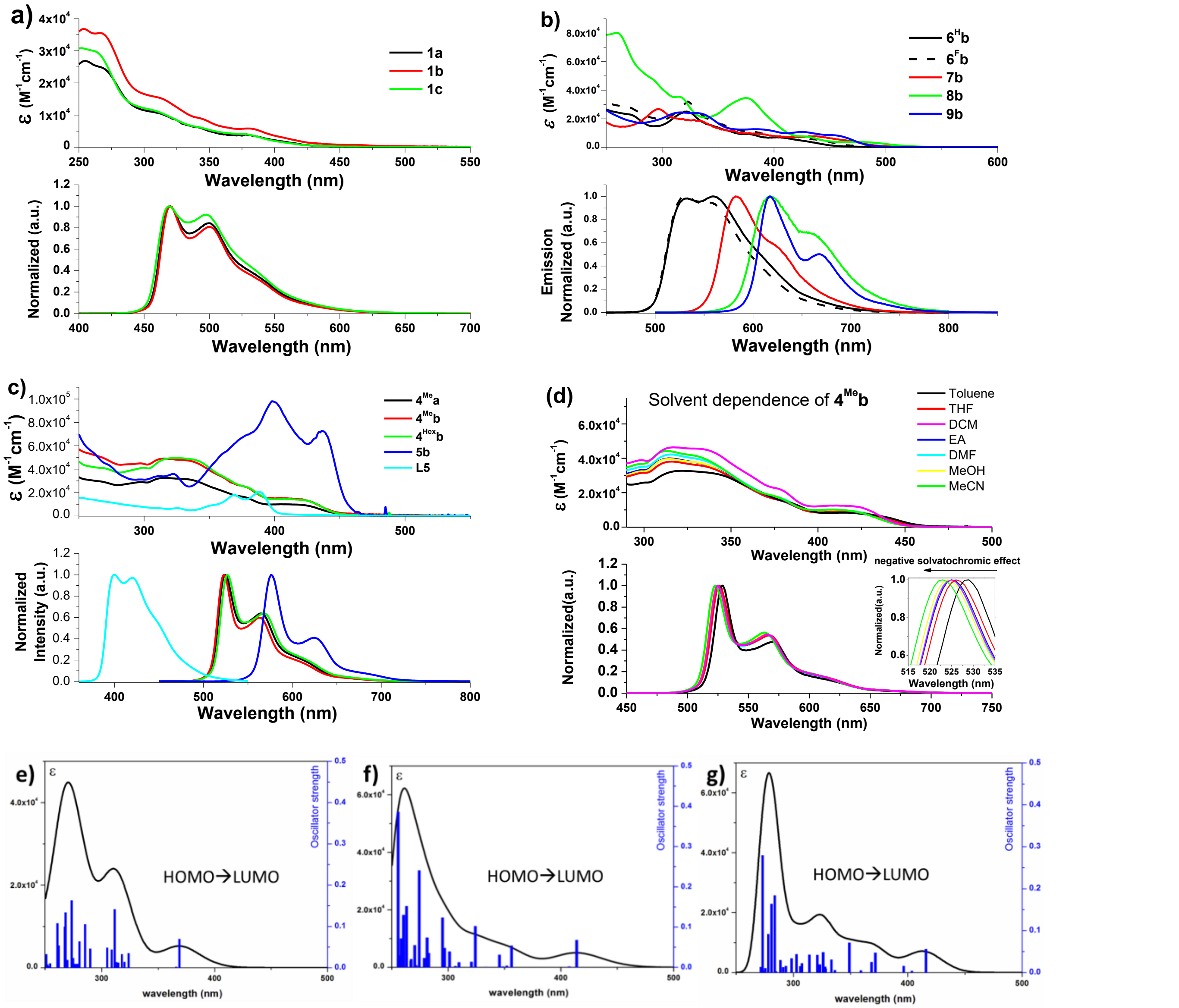

Fig. S4. UV/Vis absorption (top) and emission (bottom) spectra of solutions of a) **1a** – **1c**, b) **6**$^{H/F}$**b** – **9b** and c) **4**^{Me}**a**, **4**^{Me}**b**, **4**^{Hex}**b**, **5b** and **L5** in degassed DCM (concentration of 2.0$\times$10^{-5} M) at 298 K, d) solvent dependence of **4**^{Me}**b**; The calculated UV-Vis profile of e) **1a**, f) **1d** and g) *fac*-Ir(ppy)$_3$ in DCM.

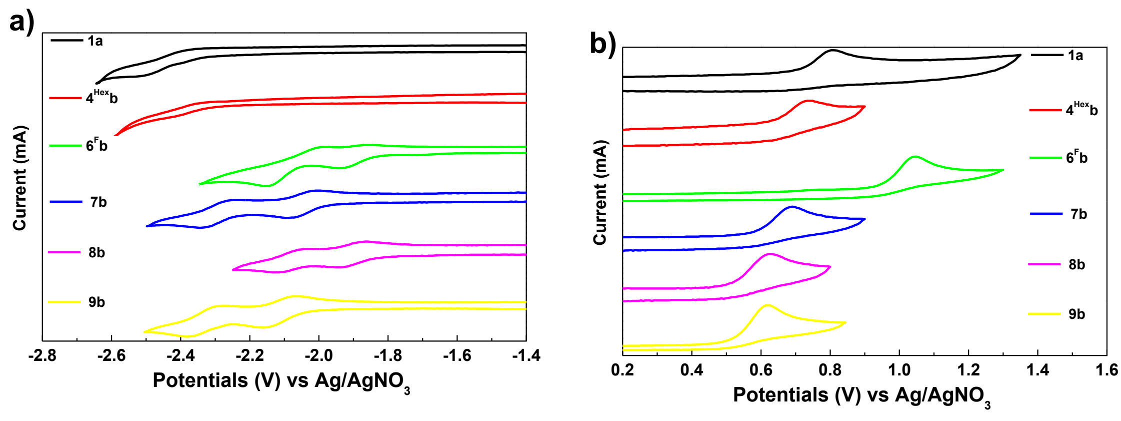

Fig. S5. Cyclic voltammograms of solution of **1a**, **4**^{Hex}**b**, **6**^{F}**b**, and **7b** – **9b** in acetonitrile with $^{n}Bu_4NPF_6$ (0.1 M) as supporting electrolyte. Conditions: working electrode, glass-carbon, scan rate: 100 mVs^{-1}.

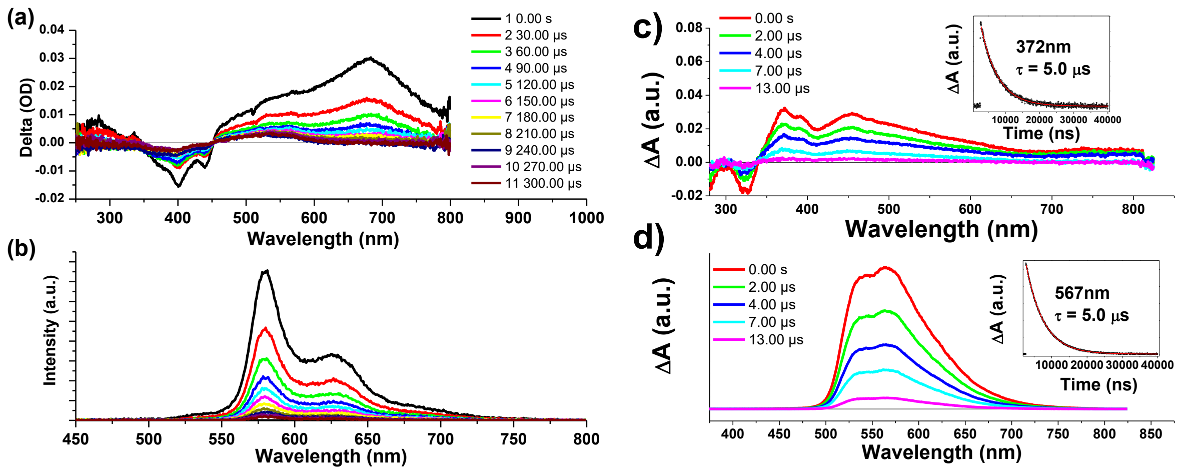

Fig. S6 Time resolved spectra: a) transient absorption; b) transient emission spectra of **5b** in degassed DCM; c) transient absorption; d) transient emission spectra of **6^{H}c** in degassed water recorded at specified time after laser pulse excitation (355 nm, laser energy: 5 mJ; beams area: 0.5 cm²) at 298 K.

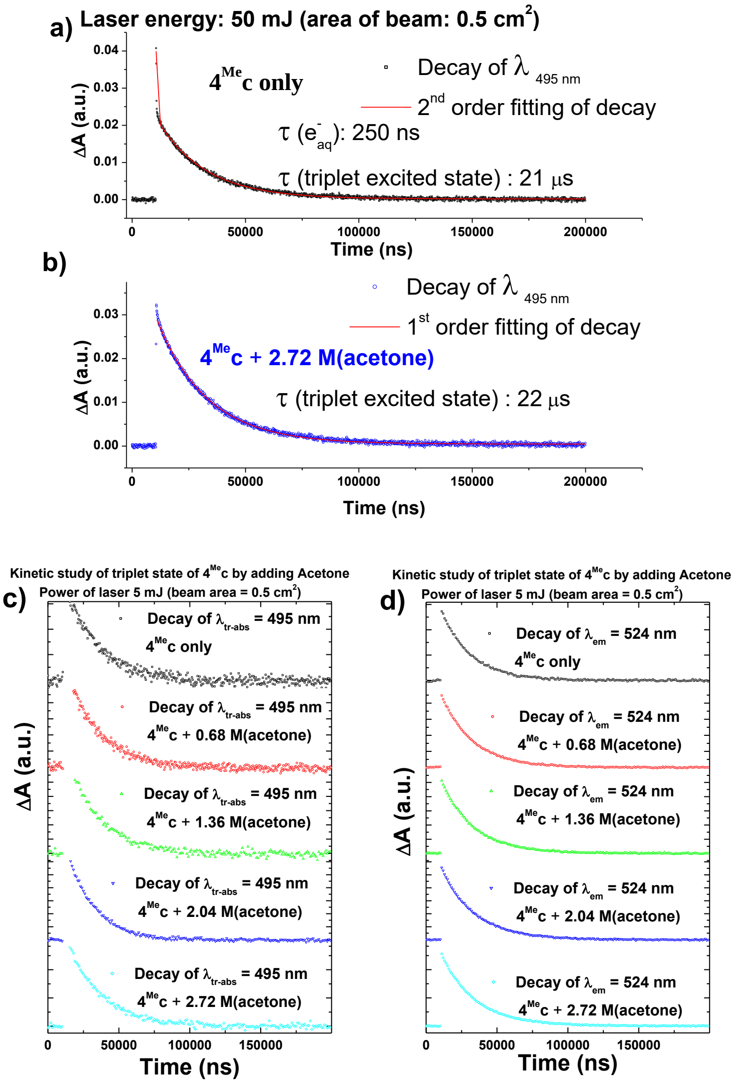

Fig. S7 Kinetic studies of tr-abs spectra of **4^{Me}c** at λ = 495 nm: a) **4^{Me}c** only and b) **4^{Me}c** and in the presence of acetone (2.72M); c) tr-abs of λ (495 nm) and d) tr-em of λ (524 nm) of **4^{Me}c** by adding specified acetone.

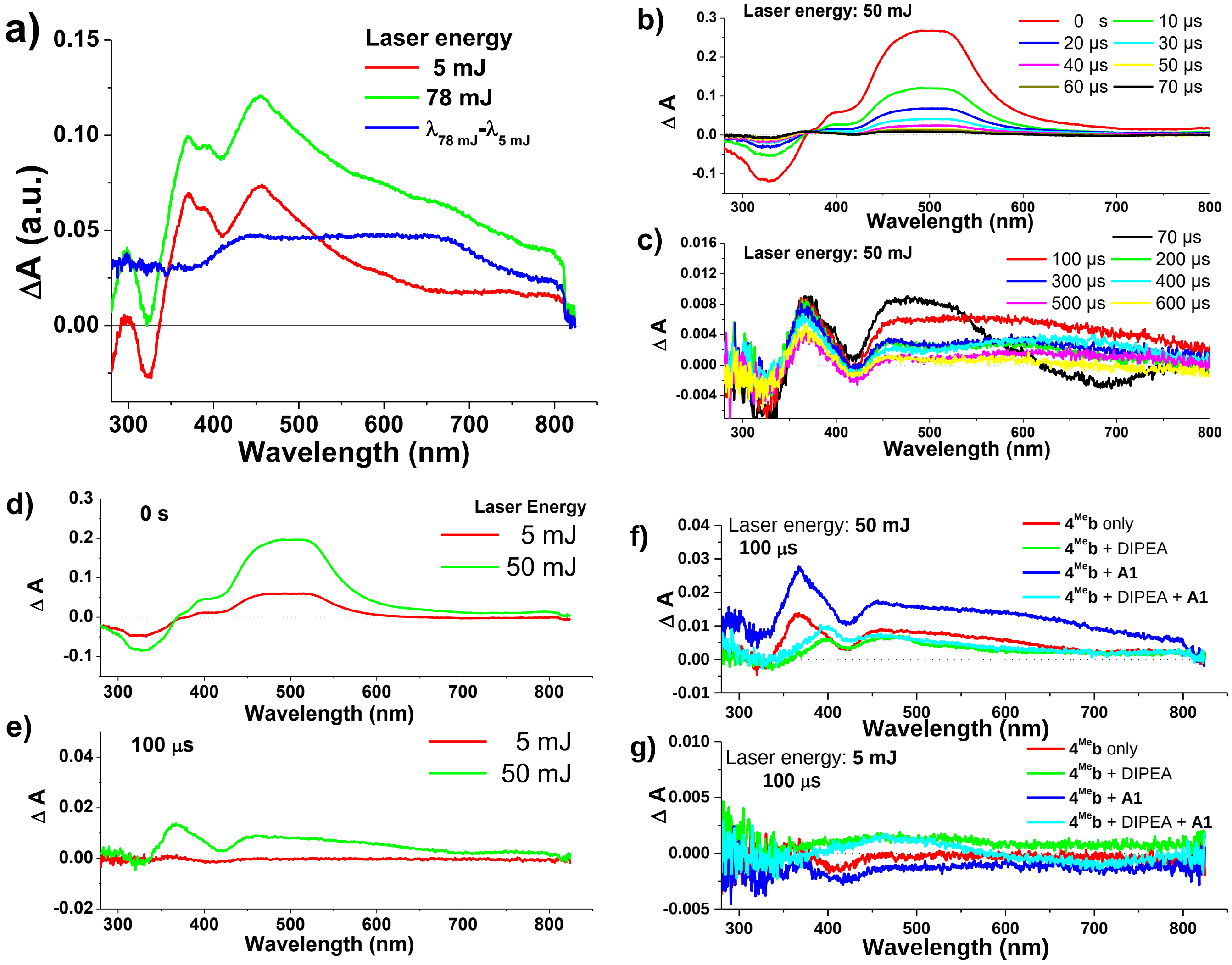

Fig. S8 a) Transient absorption spectra of **$6^{H}c$** in water by different energies of laser beams; b) and c) time-resolved transient absorption spectra of **$4^{Me}b$** in MeCN under 50 mJ laser beams; d) and e) transient absorption spectra of **$4^{Me}b$** after specified time excitation from different energy of laser beams; f) and g).transient absorption spectra of **$4^{Me}b$** in MeCN, in presence of DIPEA, substrate **A1** and both after 100 μs upon specified energy of laser excitation.(laser beams area 0.5 cm^2, concentration of **$6^{H}c$** and **$4^{Me}b$**: 2×10^{-5} M; DIPEA and **A1**: 2×10^{-3} M (100 equiv. to **$4^{Me}b$**)).

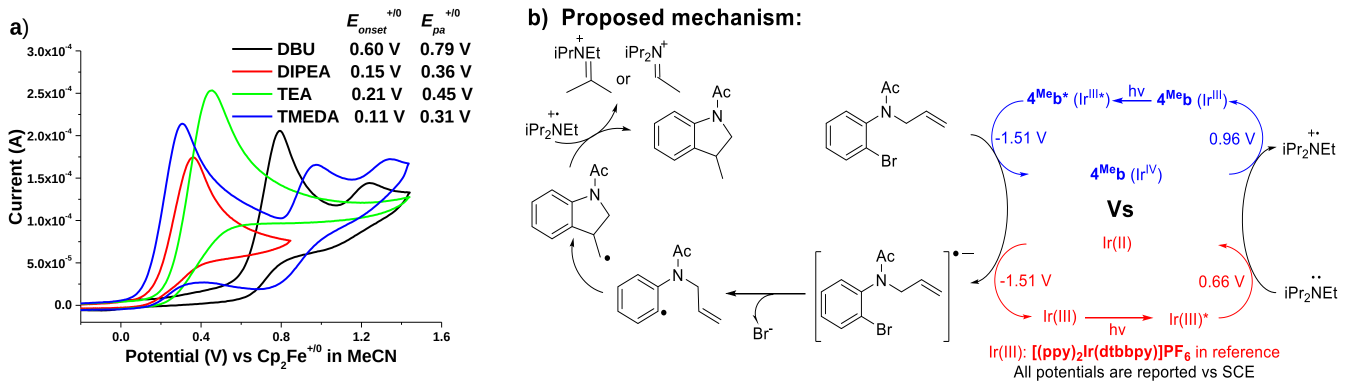

Fig. S9 a): Cyclic voltammograms of solution of **amines** in acetonitrile with $^{n}Bu_4NPF_6$ (0.1 M) as supporting electrolyte. Conditions: working electrode, glass-carbon, scan rate: 100 mVs^{-1}; b): mechanism for radical cyclization for substrate **A1**, the blue (proposed) /red (reference[1]) catalytic cycles represent the different quenching cycles.

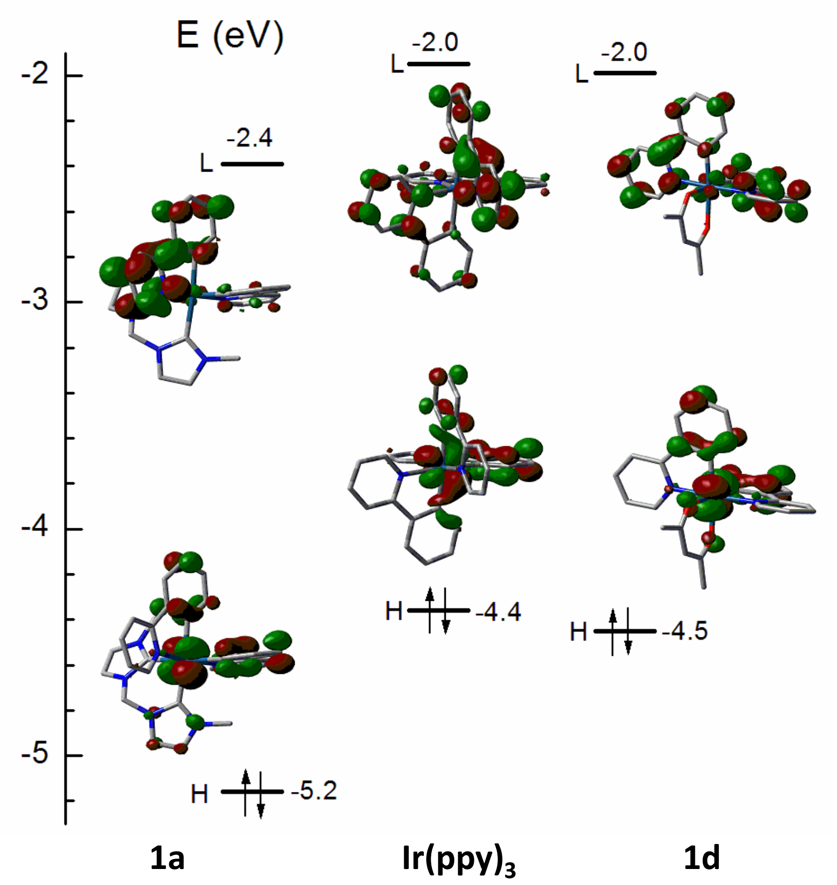

Fig. S10 MO diagram of **1a, Ir(ppy)$_3$** and **1d** based on the optimized ground state geometries (H for HOMO and L for LUMO).

Table S1. Selected bond length(Å) and angles (deg).

Complex	Bond length (Å)		Bond angles (deg)	
**4^{Me}b**	Ir1-C18	2.064(6)	C18-Ir1-C45	174.7(3)
	Ir1-C38	2.054(6)	C38-Ir1-C41	175.4(3)
	Ir1-C41$^{NHC\ a}$	2.139(7)	N1-Ir1-N2	167.8(2)
	Ir1-C45^{NHC}	2.112(7)	C18-Ir1-N1	79.5(2)
	Ir1-N1	2.074(6)	C38-Ir1-N2	80.1(2)
	Ir1-N2	2.053(6)	C41-Ir1-C45	84.7(3)
**6^{H}b**	Ir1-C1	2.077(2)	C1-Ir1-C31	176.38(9)
	Ir1-C14	2.054(2)	C14-Ir1-C27	174.27(9)
	Ir1-C27^{NHC}	2.128(2)	N1-Ir1-N2	167.85(8)
	Ir1-C31^{NHC}	2.105(2)	C1-Ir1-N1	79.17(9)
	Ir1-N1	2.071(2)	C14-Ir1-N2	79.47(8)
	Ir1-N2	2.067(2)	C27-Ir1-C31	85.10(9)
**7b**	Ir1-C11	2.0568(17)	C11-Ir1-C29	175.81(6)
	Ir1-C25	2.0440(17)	C25-Ir1-C35	173.60(6)
	Ir1-C29^{NHC}	2.0941(17)	N1-Ir1-N2	168.75(5)
	Ir1-C35^{NHC}	2.1080(17)	C11-Ir1-N1	78.63(6)
	Ir1-N1	2.1156(14)	C25-Ir1-N2	79.28(6)
	Ir1-N2	2.0894(14)	C29-Ir1-C35	85.58(6)
**cis-6^{H}b**	Ir1-C13	2.187(18)	C27-Ir1-C13	173.1(12)
	Ir1-N2	2.106(16)	C31-Ir1-N2	169.1(12)
	Ir1-C31^{NHC}	2.00(3)	N1-Ir1-N2	97.7(12)
	Ir1-C27^{NHC}	2.06(4)	C13-Ir1-N1	77.4(11)
	Ir1-N1	2.05(3)	C26-Ir1-N2	78.7(10)
	Ir1-N2	2.20(3)	C27-Ir1-C31	86.0(14)

[a] NHC refers to the bis-NHC carbene carbon atoms.

Table S2. Crystal data and structure refinement for crystals.

**Complex**	**4^{Me}b**	**6^{H}b**	**7b**	**cis-6^{H}b**
Empirical formula	$C_{57}H_{59}F_6IrN_7P$	$C_{42}H_{40}N_6O_3F_3S_3Ir$	$C_{44}H_{44}N_6O_3F_3S_3Ir$	$C_{42}H_{40}F_3IrN_6O_3S_3$
Formula weight	1179.28	1022.18	1050.23	1022.18
Temperature/K	100	100	115.04	100
Crystal system	monoclinic	orthorhombic	triclinic	triclinic
Space group	P21/n	Pbca	P-1	P-1
a/Å	14.9598(7)	13.3460(5)	11.6716(7)	15.8141(6)
b/Å	22.5882(11)	19.6302(7)	12.4594(8)	16.3895(6)
c/Å	16.6294(8)	30.1437(11)	14.7489(9)	18.8888(7)
α/degree	90	90	74.683(2)	67.0480(19)
β/ degree	112.4532(13)	90	86.255(2)	67.5521(17)
γ/ degree	90	90	83.689(2)	82.559(2)
Volume/Å3	5193.3(4)	7897.2(5)	2054.6(2)	4165.4(3)
Z	4	8	2	4
ρcalcg/cm^3	1.508	1.719	1.698	1.63
μ/mm^{-1}	5.826	8.566	3.465	8.12
F(000)	2384	4080	1052	2040
Crystal size/mm^3	0.03 × 0.02 × 0.01	0.1 × 0.05 × 0.05	0.354 × 0.222 × 0.18	0.2 × 0.15 × 0.03
Radiation	CuKα (λ = 1.54178)	CuKα (λ = 1.54178)	MoKα (λ = 0.71073)	CuKα (λ = 1.54178)
2Θ range for data collection/	6.96 to 134.94	5.86 to 135.22	5.12 to 50.06	5.46 to 108.86
Index ranges	-17 ≤ h ≤ 17, -26 ≤ k ≤ 26, -19 ≤ l ≤ 19	-14 ≤ h ≤ 15, -23 ≤ k ≤ 21, -28 ≤ l ≤ 35	-13 ≤ h ≤ 13, -14 ≤ k ≤ 14, -17 ≤ l ≤ 17	-16 ≤ h ≤ 16, -17 ≤ k ≤ 17, -19 ≤ l ≤ 19
Reflections collected	54353	144642	43882	50514
Independent reflections	8990 [Rint = 0.0746, Rsigma = 0.0472]	7057 [Rint = 0.0644, Rsigma = 0.0215]	7243 [Rint = 0.0250, Rsigma = 0.0151]	[R_{int} = 0.0819, R_{sigma} = 0.0759]
Data/restraints/parameters	8990/5/626	7057/0/525	7243/0/546	10044/828/996
Goodness-of-fit on F^2	1.049	1.127	1.012	1.036
Final R indexes [I ≥2σ (I)]	R_1 = 0.0647, wR_2 = 0.1706	R_1 = 0.0227, wR_2 = 0.0588	R_1 = 0.0123, wR_2 = 0.0307	R_1 = 0.1713, wR_2 = 0.3922
Final R indexes [all data]	R_1 = 0.0706, wR_2 = 0.1799	R_1 = 0.0228, wR_2 = 0.0590	R_1 = 0.0126, wR_2 = 0.0309	R_1 = 0.2365, wR_2 = 0.4436
Largest diff. peak/hole / e Å^{-3}	3.11/-1.43	0.79/-0.95	0.40/-0.50	9.71/-4.68

[a], $R_1 = \sum||F_o| - |F_c|| / \sum|F_o|$; [b]$wR_2 = [\sum w(|F_o| - |F_c|)^2 / \sum w|F_o|^2]^{1/2}$

Table S3. Electrochemical Data[a] and Excited State Redox Properties of Complexes **1**–**9**.

Complex	$E(Ir^{IV/III})$ [b] (V) *vs* $Cp_2Fe^{+/0}$	E $(Ir^{III/II})$ [c] (V) *vs* $Cp_2Fe^{+/0}$	$E_{0,0}$[d]	$E(Ir^{IV/III*})$ *(vs* $Cp_2Fe^{+/0}$ / SCE)[e]	$E(Ir^{III*/II})$ *(vs* $Cp_2Fe^{+/0}$ / SCE) [e]	E_g [f] (eV)	HOMO/LUMO [f] (eV)
**1b**	0.68 (0.80)	-2.39 (-2.63)	2.74	-2.06/-1.70	0.35/0.71	3.07	-5.48/-2.41
**2a**	0.48 (0.59)	-2.12 (-2.24)	2.43	-1.95/-1.59	0.31/0.77	2.60	-5.28/-2.68
**4^{Me}a**	0.60 (0.69)	-2.33 (-2.45)	2.47	-1.87/-1.51	0.14/0.50	2.93	-5.4/-2.47
**4^{Me}b**	0.60 (0.70)	-2.32 (-2.49)	2.47	-1.87/-1.51	0.15/0.51	2.92	-5.4/-2.48
**4^{Hex}b**	0.60 (0.69)	-2.33 (-2.46)	2.45	-1.85/-1.49	0.12/0.48	2.93	-5.4/-2.47
**5b**	0.62 (0.72)	-2.16 (-2.31)	2.24	-1.62/-1.26	0.08/0.44	2.78	-5.42/-2.64
**6^{H}b**	0.81 (0.92)	-2.09 (-2.21)	2.50	-1.69/-1.33	0.41/0.79	2.90	-5.61/-2.71
**6^{F}b**	0.87 (0.99)	-1.88 (-2.0)	2.50	-1.63/-1.27	0.67/1.03	2.75	-5.67/-2.92
**7b**	0.53 (0.63)	-2.03 (-2.13)	2.25	-1.72/-1.36	0.22/0.58	2.56	-5.33/-2.77
**8b**	0.46 (0.57)	-1.90 (-2.0)	2.15	-1.69/-1.33	0.25/0.61	2.62	-5.37/-2.64
**9b**	0.52 (0.62)	-2.02 (-2.16)	2.10	-1.58/-1.32	0.08/0.44	2.54	-5.55/-2.78

[a] Supporting electrolyte: 0.1 M $^nBu_4NPF_6$ in MeCN and $E(Cp_2Fe^{+/0})$ are recorded at the range from 0.05 – 0.06 (V) *vs* $AgNO_3$/Ag (0.1 M) in MeCN; [b] Values refer to onset of the anodic peak potential (E^{ox}_{onset}) and oxidation peak potential (E_{pa}) in parenthesis at 25 ºC for irreversible couples at a scan rate of 100 mV s^{-1}; [c] Values refer to onset of the cathodic peak potential (E^{red}_{onset}) and reduction peak potential (E_{pc}) in parenthesis for the irreversible reduction waves; [d] approximate zero-zero excitation energy $E_{0,0} = 1240/\lambda_{em}$ (onset of emission band at 25 ºC), [e] Calculations of approximate redox potentials of excited iridium(III) complexes: $E(Ir^{IV/III*}) = E(Ir^{IV/III}) - E_{0,0}$, $E(Ir^{III*/II}) = E(Ir^{III/II}) + E_{0,0}$ (eV).[2] [f] $E_g = E^{ox}_{onset} - E^{red}_{onset}$, HOMO (eV) = $-e(E^{ox}_{onset} + 4.8)$, and LUMO (eV) = $-e(E^{red}_{onset} + 4.8)$. [3]

Experimental Section

Photophysical measurements

UV-vis absorption spectra were recorded on a Hewlett-Packard 8453 diode array spectrophotometer. Photo-excitation and steady-state emission spectra were obtained on a SPEX Fluorolog-3 Model FL3-21 spectrofluorometer. Solution samples for measurements were degassed on a high-vacuum line in a two-compartment cell that consisted of a pyrex bulb (10 mL) and a quartz cuvette (path length: 1 cm) and was sealed from the atmosphere by a Bibby Rotaflo HP6 Teflon Stopper. The solutions were rigorously degassed by at least five successive freeze/pump/thaw cycles. Excited state lifetime measurements of solution samples were performed with a Quanta Ray DCR-3 pulsed Nd:YAG laser system (pulse output 355 nm, 8 ns). The emission signals were detected by a Hamamatsu R928 photomultiplier tube and recorded on a Tektronix TDS 350 oscilloscope, and analysed by using a program for the exponential fits. Φ_{PL} values were measured[4] relative to that of a degassed acetonitrile solution of Ru(bpy)$_3$(PF$_6$)$_2$ (bpy = 2,2′-bipyridine) (Φ_{ref} = 0.062) as a standard reference and calculated by: $\Phi_s = \Phi_r(B_r/B_s)(n_s/n_r)^2(D_s/D_r)$, where the subscripts *s* and *r* refer to sample and reference standard solutions, respectively, *n* is the refractive index of the solvents, *D* is the integrated intensity, and Φ is the luminescence quantum yield. The excitation intensity *B* was calculated by: $B = 1 - 10^{-AL}$, where *A* is the absorbance at the excitation wavelength and *L* is the optical path length (*L* = 1 cm in all cases). Errors for λ values (± 1 nm), τ (± 10 %), Φ (± 10 %) were estimated. Nanosecond time-resolved absorption and emission measurements were performed using a LP920-KS Laser Flash Photolysis Spectrometer (Edinburgh Instruments Ltd, Livingston, UK). The excitation source was 355 nm output from a Nd:YAG laser.

Cyclic voltammetry

Cyclic voltammetry measurements were performed on a Princeton Applied Research Model 273A potentiostat. The glassy-carbon electrode was polished with 0.05 μm alumina on a microcloth, sonicated for 5 min in deionized water, and rinsed with acetonitrile before use. A Ag/AgNO$_3$ (0.1 M in MeCN) electrode was used as reference electrode and a platinum wire as counter electrode. All solutions of samples were prepared in MeCN containing 0.1 mol dm^{-3} tetra(*n*-butyl)ammonium hexafluorophosphate (^{n}Bu$_4$NPF$_6$) as supporting electrolyte. The solutions were purged and maintained under Argon atmosphere. Scan rates were 100 mV s^{-1} and the potentials were reported with respect to the potential of Ag/AgNO$_3$. Ferrocene was used as internal reference and recorded with the potential at the range of 0.05 – 0.07 V for ferrocenium/ferrocene (Cp$_2$Fe$^{+/0}$) vs Ag/AgNO$_3$.

Computational details

Density functional theory (DFT) and time-dependent density functional theory (TDDFT) calculations have been performed to understand the geometries and the electronic structures of Iridium complexes (**1a, 1d)** using Gaussian 09 package.[4] PBE0[5]/6-31G*(lanl2dz)[6] was used for the geometry optimization and PBE0/6-31+G*(lanl2dz) was used for TDDFT calculations. The Solvent effects have been studied using self-consistent reaction field (SCRF) method based on PCM models.[7] The choice of solvents (dichloromethne， a dielectric constant e = 8.93) was based on the solvent media for experiments.

X-ray crystal-structure determination

Crystals of **4^{Me}b** (with PF$_6^-$ counter anion), **6^{H}b**, **cis-6^{H}b** and **7b** suitable for X-ray crystallography were obtained by slow diffusion of diethyl ether into DCM (**6^{H}b**), MeCN (**4^{Me}b** and **7b**) and chloroform (**cis-6^{H}b**) solution of these complexes, respectively. The X-ray diffraction data were collected on a Bruker X8 Proteum diffractometer except for **7b** (Bruker D8 Venture diffractometer). The crystal was kept at 100 K during data collection. The diffraction images were interpreted and the diffraction intensities were integrated by using the program SAINT. Multi-scan SADABS was applied for absorption correction. By using Olex2,[5] the structure was solved with the ShelXS[6] structure solution program using direct Methods and refined with the XL[6] refinement package using Least Squares minimization. The positions of the H atoms were calculated on the basis of the riding mode with thermal parameters equal to 1.2 times that of the associated C atoms and these positions participated in the calculation of the final R indices. In the final stage of least-squares refinement, all non-hydrogen atoms were refined anisotropically. Crystallographic parameters are summarized in Table S1. CCDC 1428476–1428479 contain the supplementary crystallographic data for this paper. The data can be obtained free of charge from The Cambridge Crystallographic Data Center via www.ccdc.cam.ac.uk/data_request/cif.

Materials for syntheses of complexes:

2-phenyl-6-(trifluoromethyl)-benzo[d]thiazole (**L6^F**), 2-phenylbenzo[d]thiazole (**L6^H**)[7] were synthesized according to literature procedure. 1,1'-methylenebis(3-methyl-1H-imidazol-3-ium) diiodide, 1,1'-methylenebis(3-butyl-1H-imidazol-3-ium) diiodide were prepared according to a modification of the literature method.[8] Iridium trichloride (IrCl$_3$) hydrate, lithium trifilate (LiOTf) and silver(I) oxide (Ag$_2$O) were purchased from commercial sources and were used as received without further purification. Deionized (DI) water was used in the experiment procedure.

Materials for synthesis of ligands: (HL3, HL4$^{Hex/Me}$, HL5 and HL9) :

Sodium carbonate, potassium carbonate, potassium fluoride (KF), ammonium chloride (NH_4Cl), potassium t-butoxide (KO^tBu), zinc chloride ($ZnCl_2$), magnesium, sodium hydride (60%), sodium acetate, iodine (I_2), bromine (Br_2), 1-bromohexane, 9-H-fluorene, glucose, acetic anhydride, 1H-imidazole, 2-bromopyridine, 2-bromoethanol, dibromomethane, 2-bromo-9-H-fluorene, 2-bromothiophene, isoquinolin-1-ol, boron trifluoride diethyl etherate ($BF_3{\cdot}Et_2O$), 4-methoxybenzaldehyde (4-MeOPhCHO), sulfuric acid (H_2SO_4), silver(I) oxide (Ag_2O), 1,2-*bis*(diphenylphosphino)ethane nickel(II) chloride (**Ni(dppe)Cl₂**), n-butyllithium in Hexane solution (2.4M, J&K), tetrakis(triphenylphosphine)palladium(0) [$Pd(PPh_3)_4$], [1,1'-bis(diphenylphosphino)ferrocene]dichloropalladium(II) [$Pd(dppf)Cl_2$], magnesium sulfate ($MgSO_4$), 1-bromopyrrolidine-2,5-dione (**NBS**), dimethylformamide (DMF), dichloromethane (DCM), ethylacetate (EA), 2-methoxyethanol, chloroform ($CHCl_3$), carbon tetrachloride (CCl_4), diethylether (Et_2O), acetonitrile (MeCN), methanol (MeOH), ethanol (EtOH), toluene, tetrahydrofuran (THF) and hexane (Hex) were purchased from commercial sources and were used as received without further purification. Deionized (DI) water was used in the experiment procedure.

General procedure for preparation of iridium(III) complexes 1–9:

Characterization for synthesis: ^{1}H NMR spectra were recorded using deuterated solvent on a Bruker Avance DPX-300, AV-400, or DRX-500 Fourier-Transform NMR spectrometer; chemical shifts are reported relative to tetramethylsilane. (Solvents: $CDCl_3$, CD_3CN and CD_3OD; chemical shifts:*δ*, ppm; *J*, Hz). Positive-ion electrospray Ionization (ESI) mass spectra were obtained on a Finnigan LCQ quadrupole ion trap mass spectrometer. Elementary analysis of the new complexes was performed on a Flash EA 1112 elemental analyzer at the Institute of Chemistry, the Chinese Academy of Sciences.

These complexes were synthesized from refluxing of **HC^N** ligand with $IrCl_3$ in aqueous solution of 2-methoxyethanol (75% in volume) for overnight (12–18 hours), the resulting precipitate was filtrated, washed by water, ethanol and diethyl ether and dried by air. The dichloro-bridged iridium complexes $[(C\text{^}N)_2IrCl]_2$ were used without further purification. A typical procedure is described as follows (example of **1**): In a 50 mL two-neck round-bottomed flask was **[(ppy)₂IrCl]₂** (150 mg, 0.14 mmol, 1 eq), 1,1'-methylenebis(3-butyl-1H-imidazol-3-ium), $2I^-$ (159 mg, 0.308 mmol, 2.2 eq), and silver(I) oxide (136 mg, 0.588 mmol, 4.2 eq) in 2-methoxyethanol (volume: 10 mL) to give a black suspension. The reaction was refluxing at 120 °C for overnight, 2-methoxyethanol (volume: 10 mL) was removed under vacuum pump. 40 mL of DCM was added and filtrated through celite, the resulting filtrate was washed by Lithium triflate aqueous solution (0.1 g/mL) 2 times. Organic layer was dried by $MgSO_4$. After organic solvent was removed, the residue was prepared to purify by chromatographic column, and eluted by DCM/MeCN (V/V = 20/3). After removed organic solvent, the yellowish green solid was in good yield of 82% complex of **1b**: 210 mg, 0.231 mmol). δ_H (400 MHz, $CDCl_3$) = 8.14 (2 H, d, *J* 5.4 Hz), 7.93 (2 H, d, *J* 8.1 Hz), 7.82 – 7.75 (4 H, m, *J* 9.1 Hz), 7.61 (2 H, d, *J* 7.6 Hz,), 7.01 (2 H, t, *J* 6.4 Hz), 6.88 (2 H, t, *J* 7.3 Hz), 6.81 – 6.72 (4 H, m), 6.31 (2 H, s), 6.27 (2 H, d, *J* 7.4 Hz), 3.46 – 3.37 (2 H, m,), 3.19 – 3.10 (2 H, m), 0.91 (2 H, qd, *J* 12.2, 6.7 Hz), 0.71 (4 H, dt, *J* = 14.1, 7.2 Hz), 0.59 (6 H, t, *J* = 6.9 Hz), 0.32 ppm (2 H, qd, *J* = 13.1, 7.2 Hz). δ_F (376 MHz, $CDCl_3$) = -78.23 ppm (s). ESI-MS: *m/z* 761.6 [*M-OTf*]$^+$; Calcd. for $C_{37}H_{40}IrN_6$, 761.3. Elemental Analysis (%): calcd. for $C_{38}H_{40}F_3IrN_6O_3S{\cdot}H_2O$: C, 49.18; H, 4.56; N, 9.06; found: C, 48.90, H, 4.52; N, 8.56. Complex **1b·Cl**: **1b** with anion of Cl^-: **1b** with anion of OTf^- was refluxing in MeOH, and the anion was exchanged by adding Amberlite IRA-401 (Cl) ion exchange resin. Elemental Analysis (%): calcd. for $C_{37}H_{40}ClIrN_6{\cdot}(H_2O)_3$: C, 52.25; H, 5.45; N, 9.88; found: C, 52.42, H, 5.40; N, 9.85.

Complex 1a:

The synthesis procedure of bis-NHC carbene Ir(III) complexes with counter anions of PF_6^- is similar to that of trifilate Ir(III) complexes. **1a** (not purified by chromatographic column) was dissolved into MeOH and excess of NH_4PF_6 was added into above solution, resulting precipitate was filtrated, washed by water, MeOH and Et_2O, and dried by air. The solid was prepared to purify by chromatographic column, and eluted by DCM/MeCN (V/V = 10/1 to 5/1). δ_H (400 MHz, CD_3CN) = 8.25 (2 H, d, *J* 5.7 Hz), 8.05 (2 H, d, *J* 8.2 Hz), 7.88 (2 H, t, *J* 7.6 Hz), 7.72 (2 H, d, *J* 7.8 Hz), 7.08 (2 H, t, *J* 6.3 Hz), 6.91 (4 H, m), 6.76 (2 H, t, *J* 7.3 Hz), 6.34 (2 H, d, *J* 7.4 Hz), 5.97 (2 H, s), 2.80 ppm (6 H, s). δ_F (376 MHz, CD_3CN) = -72.91 ppm (d, *J* 706.3 Hz). ESI-MS: *m/z* 677.1 [*M-PF₆*]$^+$; Calcd. for $C_{31}H_{28}IrN_6$, 677.2. Elemental Analysis (%): calcd. for $C_{31}H_{28}PF_6IrN_6{\cdot}H_2O$: C, 44.34; H, 3.60; N, 10.01; found: C, 44.50, H, 3.58; N, 9.60.

Complex 1c:

Typical procedure for synthesis of Ir(III) complexes bearing D-glucose on terminal of bis-NHC carbene ligand (**[(C^N)₂Ir(dig)]OTf**), refers to the procedure of **6ᴴc**. Yield: 13%. δ_H (400 MHz, CD_3OD) = 8.27 (2 H, d, *J* 4.8 Hz), 8.13 (2 H, d, *J* 8.2 Hz), 7.91 (2 H, t, *J* 7.6 Hz), 7.74 (2 H, d, *J* 7.7 Hz), 7.38 (4 H, t, *J* 14.6 Hz), 7.13 (2 H, t, *J* 6.3 Hz), 6.88 (2 H, t, *J* 7.4 Hz), 6.74 (2 H, t, *J* = 6.7 Hz), 6.27 – 6.22 (2 H, m), 6.12 (2 H, s), 4.46 – 4.06 (3 H, m), 3.97 (1 H, d, *J* 7.8 Hz), 3.90 – 3.76 (4 H, m), 3.75 – 3.39 (6 H, m), 3.28 – 2.97 (9 H, m), 2.87 (1 H, d, *J* 11.4 Hz), 2.48 (1 H, t, *J* 8.6 Hz), 2.15 (3 H, s), 2.13 – 1.95 (2 H, m) ppm. δ_F (376 MHz, CD_3OD) δ = -80.09 ppm. ESI-MS: *m/z* 1061.4 [*M-OTf*]$^+$; Calcd. for $C_{45}H_{52}IrN_6O_{12}$, 1061.1.

Complex 2a:

Yield: 60%. δ_H (400 MHz, CD_3CN) = 8.10 (2 H, d, *J* 5.8 Hz), 7.75 (2 H, t, *J* 7.6 Hz), 7.59 (2 H, d, *J* 8.0 Hz), 7.35 – 7.23 (4 H, m), 6.96 (2 H, s), 6.86 (2 H, t, *J* 6.6 Hz), 6.19 (2 H, d, *J* 4.7 Hz,), 5.97 (2 H, s), 2.90 ppm (6 H, s). δ_F (376 MHz, CD_3CN) = -72.90 (d, *J* 706.2 Hz). δ_P (162 MHz, CD_3CN) = -144.53 (sept, *J* 706.4 Hz). ESI-MS: *m/z* 689.3 [*M-PF*$_6$]$^+$; Calcd. for $C_{27}H_{24}IrN_6S_2$, 689.1. Elemental Analysis (%): calcd. for $C_{27}H_{24}F_6IrN_6PS_2{\cdot}H_2O$: C, 38.07; H, 3.08; N, 9.87; found: C, 38.18, H, 2.88; N, 7.45.

Complex 3a:

Yield: 19%, this complex is difficult to purify. After twice chromatograph purification procedures, the desired crude product was purified from growing crystals by diffusion diethylether into a solution of DCM. δ_H (400 MHz, CD_3CN) = 8.10 (2 H, d, *J* 5.5 Hz), 7.78 (2 H, t, *J* 7.7 Hz), 7.56 (2 H, d, *J* 8.0 Hz), 7.31 (2 H, s), 7.27 (2 H, d, *J* 5.0 Hz), 7.14 (2 H, s), 6.99 (4 H, s), 6.88 (2 H, t, *J* 6.4), 6.32 (2 H, s,), 5.98 (2 H, s), 3.02 ppm (6 H, s). δ_F (376 MHz, CD_3CN) = -72.90 ppm (d, *J* 706.4 Hz). δ_P (162 MHz, CD_3CN) = -144.53 ppm (sept, *J* 706.2 Hz). ESI-MS: *m/z* 853.1 [*M-PF*$_6$]$^+$; Calcd. for $C_{35}H_{28}IrN_6S_4$, 853.1. Elemental Analysis (%): calcd. for $C_{35}H_{28}F_6IrN_6PS_4{\cdot}(CH_2Cl_2)_{0.5}$: C, 40.98; H, 2.81; N, 8.08; found: C, 40.38, H, 2.86; N, 8.05.

Complex 4^{Hex}b:

Yield: 66%. δ_H (400 MHz, $CDCl_3$) = 8.20 (2 H, d, *J* 5.9 Hz), 8.05 (2 H, d, *J* 8.0 Hz), 7.85 (4 H, dd, *J* 15.1, 5.1 Hz), 7.57 (2 H, s), 7.20 – 7.10 (8 H, m), 7.03 (2 H, t, *J* 6.0 Hz), 6.78 (2 H, d, *J* 1.8 Hz), 6.60 (2 H, s), 6.37 (2 H, s), 3.45 – 3.24 (4 H, m), 1.89 – 1.81 (4 H, m), 1.81 – 1.67 (4 H, m), 1.09 (6H, dt, *J* 13.5, 6.7 Hz), 1.05 – 0.94 (10 H, m), 0.88 (2 H, t, *J* 7.0 Hz), 0.85 – 0.79 (4 H, m, *J* 6.6 Hz), 0.75 (8 H, t, *J* 7.2 Hz), 0.72 – 0.64 (10 H, m), 0.56 (8 H, t, *J* 7.2 Hz), 0.51 (6 H, t, *J* 7.2 Hz), 0.44 – 0.36 (2 H, m), 0.33 – 0.22 (2 H, m) ppm. δ_F (376 MHz, $CDCl_3$) = -78.21 ppm. ESI-MS: *m/z* 1274.0 [*M-OTf*]$^+$; Calcd. for $C_{75}H_{96}IrN_6$, 1273.7. Elemental Analysis (%): calcd. for $C_{76}H_{96}F_3IrN_6O_3S{\cdot}(H_2O)$: C, 63.35; H, 6.86; N, 5.83; found: C, 62.77, H, 6.97; N, 5.88.

Complex 4^{Me}a, with anion of OTf$^-$:

Yield of 47%. δ_H (400 MHz, CD_3CN) = 8.35 (2 H, d, *J* 5.6 Hz), 8.20 (2 H, d, *J* 8.2 Hz), 7.97 (2 H, s), 7.93 (2 H, s), 7.41 (2 H, d, *J* 7.2 Hz), 7.30 – 7.25 (4 H, m), 7.22 (4 H, t, *J* 7.8 Hz), 7.14 (2 H, s), 6.90 (2 H, s), 6.77 (2 H, s), 6.00 (2 H, s), 2.82 (6H, s), 1.43 (6 H, s), 1.35 ppm (6 H, s). δ_F (376 MHz, CD_3CN) = -79.32 ppm. ESI-MS: *m/z* 909.4 [*M-OTf*]$^+$; Calcd. for $C_{49}H_{44}IrN_6$, 909.1. Elemental Analysis (%): calcd. for $C_{50}H_{44}F_3ClIrN_6O_3S{\cdot}(CH_2Cl_2)_{0.5}$: C, 55.11; H, 4.12; N, 7.64; found: C, 54.49, H, 3.98; N, 8.06.

Complex 4^{Me}b: Overall yield: 58%.

4^{Me}b with anion of PF$_6^-$:

Yield: 17 %. δ_H (400 MHz, DMSO) = 8.39 (2 H, d, *J* 8.1 Hz), 8.28 (2 H, d, *J* 5.3 Hz), 8.09 – 8.01 (4 H, m,), 7.55 (2 H, s), 7.44 – 7.38 (2 H, m), 7.31 – 7.23 (4 H, m), 7.22 – 7.14 (4 H, m), 7.11 (2 H, d, *J* 4.8 Hz), 6.57 (2 H, d, *J* 6.4 Hz), 6.09 (2 H, s), 3.22 (4 H, d, *J* 11.4 Hz), 1.37 (6 H, s), 1.33 (6 H, s), 1.07 (2 H, t, *J* 7.0 Hz), 0.98 – 0.86 (2 H, m), 0.56 – 0.44 (4 H, m), 0.31 – 0.22 (8 H, m). δ_F (376 MHz, CD_3CN) = -70.10 (d, *J* 711.2 Hz) ppm. δ_P (162 MHz, CD_3CN) = -144.09 (sept, *J* 711.2 Hz) ppm.; Elemental Analysis (%): calcd. for $C_{55}H_{56}F_6IrN_6P{\cdot}(H_2O)_2$: C, 56.25; H, 5.15; N, 7.16; found: C, 56.06, H, 5.00; N, 7.16.

4^{Me}b with anion of OTf$^-$:

Yield: 41 %. δ_H (400 MHz, CD_3CN) = 8.30 (2 H, d, *J* 5.7 Hz), 8.22 (2 H, d, *J* 8.1 Hz,), 7.97 (2 H, t, *J* 7.8 Hz), 7.93 (2H, s), 7.40 (2 H, d, *J* 7.4 Hz), 7.33 (2 H, d, *J* 1.9 Hz), 7.23 (6 H, ddd, *J* 13.9, 8.2, 7.1 Hz), 7.15 (2 H, t, *J* 6.6 Hz,), 7.01 (2 H, d, *J* 1.9 Hz), 6.72 (2 H, s), 5.97 (2 H, s), 3.48 (2 H, td, *J* 12.5, 5.3 Hz), 3.24 (2 H, td, *J* 12.5, 5.3 Hz), 1.43 (6 H, s), 1.33 (6 H, s), 1.02 – 0.90 (2 H, m), 0.54 (4 H, dt, *J* 13.4, 6.4 Hz), 0.47 – 0.36 (2H, m), 0.32 ppm (6 H, t, *J* 7.2 Hz). δ_F (376 MHz, CD_3CN) = -79.29 ppm. ESI-MS: *m/z* 993.5 [*M-OTf*]$^+$; Calcd. for $C_{55}H_{56}IrN_6$, 993.4. Elemental Analysis (%): calcd. for $C_{56}H_{56}F_3IrN_6SO_3$: C, 58.88; H, 4.94; N, 7.36; found: C, 58.38, H, 4.83; N, 7.33.

Complex 4^{Me}c:

Yield: δ_H (400 MHz, CD_3OD) 8.41 – 8.26 (4 H, m), 8.02 (2 H, s), 7.94 (2 H, s), 7.41 (2 H, s), 7.35 (4 H, s), 7.23 – 7.14 (8 H, m), 6.65 (2 H, d, *J* 1.8), 6.16 (2 H, d, *J* 3.1), 5.19 (2 H, s), 3.97 – 3.89 (2 H, m), 3.81 – 3.68 (4 H, m), 3.66 – 3.56 (4 H, m), 3.54 (2 H, s), 3.25 – 3.11 (6 H, m), 3.06 (1 H, ddd, *J* 6.5, 4.3, 2.7), 3.02 – 2.86 (4 H, m), 2.11 – 2.01 (1 H, m), 1.60 – 1.51 (2 H, m), 1.46 (6 H, s), 1.39 (6 H, s), 1.28 (2 H, s). δ_F (376 MHz, CD_3CN) = -79.29 ppm. ESI-MS: *m/z* 1293.9 [*M-OTf*]$^+$; Calcd. for $C_{63}H_{68}IrN_6O_{12}$, 1293.5.

Complex 5b:

Yield: 80%. δ_H (400 MHz, CD_3OD) = 8.33 (2 H, d, *J* 5.3 Hz), 8.16 (2 H, d, *J* 8.0 Hz), 7.98 (2 H, t, *J* 7.7 Hz), 7.86 (2 H, s), 7.79 – 7.73 (4 H, m), 7.69 – 7.62 (6 H, m), 7.59 (2 H, d, *J* 1.6 Hz), 7.49 (2 H, dd, *J* 8.1, 1.7 Hz), 7.22 (2 H, s), 7.16 (2 H, t, *J* 6.1 Hz), 6.90 (2 H, d, *J* 1.8 Hz), 6.84 (2 H, s), 6.26 (2 H, s), 3.64 – 3.51 (2 H, m), 3.33 – 3.21 (2 H, m), 2.09 – 1.96 (8 H, m), 1.56 (9 H, s), 1.53 (6 H, s), 1.49 (6 H, s), 1.14 – 0.91 (27 H, m), 0.81 – 0.72 (12 H, m), 0.70 – 0.44 (16 H, m), 0.36 ppm (6 H, t, *J* 7.2 Hz). δ_F (376 MHz, CD_3OD) = -78.91 ppm. ESI-MS: *m/z* 1896.5 [*M-OTf*]$^+$; Calcd. for $C_{111}H_{126}Br_2IrN_6$, 1896.8. Elemental Analysis (%): calcd. for $C_{112}H_{126}Br_2F_3IrN_6O_3S$: C, 65.77; H, 6.21; N, 4.11; found: C, 65.45, H, 6.13; N, 4.12.

Complex 6^{H}b:

Yield: 95%. δ_H (400 MHz, CD_3CN) = 8.11 (2 H, d, *J* 8.0 Hz), 7.81 (2H, d, *J* 7.6 Hz), 7.49 (2 H, t, *J* 7.5 Hz), 7.37 (2 H, s), 7.27 (2 H, t, *J* 7.7 Hz), 7.05 (2 H, s), 6.95 (2 H, t, *J* 7.4 Hz), 6.77 (2 H, t, *J* 7.4 Hz), 6.50 (2 H, d, *J* 7.5 Hz), 6.21 (2 H, d, *J* 8.3 Hz), 5.65 (2 H, s), 3.18 (2 H, td, *J* 12.4, 5.3 Hz), 2.95 (2 H, td, *J* 12.3, 4.2 Hz), 1.01 – 0.85 (2 H, m), 0.71 – 0.55 (4 H, m), 0.49 (6 H, t, *J* 7.0 Hz), 0.39 – 0.22 ppm (2 H, m). δ_F (376 MHz, CD_3CN) = -79.30 ppm. δ_C (101 MHz, CD_3CN) = 181.63, 164.36, 159.47, 150.38, 141.22, 132.85, 131.29, 130.32, 127.64, 126.03, 123.76, 122.04, 122.01, 121.67, 118.31, 63.09, 49.16, 32.31, 19.21, 12.59 ppm. ESI-MS: *m/z* 873.5 [*M-OTf*]$^+$; Calcd. for $C_{41}H_{40}IrN_6S_2$, 873.2. Elemental Analysis (%): calcd. for $C_{42}H_{40}F_3IrN_6O_3S_3{\cdot}H_2O$: C, 48.49; H, 4.07; N, 8.08; found: C, 48.79, H, 4.08; N, 7.97.

Complex cis-6^{H}b:

Yield: 94%. To a 4 mL acetonitrile solution in a test tube was added 100mg of **6^{H}b**, the resulting mixture was degassed by nitrogen gas for 10mins. The solution was irradiated by blue LEDs (12 w) for 12 hr. After removing solvents, the mixture was purified by chromatographic column, and eluted by DCM/MeCN (V/V = 20/3). After removed organic solvent, the yellowish solid was in good yield (94%) for complex of **cis-6^{H}b**: δ_H (400 MHz, CD_3CN) 8.19 (1 H, d, *J* 7.8 Hz), 7.95 (1 H, d, *J* 7.7 Hz), 7.88 (1 H, d, *J* 8.0 Hz), 7.66 (1 H, d, *J* 7.4 Hz), 7.57 (1 H, t, *J* 7.7 Hz), 7.42 (1 H, t, *J* 7.8 Hz), 7.39 (1 H, d, *J* 1.9 Hz), 7.25– 7.18 (3 H, m), 7.13 (1 H, d, *J* 2.0 Hz), 7.05 (1 H, t, *J* 6.9 Hz), 6.99 (1 H, d, *J* 2.1 Hz), 6.90 (1 H, t, *J* 7.4 Hz), 6.85 (2 H, t, *J* 7.5 Hz), 6.79 (2 H, dd, *J* 7.4, 2.8 Hz), 6.63 (1 H, d, *J* 8.3 Hz), 6.36 (1 H, d, *J* 8.4 Hz), 5.99 (1 H, d, *J* 13.5 Hz), 5.72 (1 H, d, *J* 13.5 Hz), 3.47 – 3.33 (2 H, m), 3.27 – 3.18 (1 H, m), 3.17 – 3.00 (2 H, m), 1.13 (1 H, t, *J* 7.0 Hz), 1.02 – 0.84 (2 H, m), 0.77 – 0.62 (2 H, m), 0.55 (3 H, t, *J* 7.2 Hz), 0.51– 0.40 (1 H, m), 0.36 – 0.07 (6 H, m). δ_C (101 MHz, CD_3CN) 178.74, 175.67, 162.70, 160.36, 152.06, 150.18, 149.64, 142.70, 140.41, 140.25, 137.17, 134.54, 132.30, 131.46, 131.42, 130.91, 128.09, 127.02, 126.98, 126.24, 125.90, 125.35, 123.85, 123.60, 123.07, 122.15, 122.04, 121.92, 121.87, 121.41, 119.18, 118.47, 62.48, 49.92, 48.64, 33.25, 32.47, 19.28, 18.92, 12.67, 12.51. δ_F (376 MHz, CD_3CN) = -79.31 ppm. ESI-MS: *m/z* 873.5 [*M-OTf*]$^+$; Calcd. for $C_{41}H_{40}IrN_6S_2$, 873.2. Elemental Analysis (%): calcd. for $C_{42}H_{40}F_3IrN_6O_3S_3{\cdot}(H_2O)_{0.5}$: C, 48.92; H, 4.01; N, 8.15; found: C, 49.15, H, 4.00; N, 8.04.

Complex 6^{H}a, with anion of OTf$^-$:

Yield of 58%. δ_H (400 MHz, CD_3CN) 8.10 (2 H, d, *J* 8.1Hz), 7.82 (2 H, d, *J* 7.7 Hz), 7.49 (2 H, t, *J* 7.7 Hz), 7.32 (2 H, d, *J* 1.5 Hz), 7.28 (2 H, t, *J* 7.8 Hz), 6.99 – 6.91 (4 H, m), 6.76 (2 H, t, *J* 7.4 Hz), 6.54 (2 H, d, *J* 7.6 Hz), 6.30 (2 H, d, *J* 8.4 Hz), 5.68 (2 H, s), 2.62 (6 H, s) ppm. δ_F (376 MHz, CD_3CN) -79.32 ppm. ESI-MS: *m/z* 789.2 [*M-OTf*]$^+$; Calcd. for $C_{35}H_{28}IrN_6S_2$, 789.0. Elemental Analysis (%): calcd. for $C_{36}H_{28}F_3IrN_6O_3S_3{\cdot}H_2O$: C, 45.23; H, 3.16; N, 8.79; found: C, 45.61, H, 3.19; N, 8.65.

Complex 6^{H}c:

Typical procedure for synthesis of Ir(III) complexes bearing D-glucose on terminal of bis-NHC carbene ligand (**[(C^N)$_2$Ir(dig)]OTf**). To a two-neck flask were added 1,1'-methylenebis(3-(2-((3,4,5-triacetoxy-6-(acetoxymethyl)tetrahydro-2H-pyran-2-yl)oxy)ethyl)-1H-imidazol-3-ium), 2Br$^-$ (392 mg, 0.37 mmol, 2.2 eq), $[(hbta)_2IrCl]_2$ (218 mg, 0.168 mmol, 1 eq.), and silver(I) oxide (171 mg, 0.74 mmol, 4.4 eq) in 2-methoxyethanol (volume: 10 mL) to give a black suspension. The reaction was refluxing at 120 °C for overnight, 2-methoxyethanol (10 mL) was removed under vacuum pump. 40 mL of MeOH was added and filtrated through celite to remove unreacted silver oxide, the resulting filtrate was concentrated, and 5 mL of MeOH was added, 30 mL of Et_2O was added to result precipitate. The crude product was prepared to purify by chromatography, by using the eluent system of MeCN(mL)/MeOH(mL)/LiOTf(mg)(100/60/320). After organic solvent is removed, the yellowish green solid was in good yield of 83% complex of **6^{H}c**: 367 mg, 0.278 mmol). δ_H (400 MHz, CD_3OD) = 8.13 (2 H, d, *J* 7.7 Hz), 7.82 (2 H, d, *J* 7.4 Hz), 7.55 – 7.41 (6 H, m), 7.32 (2 H, dd, *J* 15.0, 7.4 Hz), 6.96 (2 H, t, *J* 7.2 Hz), 6.78 (2 H, t, *J* 7.2 Hz), 6.44 (2 H, d, *J* 7.4 Hz), 6.23 (2 H, t, *J* 7.7 Hz), 5.82 (2 H, s), 4.34 – 4.13 (1H, m), 3.91 (1H, d, *J* 7.8 Hz), 3.80 – 3.45 (9 H, m), 3.42 – 3.35 (2 H, m), 3.27 – 3.08 (7 H, m), 3.06 – 2.95 (3 H, m), 2.94 – 2.81 (1 H, m), 2.56 – 2.46 (1 H, t), 2.04 – 1.87 (3 H, m) ppm. δ_F (376 MHz, CD_3OD) = -80.03 ppm. ESI-MS: *m/z* 1173.5 [*M-OTf*]$^+$; Calcd. for $C_{49}H_{52}IrN_6O_{12}S_2$, 1173.3. Elemental Analysis (%): calcd. for $C_{50}H_{52}F_3IrN_6O_{15}S_3{\cdot}(LiSO_3CF_3)_{1.2}{\cdot}(H_2O)_8$: C, 37.19; H, 4.14; N, 5.08; found: C, 36.88, H, 4.17; N, 5.09.

Complex 6^{F}b:

Yield: 78%. δ_H (400 MHz, CD_3CN) δ = 8.49 (2 H, s), 7.89 (2 H, d, *J* 7.6 Hz), 7.56 (2 H, d, *J* 8.8 Hz), 7.39 (2 H, s), 7.07 (2 H, s), 6.99 (2 H, t, *J* 7.4 Hz), 6.81 (2 H, t, *J* 7.3 Hz), 6.51 (2 H, d, *J* 7.5 Hz), 6.30 (2 H, d, *J* 8.7 Hz), 5.63 (2 H, s), 3.16 (2 H, td, *J* 12.4, 5.5 Hz), 2.93 (2 H, td, *J* 12.4, 4.6 Hz), 1.04 – 0.81 (2 H, m), 0.74 – 0.53 (4 H, m), 0.50 (6 H, t, *J* = 6.9 Hz), 0.40 – 0.21 (2H, m) ppm. δ_F (376 MHz, CD_3CN) = -62.16, -79.33 ppm. ESI-MS: *m/z* 1009.5 [*M-OTf*]$^+$; Calcd. for $C_{43}H_{38}F_6IrN_6S_2$, 1009.2. Elemental Analysis (%): calcd. for $C_{44}H_{40}F_9IrN_6O_3S_3{\cdot}H_2O$: C, 44.93; H, 3.43; N, 7.14; found: C, 45.17, H, 3.32; N, 7.01.

Complex 7b:

Yield: 24%. ^{1}H NMR (500 MHz, CD_3CN, 238 K) δ = 8.02 (1 H, d, *J* 8.3 Hz), 7.96 (1 H, d, *J* 8.0 Hz), 7.88 (1 H, s), 7.76 (1 H, d, *J* 8.7 Hz), 7.53 (1 H, t, *J* 7.5 Hz), 7.46 (1 H, s), 7.37 – 7.29 (4 H, m), 7.22 (1 H, d, *J* 1.7 Hz), 7.17 (1 H, d, *J* 1.7 Hz), 7.01 (1 H, d, *J* = 1.7 Hz), 6.89 – 6.82 (2 H, m), 6.22 (2H, dd, *J* 12.9, 4.7 Hz), 5.70 (1 H, d, *J* 8.9 Hz), 5.57 (1 H, d, *J* 13.6 Hz), 4.62 (1 H, d, *J* 13.4 Hz), 3.16 – 3.02 (2 H, m), 2.88 (3 H, s), 2.75 (3 H, s), 2.21 (1 H, t, *J* 10.9 Hz), 1.10 – 1.01 (1 H, m), 0.88 – 0.79 (1 H, m), 0.71 (1 H,

t, *J* 7.2 Hz), 0.66 – 0.41 (8 H, m), 0.36 (3 H, t, *J* 7.1 Hz), 0.26 – 0.14 (1 H, m), -0.26 (1 H, s). δ_F (376 MHz, CD_3CN, 300 K) = -78.27 ppm. ESI-MS: *m/z* 901.4 [*M-OTf*]$^+$; Calcd. for $C_{43}H_{44}IrN_6S_2$, 901.3. Elemental Analysis (%): calcd. for $C_{44}H_{44}F_3IrN_6O_3S_3{\cdot}H_2O$: C, 49.47; H, 4.34; N, 7.87; found: C, 49.34, H, 4.12; N, 7.90.

Complex 7c:

Yield: 58%. δ_H (500 MHz, MeOH, 228 K) 8.12 (1 H, d, *J* 7.1 Hz), 8.07 (1 H, t, *J* 7.9 Hz), 7.97 (1 H, s), 7.84 (1 H, t, *J* 9.8 Hz), 7.62 – 7.57 (1 H, m, *J* 7.4 Hz), 7.56 – 7.27 (10 H, m), 6.99 – 6.89 (1 H, m), 6.32 – 6.22 (1 H, m), 6.15 – 6.06 (1 H, m), 5.99 (1 H, d, *J* 12.0 Hz), 5.80 (1 H, d, *J* 8.5 Hz), 4.74 (1 H, s), 4.08 – 3.90 (1 H, m), 3.79 (1 H, d, *J* 7.8 Hz), 3.74 – 3.46 (6 H, m), 3.24 – 3.12 (5 H, m), 3.09 – 3.03 (2 H, m), 2.96 (3 H, s), 2.82 (3 H, s), 2.74 – 2.57 (2 H, m), 2.53 – 2.29 (1 H, m), 2.06 – 1.74 (1 H, m). ESI-MS: *m/z* 1201.5 [*M-OTf*]$^+$; Calcd. for $C_{51}H_{56}IrN_6O_{12}S_2$, 1201.4. Elemental Analysis (%): calcd. for $C_{52}H_{56}F_3IrN_6O_{15}S_3[(LiSO_3CF_3)_{0.6}(H_2O)_6]$: C, 40.70; H, 4.42; N, 5.41; found: C, 41.19, H, 4.38; N, 5.30.

Complex 8b:

Yield: 62%. δ_H (400 MHz, CD_3CN) = 9.20 (2 H, d, *J* 4.8 Hz), 8.85 (2 H, s), 8.15 (4 H, t, *J* 8.0 Hz), 7.94 (4 H, d, *J* 7.7 Hz), 7.79 (2 H, d, *J* 7.6 Hz), 7.56 (2 H, d, *J* 6.4 Hz), 7.30 (s, 2H), 7.18 (4 H, dd, *J* 14.5, 7.1 Hz), 7.11 (2 H, d, *J* 7.6 Hz), 7.01 (4 H, d, *J* 5.4 Hz), 5.80 (2 H, s), 3.46 – 3.37 (2 H, m), 3.14 (2 H, td, *J* 12.6, 4.8 Hz), 1.01 – 0.82 (2 H, m), 0.40 – 0.26 (2 H, m), 0.25 – 0.10 (4 H, m), -0.03 ppm (6 H, t, *J* 7.0 Hz). δ_F (376 MHz, CD_3CN) = -79.34 ppm. ESI-MS: *m/z* 961.7 [*M-OTf*]$^+$; Calcd. for $C_{53}H_{48}IrN_6$, 961.4. Elemental Analysis (%): calcd. for $C_{54}H_{48}F_3IrN_6O_3S{\cdot}H_2O$: C, 57.48; H, 4.47; N, 7.45; found: C, 56.91, H, 4.31; N, 7.26.

Complex 9b:

Yield: 53%. δ_H (400 MHz, CD_3CN) = 8.83 (2 H, d, *J* 5.7 Hz), 8.00 (4 H, d, *J* 6.2 Hz), 7.82 (4 H, dd, *J* 6.0, 3.1 Hz), 7.44 (2 H, d, *J* 4.7 Hz), 7.35 (2 H, s), 7.29 (2 H, d, *J* 6.6 Hz), 7.06 (2 H, s), 6.28 (2 H, d, *J* 4.8 Hz), 5.93 (2 H, s), 3.31 (4 H, pd, *J* = 13.0, 5.3 Hz), 1.11 – 0.99 (2 H, m), 0.82 – 0.68 (2 H, m), 0.61 (2 H, ddd, *J* 17.4, 11.5, 6.1 Hz), 0.39 (6 H, t, *J* 7.3 Hz), 0.26 – 0.09 (2 H, m) ppm. δ_F (376 MHz, CD_3CN) = -79.32 ppm. ESI-MS: *m/z* 873.6 [*M-OTf*]$^+$; Calcd. for $C_{41}H_{40}IrN_6S_2$, 873.2. Elemental Analysis (%): calcd. for $C_{42}H_{40}F_3IrN_6O_3S_3{\cdot}(H_2O)_{0.5}$: C, 48.92; H, 4.01; N, 8.15; found: C, 48.80, H, 4.01; N, 8.12.

Complex 9c:

Yield of 54%, δ_H (400 MHz, CD_3OD) δ = 8.86 (2 H, d, *J* 4.3 Hz), 8.07 – 7.93 (4 H, m), 7.86 – 7.77 (4 H, m), 7.51 – 7.37 (6 H, m), 7.38 (2 H, d, *J* 4.0 Hz), 6.27 – 6.17 (2 H, m), 6.12 (2 H, d, *J* 2.7 Hz), 3.96 – 3.80 (3 H, m), 3.72 (7 H, t, *J* 6.5 Hz), 3.64 (3 H, s), 3.62 – 3.45 (4 H, m), 3.34 (1 H, s), 3.27 – 3.11 (6 H, m), 3.08 – 2.98 (3 H, m), 2.96 – 2.89 (1 H, m), 2.80 – 2.70 (1 H, m), 2.30 – 2.21 (1 H, m) ppm. δ_F (376 MHz, CD_3OD) δ = -80.09 ppm. ESI-MS: *m/z* 1173.5 [*M-OTf*]$^+$; Calcd. for $C_{49}H_{52}IrN_6O_{12}S_2$, 1173.3. Elemental Analysis (%): calcd. for $C_{50}H_{52}F_3IrN_6O_{15}S_3{\cdot}(LiSO_3CF_3)_{0.5}{\cdot}(H_2O)_4$: C, 41.19; H, 4.11; N, 5.71; found: C, 41.99, H, 4.10; N, 5.55.

Synthesis of C^N ligands:

Ligand of HL3:

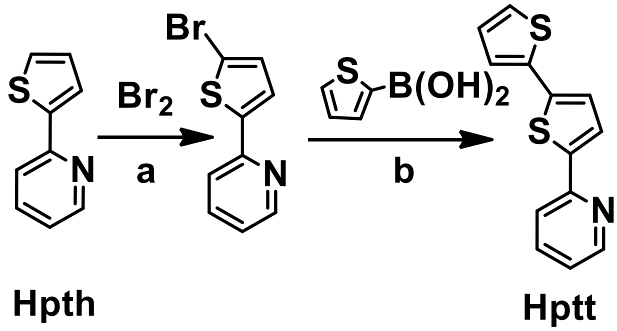

Scheme 1, a) i, DCM (0°C to r.t.) b) K_2CO_3, [Pd(dppf)]Cl_2, Toluene/EtOH/H_2O (V/V/V = 5/5/2), reflux.

Synthesis of 2-(5-bromothiophen-2-yl)pyridine:

To a 100 mL round-bottomed flask was added 2-(thiophen-2-yl)pyridine (700 mg, 4.34mmol) in DCM to give a yellow solution. Bromine (694 mg, 4.34mmol) in 5 mL of DCM was added at ice bath. After addition for 5mins, red precipitate was formed, and it was left stirring for overnight. 75% was converted after HNMR identification. Another 150 mg of bromine was added again. The crude material was loaded on a 2.0mm plate. The plate was eluted by DCM/Hexane (V/V = 1/2), Collect the fraction of R_f = 0.3. The product was collected with the yield of 100% (1.04g, 4.33 mmol). ^{1}H NMR (300 MHz, $CDCl_3$) δ = 8.54 (d, J = 4.9 Hz, 1H), 7.69 (td, J = 7.5, 1.4 Hz, 1H), 7.58 (d, J = 8.0 Hz, 1H), 7.31 (d, J = 3.9 Hz, 1H), 7.16 (dd, J = 7.3, 5.0 Hz, 1H), 7.07 ppm (d, J = 3.9 Hz, 1H).

Synthesis of 2-[(2,2'-bithiophen)-5-yl]pyridine (Hptt: L3):

2-(5-bromothiophen-2-yl)pyridine was used without further purification. To a two-neck flask were added potassium carbonate (1.796g, 12.99 mmol, 3 eq.), 2-(5-bromothiophen-2-yl)pyridine (1.04 g, 4.33 mmol, 1 eq.), thiophen-2-ylboronic acid (665 mg, 5.2 mmol, 1.2 eq.) and [Pd(dppf)]Cl_2 (317 mg, 0.433 mmol, 0.1 eq.) into an aqueous solution of toluene/ethanol/water (V/V/V = 25 mL/25 mL/10 mL) to give a solution. The mixture was degassed by nitrogen for 20mins, and then left to reflux at 100 °C for overnight. The reaction was monitored by TLC, and the mixture was extracted by DCM (30 mL × 2). And the organic layer was washed by water (30 mL × 2), dried by $MgSO_4$. After remove organic solvent, the crude product was prepared to purify by chromatography, by using the eluent system of DCM/Hexane (V/V from 0/1 to 1/3). 480mg of **L3** (yield of 45.5%) was collected. ^{1}H NMR (300 MHz, $CDCl_3$) δ = 8.59 – 8.53 (m, 1H), 7.73 – 7.62 (m, 2H), 7.48 (d, J = 3.9 Hz, 1H), 7.24 (d, J = 1.1 Hz, 1H), 7.19 (d, J = 3.9 Hz, 1H), 7.18 – 7.12 (m, 1H), 7.05 (dd, J = 5.1, 3.6 Hz, 1H).

Ligand of HL4^{Hex}:

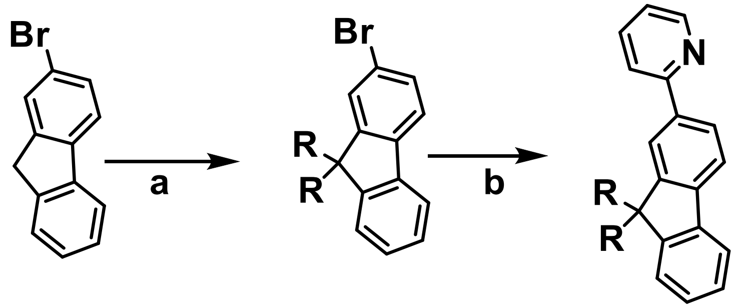

Scheme 2, a) i, KOtBu, DMF; ii, 1-Br^nC_6H_{13} for **L4^{Hex}**, MeI for **L4^{Me}**; b) i, Mg, I_2, THF, reflux (to prepare Grignard reagent); ii) above Grignard reagent, Ni(dppe)Cl_2, 2-bromopyridine, THF, reflux.

Synthesis of 2-9,9-dihexyl-9H-fluoren-2-yl)pyridine (L4):

In a 100 mL two-neck round-bottomed flask was **2-bromo-9,9-dihexyl-9H-fluorene** (0.98 g, 2.37 mmol) and magnesium (58 mg, 2.37 mmol) in dried THF (20 mL) to give a colorless suspension. Iodine 10 mg was added to initialize the reaction and refluxing for

30 mins. In a 250 mL two-neck round-bottomed flask was 2-bromopyridine (1.63 g, 10.00 mmol) and **Ni(dppe)Cl$_2$** (13 mg, 0.024 mmol) in dried THF (30.0 mL) to give a orange solution. The Grignard reagent of first solution was transferred by cannula at refluxing temperature, the resulting dark brown solution in the 250 mL flask was left stirring for 20 mins and refluxing for overnight. Monitoring of reaction shows product left without starting materials. H_2O was added to end the reaction. The aqueous layer was back extracted with Et_2O (50 mL×3). Combined the organic layers and washed with water (50 mL×2). The organic layer was dried by $MgSO_4$, filtrated and concentrated. The crude product was added to a silica gel column and was eluted with DCM/Hexane (V/V = 1/1). Collect fractions with R_f = 0.1 in DCM/Hex (V/V =1/4) with strong blue emission under UV lamp (285 nm). Purified **L4^{Hex}**: 0.68g, Yield: 70%. ^{1}H NMR (400 MHz, $CDCl_3$) δ = 8.73 (d, J = 4.5, 1H), 8.03 – 7.93 (m, 2H), 7.83 – 7.69 (m, 4H), 7.35 (t, J=6.0, 3H), 7.25 – 7.17 (m, 2H), 2.11 – 1.94 (m, 5H), 1.14 – 0.97 (m, 14H), 0.74 (t, J = 6.9, 7H), 0.67 – 0.56 (m, 4H). Purified **L4^{Me}**: 4.5 g, 16.6 mmol from **2-bromo-9,9-dimethyl-9H-fluorene** (5.1g, 18.7 mmol), yield: 89%. ^{1}H NMR (400 MHz, $CDCl_3$) δ = 8.76 – 8.69 (m, 1H), 8.14 (s, 1H), 7.96 (dd, J = 7.9, 1.7 Hz, 1H), 7.84 – 7.74 (m, 4H), 7.49 – 7.43 (m, 1H), 7.38 – 7.31 (m, 2H), 7.28 – 7.23 (m, 1H), 1.56 (s, 6H).

Ligand of HL5:

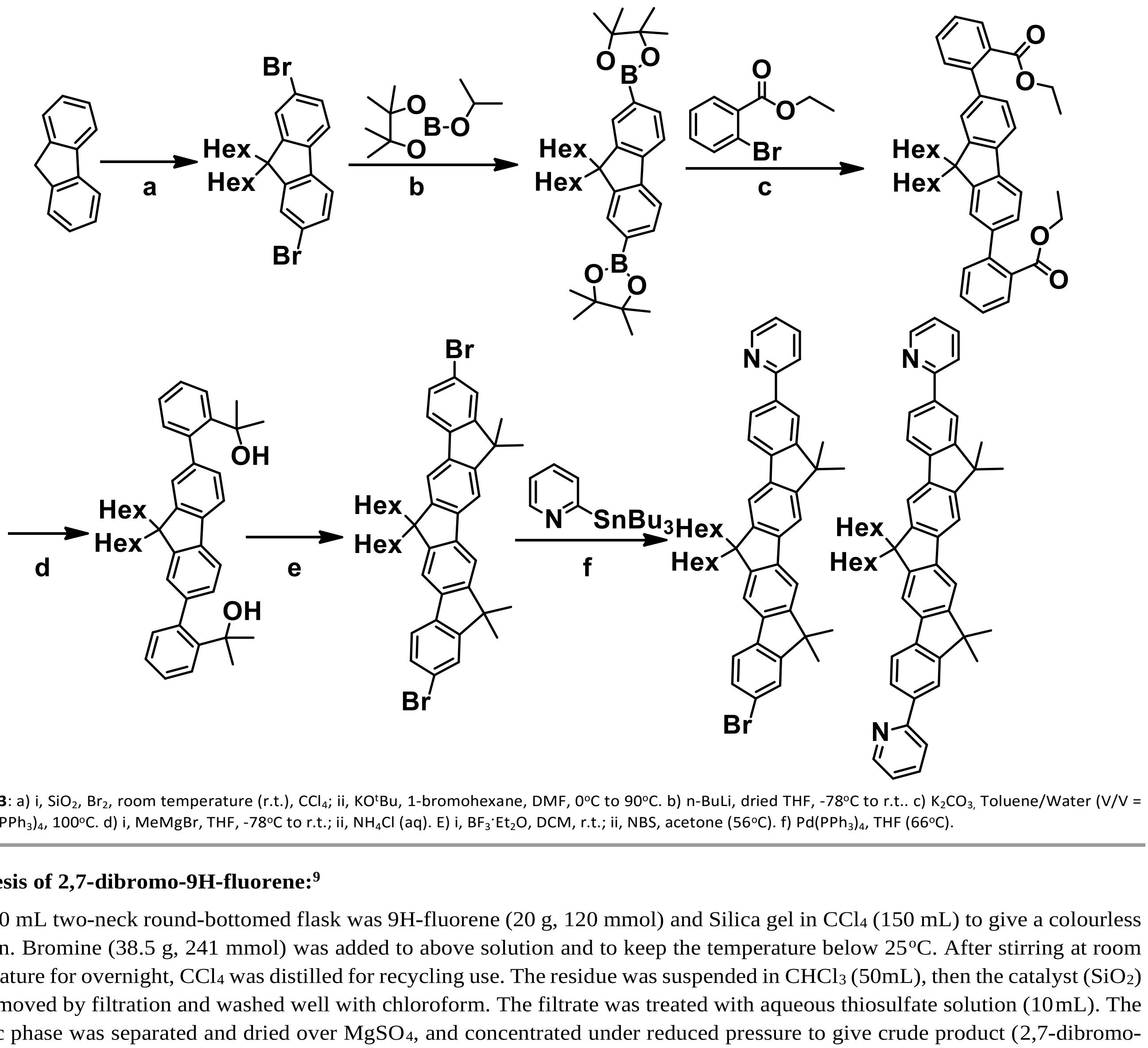

Scheme 3: a) i, SiO_2, Br_2, room temperature (r.t.), CCl_4; ii, KO^{t}Bu, 1-bromohexane, DMF, 0°C to 90°C. b) n-BuLi, dried THF, -78°C to r.t.. c) K_2CO_3, Toluene/Water (V/V = 4/1), $Pd(PPh_3)_4$, 100°C. d) i, MeMgBr, THF, -78°C to r.t.; ii, NH_4Cl (aq). E) i, $BF_3 \cdot Et_2O$, DCM, r.t.; ii, NBS, acetone (56°C). f) $Pd(PPh_3)_4$, THF (66°C).

Synthesis of 2,7-dibromo-9H-fluorene:[9]

In a 100 mL two-neck round-bottomed flask was 9H-fluorene (20 g, 120 mmol) and Silica gel in CCl_4 (150 mL) to give a colourless solution. Bromine (38.5 g, 241 mmol) was added to above solution and to keep the temperature below 25°C. After stirring at room temperature for overnight, CCl_4 was distilled for recycling use. The residue was suspended in $CHCl_3$ (50mL), then the catalyst (SiO_2) was removed by filtration and washed well with chloroform. The filtrate was treated with aqueous thiosulfate solution (10mL). The organic phase was separated and dried over $MgSO_4$, and concentrated under reduced pressure to give crude product (2,7-dibromo-9H-fluorene (36.5 g, 113 mmol, 94 % yield)). ^{1}H NMR (400 MHz, $CDCl_3$) δ = 7.66 (s, 2H), 7.59 (d, J = 8.1 Hz, 2H), 7.50 (dd, J = 8.1, 1.3 Hz, 2H), 3.86 ppm (s, 2H).

Synthesis of 2,7-dibromo-9,9-dihexyl-9H-fluorene:

To a two-neck round-bottomed flask was added 2,7-dibromo-9H-fluorene (20g, 61.7 mmol, 1eq.) into DMF (150 mL) to give clear solution. Potassium t-butoxide (15.24g, 136 mmol, 2.2 eq.) was added into above solution. After stirring for 20mins, 1-bromohexane was divided into 3 lots and added into reaction under ice bath (caution: exothermic reaction). The mixture was heated to reflux for overnight until the reaction bottle was cooled down. DMF was evaporated after the reaction finished. DCM (200mL) was added into residue, resulting organic solution was washed by water (50 mL×2) and brine (20mL×2), dried over $MgSO_4$. The crude compound was purified by chromatograph, eluted by Hexane to get desired compound (25g, 50.8 mmol, yield: 82%). ^{1}H NMR (400 MHz, $CDCl_3$) δ = 7.54 – 7.49 (m, 2H), 7.47 – 7.41 (m, 4H), 1.95 – 1.87 (m, 4H), 1.17 – 1.08 (m, 4H), 1.07 – 0.99 (m, 8H), 0.77 (dd, J=8.9, 5.3 Hz, 6H), 0.56 ppm (dd, J=15.8, 7.2 Hz, 4H).

Synthesis of 2,2'-(9,9-dihexyl-9H-fluorene-2,7-diyl)bis(4,4,5,5-tetramethyl-1,3,2-dioxaborolane):

In a 250 mL two-neck round-bottomed flask was added 2,7-dibromo-9,9-dihexyl-9H-fluorene (5 g, 10.16 mmol) in THF (100 mL) to give a colorless solution. n-Butyllithium (10.16 mL, 24.37 mmol, 2.4M) was added by syringe into the flask which was placed in acetone/Liquid nitrogen bath. And the resulting white solid was left to warm up till the suspension could be stirring and the bottle was placed into acetone bath again, 2-isopropoxy-4,4,5,5-tetramethyl-1,3,2-dioxaborolane (4.53 g, 24.37 mmol) was added into the flask. The white solid was disappearing and the reaction was left to warm up to room temperature for overnight. Saturated $NaHCO_3$ solution was added to quench the reaction. THF was removed and resulting mixture was extracted with Et_2O (3 × 50 mL). Combined the organic layers and wash with water (2 × 20 mL). The organic was dried over $MgSO_4$, filtrated and concentrated. The mixture was prepared for chromatograph by silica gel and eluted by DCM/Hex (V/V from 1/25 R_f : 0.05 to 1/1 R_f : 0.2) then by EA/Hex (1/10 R_f = 0.4). 2,2'-(9,9-dihexyl-9H-fluorene-2,7-diyl)bis(4,4,5,5-tetramethyl-1,3,2-dioxaborolane) (1.82 g, 3.10 mmol, 30.6 % yield) was synthesized. ^{1}H NMR (400 MHz, $CDCl_3$) δ = 7.80 (d, J = 7.6 Hz, 2H), 7.75 – 7.69 (m, 4H), 2.04 – 1.95 (m, 4H), 1.38 (s, 24H), 1.10 – 0.96 (m, 12H), 0.74 (t, J = 7.1 Hz, 6H), 0.54 ppm (d, J = 6.7 Hz, 4H).

Synthesis of diethyl 2,2'-(9,9-dihexyl-9H-fluorene-2,7-diyl)dibenzoate[10]:

To a 100 mL two-neck round-bottomed flask were added ethyl 2-bromobenzoate (1.547 g, 6.75 mmol), 2,2'-(9,9-dihexyl-9H-fluorene-2,7-diyl)bis(4,4,5,5-tetramethyl-1,3,2-dioxaborolane) (1.8 g, and K_2CO_3 (2.121 g, 15.35 mmol) 3.07 mmol), in Toluene (40 mL) to give a colorless solution. Water (10 mL) and $Pd(PPh_3)_4$ (0.177 g, 0.153 mmol) were added and the mixture was degassed by nitrogen gas for 15 mins. The reaction was left to reflux for overnight. After monitored by thin layer chromatograph plate, the reaction was quenched by adding water. The mixture was extracted by Et_2O (3×50 mL). And the combined organic layers was washed by water (2×30 mL), dried over $MgSO_4$, filtrated and concentrated. The residue was prepared to purify by chromatograph and pre-eluted by DCM/Hex (V/V from 1/25 to 1/1) to remove the first byproduct, and then eluted by using Hex/DCM/EA (V/V/V = 2/1/0.3) to get the product (1.69g, 2.68 mmol, yield: 87%). ^{1}H NMR (400 MHz, $CDCl_3$) δ = 7.82 (d, J = 7.7 Hz, 2H), 7.72 (d, J = 7.6 Hz, 2H), 7.55 (t, J = 4.4 Hz, 2H), 7.46 – 7.40 (m, 4H), 7.32 – 7.27 (m, 4H), 4.07 (q, J = 7.1 Hz, 4H), 2.02 – 1.92 (m, 4H), 1.17 – 0.95 (m, 18H), 0.80 – 0.67 (m, 10H).

Synthesis of 2,2'-((9,9-dihexyl-9H-fluorene-2,7-diyl)bis(2,1-phenylene))bis(propan-2-ol):

To a 100 mL two-neck round-bottomed flask was added diethyl 2,2'-(9,9-dihexyl-9H-fluorene-2,7-diyl)dibenzoate (1.69 g, 2.68 mmol) in dried THF (40 mL) to give a colorless solution. Methylmagnesium bromide (14 mL, 19.60 mmol) was added to the bottle at -78°C and the mixture was warmed to room temperature for overnight. The reaction was quenched by saturated NH_4Cl aqueous solution, and extracted with Et_2O (3×30 mL). The combined organic layer was washed by water (3×30 mL), dried over $MgSO_4$, filtrated and concentrated. The crude product was added to a silica gel column and was eluted with EA/Hex (V/V from 1/10 to 1/5). Collected the fractions which was the second blue spot with strong emission under UV 385nm and the R_f value of 0.5 in EA/Hex (V/V = 1/5). 2,2'-((9,9-dihexyl-9H-fluorene-2,7-diyl)bis(2,1-phenylene))bis(propan-2-ol) (550 mg, 0.912 mmol, 34.1 % yield) was purified for characterization. ^{1}H NMR (400 MHz, $CDCl_3$) δ = 7.74 (dd, J = 8.4, 1.6 Hz, 4H), 7.39 (td, J = 7.3, 1.4 Hz, 2H), 7.32 – 7.27 (m, 6H), 7.16 (dd, J = 7.5, 1.2 Hz, 2H), 1.99 – 1.92 (m, 4H), 1.84 (s, 2H), 1.47 (s, 12H), 1.16 – 1.08 (m, 4H), 1.07 – 0.99 (m, 8H), 0.77 (t, J = 7.1 Hz, 6H), 0.73 – 0.61 ppm (m, 4H).

Synthesis of 6,6-dihexyl-12,12,15,15-tetramethyl-12,15-dihydro-6H-cyclopenta[1,2-b:5,4-b']difluorene:

To the above diol compound (550 mg, 0.912 mmol, 1 eq.) in DCM (20 mL) was added 4 mL of $BF_3 \cdot Et_2O$ (4.6 g, 32.4 mmol, 35.5 eq.) dropwise under nitrogen protection and the resulting solution was stirred for 10 mins. 10mL of water was added to quenching the reaction. The mixture was extracted by Et_2O, washed by water, dried over $MgSO_4$, filtrated and concentrated. The product was used without further purification (491 mg, 0.866 mmol, yield: 95%). ^{1}H NMR (400 MHz, $CDCl_3$) δ = 7.82 – 7.75 (m, 4H), 7.65 (s, 2H), 7.46 (d, J=7.3, 2H), 7.37 (td, J = 7.3, 1.1 Hz, 2H), 7.31 (td, J = 7.3, 1.0 Hz, 2H), 2.11 – 2.01 (m, 4H), 1.58 (s, 12H), 1.15 – 0.97 (m, 12H), 0.77 – 0.66 ppm (m, 10H).

Synthesis of 2,10-dibromo-6,6-dihexyl-12,12,15,15-tetramethyl-12,15-dihydro-6H-cyclopenta[1,2-b:5,4-b']difluorene:

To a 50 mL round-bottomed flask was 6,6-dihexyl-12,12,15,15-tetramethyl-12,15-dihydro-6H-cyclopenta[1,2-b:5,4-b']difluorene (491 mg, 0.866 mmol) and 1-bromopyrrolidine-2,5-dione (NBS, 1.542 g, 8.66 mmol) in acetone (20 mL) to give a yellow suspension. The reaction was refluxing in acetone for 3h, the starting materials remained about 80%. After another 2.0 g of NBS was added, the mixture was refluxing for overnight. 578 mg of desired product was obtained (yield: 92%). ^{1}H NMR (400 MHz, $CDCl_3$) δ = 7.76 (s, 2H), 7.64 (d, J = 8.1 Hz, 2H), 7.61 (s, 2H), 7.57 (d, J = 1.6 Hz, 2H), 7.48 (dd, J = 8.1, 1.7 Hz, 2H), 2.07 – 2.01 (m, 4H), 1.56 (s, 12H), 1.12 – 0.99 (m, 12H), 0.75 – 0.66 ppm (m, 10H).

Synthesis of 2-(10-bromo-6,6-dihexyl-12,12,15,15-tetramethyl-12,15-dihydro-6H-cyclopenta[1,2-b:5,4-b']difluoren-2-yl)pyridine (L5):

In a 100 mL two-neck round-bottomed flask was 2,10-dibromo-6,6-dihexyl-12,12,15,15-tetramethyl-12,15-dihydro-6H-cyclopenta[1,2-b:5,4-b']difluorene (537 mg, 0.741 mmol), 2-(tributylstannyl)pyridine (286 mg, 0.778 mmol), and $Pd(PPh_3)_4$ (42.8 mg, 0.037 mmol) in THF (20 mL) to give a brownish orange suspension. The mixture was refluxing for overnight. Upon the reaction finished, aqueous solution of KF was added to quench the reaction. The mixture was extracted by with diethyl ether (3×25 mL). Combined the organic layers and wash with water (3×20 mL). The organic was dried over $MgSO_4$, filtrated and concentrated. The crude product was added to a silica gel column and was eluted with DCM/Hex (V/V from 1/30 to 1/3). Collect the two strongly emissive fractions. One product is **2-(10-bromo-6,6-dihexyl-12,12,15,15-tetramethyl-12,15-dihydro-6H-cyclopenta[1,2-b:5,4-b']difluoren-2-yl)pyridine** (251 mg, 0.347 mmol, 46.9 % yield). ^{1}H NMR (400 MHz, $CDCl_3$) δ = 8.74 (d, J =4.8 Hz, 1H), 8.14 (s, 1H), 8.00 (dd, J = 8.0, 1.4 Hz, 1H), 7.87 (d, J = 7.9 Hz, 1H), 7.85 – 7.75 (m, 4H), 7.69 (s, 1H), 7.66 – 7.60 (m, 2H), 7.57 (d, J = 1.6 Hz, 1H), 7.48 (dd, J = 8.0, 1.7 Hz, 1H), 7.25 – 7.21 (m, 1H), 2.10 – 2.05 (m, 4H), 1.64 (s, 6H), 1.57 (s, 6H), 1.14 – 1.00 (m, 12H), 0.78 – 0.68 ppm (m, 10H). The byproduct is **2,2'-(6,6-dihexyl-12,12,15,15-tetramethyl-12,15-dihydro-6H-cyclopenta[1,2-b:5,4-b']difluorene-2,10-diyl)dipyridine** (112mg, 0.155 mmol, yield: 21%).^{1}H NMR (400 MHz, $CDCl_3$) δ = 8.74 (d, J = 4.7 Hz, 2H), 8.15 (s, 2H), 8.00 (dd, J = 7.9 Hz, 1.4, 2H), 7.88 (d, J = 8.0 Hz, 2H), 7.85 – 7.81 (m, 4H), 7.78 (td, J = 7.7, 1.6 Hz, 2H), 7.70 (s, 2H), 7.26 – 7.21 (m, 2H), 2.12 – 2.06 (m, 4H), 1.65 (s, 12H), 1.15 – 1.02 (m, 12H), 0.80 – 0.69 (m, 10H).

Ligand of HL9:

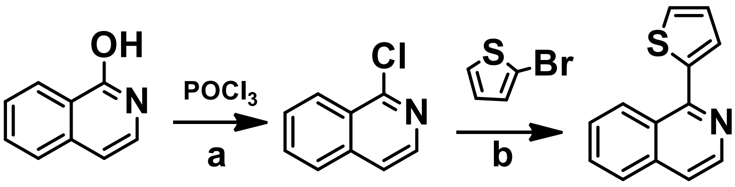

Htiq

Scheme 3, a) i, $POCl_3$ (0°C to room temperature (r.t.)); ii, ice water. b) i, Mg, I_2, 2-bromothiophene, THF, reflux (to prepare Grignard reagent); ii) above Grignard reagent, $Ni(dppe)Cl_2$, THF, reflux.

Synthesis of 1-chloroisoquinoline:

To a flask was added isoquinolin-1-ol (3g, 20.67 mmol) and phosphoryl trichloride (10 mL), and heated for 4hrs at 80°C. 100 mL of ice water was added into the cooled mixture to quench the reaction. The aqueous layer was back-extracted with DCM (30 mL× 3). Combined the organic layers and washed with water (20 mL×3). The organic layer was dried by $MgSO_4$, filtrated and concentrated. The product was used without further purification (3.12g, 92%).^{1}H NMR (400 MHz, $CDCl_3$) δ = 8.35 (d, J = 8.3 Hz, 1H), 8.28 (d, J = 5.6 Hz, 1H), 7.85 (d, J = 8.0 Hz, 1H), 7.76 (t, J = 7.5 Hz, 1H), 7.70 (t, J = 7.6 Hz, 1H), 7.61 ppm (d, J = 5.6 Hz, 1H).

Synthesis of 1-(thiophene-2-yl)isoquinoline (Htiq):

In a 100 mL two-neck round-bottomed flask was 2-bromothiophene (1.95 g, 12 mmol) and magnesium (0.291 g, 12.00 mmol) in dried THF (20 mL) to give a colorless suspension. Iodine 10 mg was added to initialize the reaction and refluxing for 30 mins. In a 250 mL two-neck round-bottomed flask was **1-chloroisoquinoline** (1.63 g, 10.00 mmol) and **$Ni(dppe)Cl_2$** (53 mg, 0.100 mmol) in dried THF (30 mL) to give a orange solution. The Grignard reagent of first solution was transferred by cannula at refluxing temperature, the resulting dark brown solution in the 250 mL flask was left stirring for 20 mins and refluxing for overnight. Monitoring of reaction shows product left without starting materials. H_2O was added to end the reaction. The aqueous layer was back extracted with Et_2O (50 mL×3). Combined the organic layers and washed with water (50 mL×2). The organic layer was dried by $MgSO_4$, filtrated and concentrated. The crude product was added to a silica gel column and was eluted with DCM/Hex (V/V from 1/1 to 3/1). Collect fractions with R_f = 0.25 in DCM/Hex (V/V =1/1). Purified **Htiq**: 1.94g, Yield: 94%.^{1}H NMR (400 MHz, $CDCl_3$)

δ = 8.54 (dd, J = 14.0, 7.1 Hz, 2H), 7.87 (d, J = 8.1 Hz, 1H), 7.71 (t, J = 7.3 Hz, 1H), 7.61 (dd, J = 12.8, 6.9 Hz, 3H), 7.54 (d, J = 4.7 Hz, 1H), 7.22 ppm (d, J = 4.1 Hz, 1H). **[(tiq)$_2$IrCl]$_2$**: yield: 92%.

Synthesis of bis-NHC carbene ligands:

Synthesis of diGluBr$_2$:

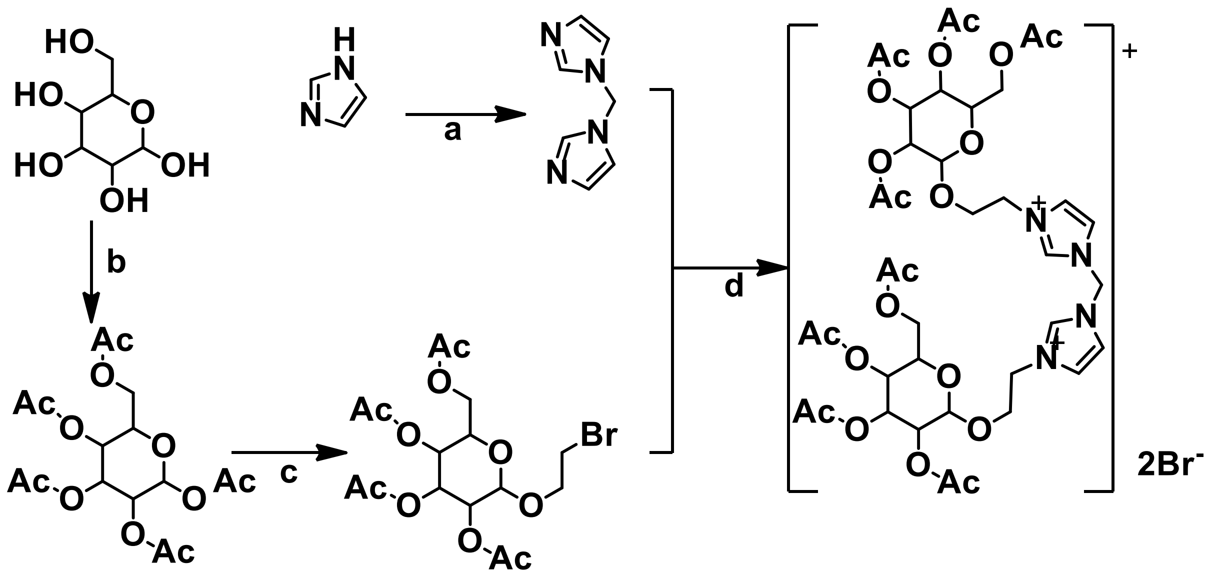

Scheme 4, a) NaH, CH_2Br_2, DMF; b), NaOAc, Ac_2O, reflux; c) $ZnCl_2$, $BrCH_2CH_2OH$, DCM, $BF_3{\cdot}Et_2O$, room temperature (r.t.); d) MeCN, reflux.

Synthesis of di(1H-imidazol-1-yl)methane:

In a 250 mL two-neck round-bottomed flask was 1H-imidazole (6.8 g, 100 mmol) in THF (200 mL) to give a colorless solution. Sodium hydride (4.39 g, 110 mmol) was added by three lots and resulting grey suspension was added dibromomethane (9.03 g, 51.9 mmol) by three lots under ice bath. The resulting grey suspension was heated at 50°C for overnight, and grey solid was converted to yellow. The reaction was monitored by ^{1}H NMR. Upon reaction was completed, the solvent was removed, and the residue was dissolved by 100 mL of methanol and passed through celite and washed by 50 mL of methanol. The filtrate was reduced and prepared to a silica gel column and was eluted with DCM/MeOH (V/V = 4/1 to 1/1). Collected yellow fractions with R_f = 0.25 (DCM/MeOH: V/V = 4/1) which could be stained in I_2 champer. ^{1}H NMR (400 MHz, $CDCl_3$) δ = 7.91 (s, 2H), 7.37 (s, 2H), 6.89 (s, 2H), 6.20 ppm (s, 2H).

Synthesis of GluOAc [6-(acetoxymethyl)tetrahydro-2H-pyran-2,3,4,5-tetrayl tetraacetate]:

The procedure is similar to reported literature.[11] To a solution of acetic anhydride (60mL) was added sodium acetate (16.2g, 197mmol, 3eq.). The mixture was refluxed at 90°C for 10mins to which glucose (12g, 66.6mmol, 1eq.) was added and stirred for 4 hrs. The organic solvent was removed under vacuum pump, dissolved into methanol and recrystallized with cold water. White precipitate was formed and filtrated and dried by air (22.72g, 58.2mmol, 87%). The product was used without further purification. ^{1}H NMR (400 MHz, $CDCl_3$) δ = 5.69 (d, J = 8.3 Hz, 1H), 5.23 (t, J = 9.4 Hz, 1H), 5.14 – 5.07 (m, 2H), 4.26 (dd, J = 12.5, 4.5 Hz, 1H), 4.10 – 4.04 (m, 1H), 3.82 (ddd, J = 10.0, 4.4, 2.2 Hz, 1H), 2.20 (s, 3H), 2.09 (s, 3H), 2.06 (s, 3H), 2.01 (s, 6H), 1.99 ppm (s, 3H).

Synthesis of GluOCH$_2$CH$_2$Br [2-(acetoxymethyl)-6-(2-bromoethoxy)tetrahydro-2H-pyran-3,4,5-triyl triacetate]:

To a dried two-neck flask was added $ZnCl_2$ (0.2 g, 1.47 mmol, 0.29 eq.), and the bottle was connected to a vacuum line and was heated by a hot gun to remove moisture which could be introduced in the procedure of weighting. To this flask, above **GluOAc** (2 g, 5.12 mmol, 1 eq.) and dichloromethane (HPLC, 30 mL), 2-bromoethanol (0.95 g, 7.6 mmol, 1.5 eq.) were added under nitrogen gas protection, followed by the addition of boron trifluoride diethyl etherate ($BF_3{\cdot}Et_2O$) (2.5g, 8.46 mmol, 1.65 eq.) dropwise at room temperature. The mixture was stirred under N_2 atmosphere for 6h at room temperature. Upon completion, the mixture was quenched by water, extracted by DCM (2×40 mL), concentrated. The crude product was added into a silica gel column and was eluted by EA/Hex (V/V = 1/3). The fractions of R_f = 0.25 was collected, and the spot on thin layer silica plate was shown by using the following procedure: the whole plate was wetted by a solution of EtOH/AcOH/H_2SO_4/4-MeOPhCHO (V/V/V/V = 135/1.5/5/4.7)

and heated by hot gun. After remove all solvent, a white solid was formed (1.32 g, 2.9 mmol, yield of 56.6%). ^{1}H NMR (400 MHz, $CDCl_3$) δ = 5.22 (t, J = 9.5 Hz, 1H), 5.08 (t, J = 9.7 Hz, 1H), 5.01 (dd, J = 9.6, 8.0 Hz, 1H), 4.57 (d, J = 8.0 Hz, 1H), 4.26 (dd, J = 12.3, 4.7 Hz, 1H), 4.19 – 4.12 (m, 2H), 3.85 – 3.78 (m, 1H), 3.71 (ddd, J = 9.9, 4.7, 2.4 Hz, 1H), 3.48 – 3.43 (m, 2H), 2.09 (s, 3H), 2.07 (s, 3H), 2.02 (s, 3H), 2.01 ppm (s, 2H).

Synthesis of diGluBr$_2$ [1,1'-methylenebis(3-(2-((3,4,5-triacetoxy-6-(acetoxymethyl)tetrahydro-2H-pyran-2-yl)oxy)ethyl)-1H-imidazol-3-ium) dibromide]:

Above $GluOCH_2CH_2Br$ was used without further purification. To a flask was added $GluOCH_2CH_2Br$ (1.32 g, 2.9 mmol, 2.1 eq.) and di(1H-imidazol-1-yl)methane (0.205g, 1.381 mmol, 1eq.) into MeCN. The mixture was refluxing for 2 days, the organic solvent is removed under reduce pressure. Resulting crude product was dissolved into DCM (5 mL), add Et_2O (20 mL) to precipitate the desired product. This procedure was repeated for 2 to 3 times to remove unreacted $GluOCH_2CH_2Br$. 1.2g of product was collected with the yield of 82%. ^{1}H NMR (400 MHz, $CDCl_3$) δ = 9.41 (s, 2H), 8.03 (s, 2H), 7.74 (s, 2H), 6.73 (s, 2H), 5.24 (t, J = 9.6 Hz, 2H), 4.89 (t, J = 9.7 Hz, 2H), 4.83 (t, J = 8.7 Hz, 2H), 4.79 – 4.73 (m, 2H), 4.55 – 4.37 (m, 4H), 4.13 (dd, J = 12.3, 5.2 Hz, 2H), 4.06 – 3.97 (m, 6H), 3.95 – 3.88 (m, 2H), 2.01 (s, 6H), 1.97 (s, 6H), 1.92 (s, 6H), 1.90 ppm (s, 6H).

Visible-light-driven radical cyclization

Synthesis of substrates:

Substrate A:

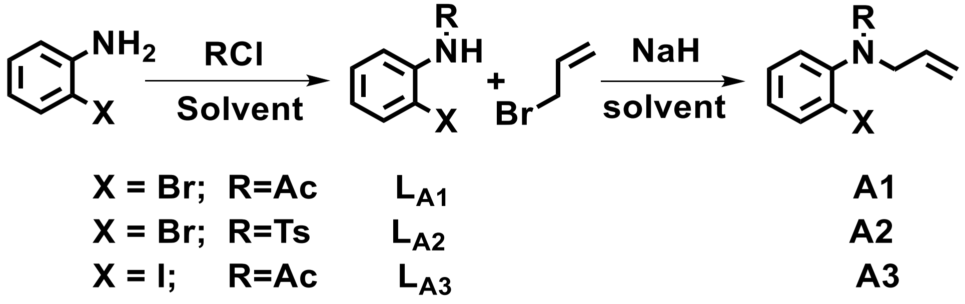

Substrate B:

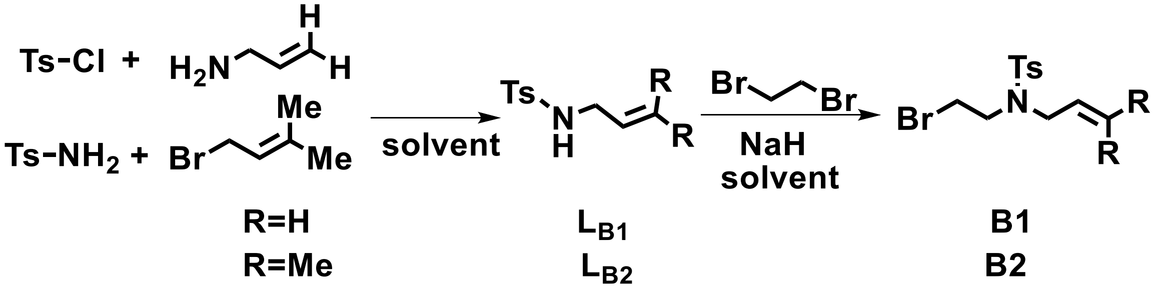

General procedure for visible light-induced photocatalytic reductive cyclization of organohalides (Table 3 – 7):

Use substrate **A1**, photo-catalyst **4^{Me}b** and N,N-diisopropylethylamine (DIPEA) as example: To a test tube (Pyrex, 15×125 mm) charged with an organohalide substrate (50 μmol) and 2mol% of **4^{Me}b** complexes (1 μmol) were added MeCN (4 mL), 5eq of DIPEA (87 μL) and 2.5eq of HCOOH (10 μL). The mixture in test tube was degassed by nitrogen (bubbling for 10 min through septa by cannula). The test tube was placed in the irradiation apparatus equipped with blue LEDs (centred at 460 nm, 12 w).[12]. The resulting mixture was stirred at ambient temperature for specified time. The organic solvent was evaporated under reduced pressure. Then added 30 mL of Et_2O and followed with 10 mL of saturated $NaHCO_3$ (aq) solution. The mixture was extracted by Et_2O (15 mL×2) and combined organic solvent was washed by water (15 mL×2), dried over $MgSO_4$. After organic solvent is removed, the yield of product can be calculated by adding internal standard 5,5'-dimethyl-2,2'-bipyridine (with known weight) to the resulting crude residue.

Table S4. The characterization of substrates and products in photocatalysis.

Substrates and solvent system for synthesis	Synthesis procedures and characterization of NMR data
**L_{A1}:** (TEA as base, DCM)[13] 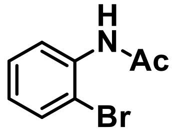	Synthesis procedure is following the reported procedure.[13] ^{1}H NMR (300 MHz, $CDCl_3$) δ = 8.34 (d, J = 8.4 Hz, 1H), 7.54 (d, J =7.8 Hz, 1H), 7.32 (t, J = 7.8 Hz, 1H), 6.98 (t, J = 7.5 Hz, 1H), 2.24 ppm (s, 3H).
**L_{A2}:** (Water; r.t.) 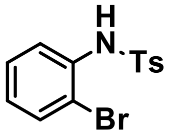	Synthesis procedure is following the reported procedure.[14, 15] ^{1}H NMR (300 MHz, $CDCl_3$) δ = 7.66 (t, J = 7.3 Hz, 3H), 7.41 (d, J = 7.8 Hz, 1H), 7.30 - 7.25 (m, 1H), 7.22 (d, J = 8.3 Hz, 2H), 6.97 (t, J = 7.0 Hz, 2H), 2.38 ppm (s, 3H).
**L_{A3}:** (TEA as base, DCM) 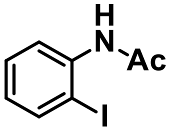	Synthesis procedure is following the reported procedure.[13] ^{1}H NMR (400 MHz, $CDCl_3$) δ = 8.21 (d, J = 7.8 Hz, 1H), 7.78 (d, J = 7.4 Hz, 1H), 7.35 (t, J = 7.8 Hz, 1H), 6.85 (t, J = 7.4 Hz, 1H), 2.25 ppm (s, 3H).
**A1**: (dried THF) 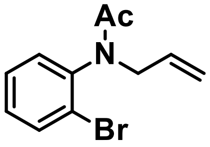	Synthesis procedure is following the reported procedure.[1] ^{1}H NMR (400 MHz, $CDCl_3$) δ = 7.69 (dd, J = 8.0, 1.3 Hz, 1H), 7.36 (td, J = 7.6, 1.4 Hz, 1H), 7.22 (td, J = 9.0, 1.5 Hz, 2H), 5.94 – 5.82 (m, 1 H), 5.06 (ddt, J = 15.6, 10.1, 1.3 Hz, 2H), 4.78 (ddt, J = 14.7, 5.7, 1.2 Hz, 1H), 3.72 (dd, J = 14.7, 7.6Hz, 1H), 1.81 ppm(s, 3H).
**A2:** (dried THF) 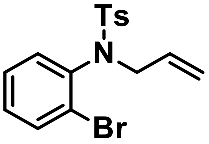	Synthesis procedure is following the reported procedure with slightly change of solvent system (THF).[16] ^{1}H NMR (400 MHz, $CDCl_3$) δ = 7.66 (d, J = 8.3 Hz, 2H), 7.60 (dd, J = 7.9, 1.5 Hz, 1H), 7.28 (d, J = 8.0 Hz, 2H), 7.27 – 7.21 (m, 1H), 7.17 (td, J = 7.7, 1.7 Hz, 1H), 7.08 (dd, J = 7.8, 1.6 Hz, 1H), 5.83 (ddt, J = 16.9, 10.1, 6.7 Hz, 1H), 5.07 – 4.95 (m, 2H), 4.23 – 4.13 (m, 2H), 2.43 (s, 3H).
**A3**: (dried THF) 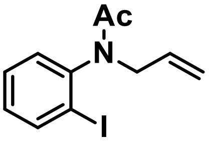	Synthesis procedure is following the reported procedure.[1] ^{1}H NMR (300 MHz, $CDCl_3$) δ = 7.95 (dd, J = 7.9, 1.4 Hz, 1H), 7.39 (td, J = 7.6, 1.4 Hz, 1H), 7.18 (dd, J = 7.8, 1.6 Hz, 1H), 7.08 (td, J = 7.7, 1.6 Hz, 1H), 5.90 (dddd, J = 17.7, 10.1, 7.8, 5.5 Hz, 1 H), 5.16 – 4.99 (m, 2H), 4.85 (ddt, J = 14.7, 5.5, 1.2 Hz, 1H), 3.59 (dd, J = 14.7, 7.8 Hz, 1H), 1.80 ppm (s, 3 H).
**P_{A1} = P_{A3}**: (MeCN) 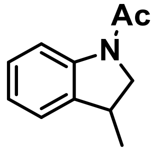	NMR spectrum matches to reported literature.[17] ^{1}H NMR (400 MHz, $CDCl_3$) δ = 8.19 (d, J = 8.1 Hz, 1H), 7.20 (t, J = 7.7 Hz, 1H), 7.15 (d, J = 7.4 Hz, 1H), 7.03 (t, J = 7.4 Hz, 1H), 4.20 (t, J = 9.5 Hz, 1H), 3.57 (dd, J = 9.8, 6.8 Hz, 1H), 3.49 (dd, J = 15.4, 7.0 Hz, 1H), 2.21 (s, 3H), 1.35 ppm (d, J = 6.8 Hz, 3H).
**P_{A2}**: (MeCN) 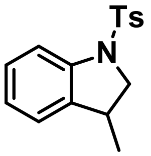	NMR spectra match to reported literature.[17] ^{1}H NMR (400 MHz, $CDCl_3$) δ = 7.68 (d, J = 8.3 Hz, 2H), 7.63 (d, J = 8.1 Hz, 1H), 7.25 – 7.18(m, 3H), 7.05 (d, J = 7.3 Hz, 1H), 6.99 (t, J = 7.3 Hz, 1H), 4.08 (dd, J = 10.4, 9.1 Hz, 1H), 3.41 (dd, J = 10.4, 7.2 Hz, 1H), 3.19 (dd, J = 15.4, 7.3 Hz, 1H), 2.36 (s, 3H), 1.11 ppm (d, J = 6.9 Hz, 3H). ^{13}C NMR (101 MHz, $CDCl_3$) δ = 144.0, 141.6, 136.8, 134.2, 129.6, 127.9, 127.4, 123.9, 123.8, 114.9, 57.6, 34.7, 21.5, 19.5 ppm.
**L_{B1}**: (TEA as base, CH_2Cl_2) 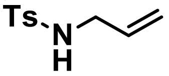	Synthesis procedure is following the reported procedure.[17, 18] ^{1}H NMR (300 MHz, $CDCl_3$) δ = 7.76 (d, J = 8.3 Hz, 2H), 7.32 (d, J = 8.0 Hz, 2H), 5.73 (ddt, J = 17.1, 11.2, 5.8 Hz, 1H), 5.25 – 5.06 (m, 2H), 3.59 (tt, J = 6.0, 1.5 Hz, 2H), 2.44 ppm (s, 3H,).

L_{B2}: (K_2CO_3 as base, Acetone)

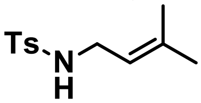

Synthesis procedure is following the reported procedure.[18] ^{1}H NMR (400 MHz, $CDCl_3$) δ = 7.75 (d, *J* = 8.1 Hz, 2H), 7.30 (d, *J* = 7.9 Hz, 2H), 5.05 (t, *J* = 7.0 Hz, 1H), 3.53 (t, *J* = 6.3 Hz, 2H), 2.43 (s, 3H), 1.63 (s, 3H), 1.53 ppm (s, 3H).

B1: (DMF)

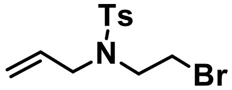

Synthesis procedure is following the reported procedure.[17] ^{1}H NMR (300 MHz, $CDCl_3$) δ = 7.71 (d, *J* = 8.2, 2H), 7.33 (d, *J* = 8.4, 2H), 5.76 – 5.60 (m, 1H), 5.25 – 5.15 (m, 1H), 3.82 (d, *J* = 6.5 Hz, 2H), 3.53 – 3.35 (m, 4H), 2.44 ppm (s, 3H).

B2: (DMF)

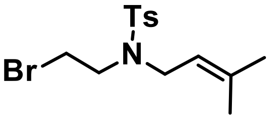

Synthesis procedure is following the reported procedure.[17] ^{1}H NMR (400 MHz, $CDCl_3$) δ = 7.70 (d, *J* = 7.9 Hz, 2H), 7.32 (d, *J* = 7.8 Hz, 2H), 5.02 (t, *J* = 7.2 Hz, 1H), 3.80 (d, *J* = 7.0 Hz, 2H), 3.50 – 3.34 (m, 4H), 2.44 (s, 3 H), 1.69 (s, 3H), 1.63 ppm (s, 3H).

P_{B1}: (MeCN)

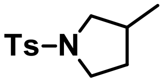

NMR spectra match to reported literature.[17] ^{1}H NMR (400 MHz, $CDCl_3$) δ = 7.70 (d, *J* = 8.2 Hz, 2H), 7.31 (d, *J* = 8.0 Hz, 2H), 3.41 (dd, *J* = 9.6 Hz, 7.2, 1H), 3.33 (ddd, *J* = 9.8, 8.3, 4.2 Hz, 1H), 3.21 (ddd, *J* = 9.8, 8.2, 7.4 Hz, 1H), 2.74 (dd, *J* = 9.6, 7.8 Hz, 1H), 2.11 (dd, *J* = 14.7, 7.6 Hz, 1H), 1.94 – 1.84 (m, 1H), 1.34 (ddd, *J* = 16.7, 12.4, 8.3 Hz, 1H), 0.91 ppm (d, *J* = 6.7 Hz, 3H). ^{13}C NMR (101 MHz, $CDCl_3$) δ = 143.2, 134.1, 129.5, 127.4, 54.7, 47.5, 33.2, 29.6, 21.4, 17.6 ppm.

P_{B2}: (MeCN)

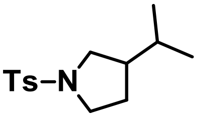

^{1}H NMR spectrum matches to reported literature.[17] ^{1}H NMR (400 MHz, $CDCl_3$) δ = 7.70 (d, *J*=8.2, 2H), 7.31 (d, *J* = 8.0 Hz, 2H), 3.44 (dd, *J* = 9.6, 7.7 Hz, 1H), 3.40 – 3.33 (m, 1H), 3.15 (td, *J* = 9.9, 6.8 Hz, 1H), 2.78 (t, *J* = 9.6 Hz, 1H), 2.43 (s, 3H), 1.97 – 1.83 (m, 2H), 1.65 (m, 1H), 1.30 – 1.47 (m, 1H), 0.83 ppm (d, *J* = 6.6 Hz, 6H).

Visible-light-driven CO_2 Reduction

Photo-catalytic procedure of CO_2 reduction

In a 4 mL CH_3CN/TEA (4:1, v/v; TEA = triethylamine) solution, [Co(TPA)Cl]Cl and [Ir] (**4^{Me}b**,OTf anion) (with specified concentration) was added into a Pyrex tube (Volume: 22 mL (16 (OD.)×150; 1.2 thickness (mm)) and purged with CO_2 through a septum (purity ⩾ 99.8%) for 10 min, followed by 250 μL CH_4 was injected to the tube prior to the irradiation using blue LEDs (centred at 460 nm, 12 w).[12] All reactions and LEDs were cooled by aluminium blocks by using cooling fans. Gas sample (200 μL) was drawn from the headspace of the tube and injected to GC-TCD for measurement.

Measurement of gases products

Gas chromatographic analysis was conducted using Agilent 7890A gas chromatography equipped with a thermal conductivity detector (TCD) and a HP-Plot 5Å column with Ar as the carrier gas. The oven temperature was held at 40 °C. Inlet and detector temperature were set at 80 °C and 150 °C respectively. Calibration curves were established separately based on the averaged results from three point of injections for CO (R^2 = 0.9997), H_2 (R^2 = 1.0000) and CH_4 (R^2 = 0.9996).

Table S5, Control experiments

**Starting**			**Product**	
**4^{Me}b**	**[Co(TPA)Cl]Cl**	**TEA**	**H_2**	**CO**
-	+	+	-	-
+	-	+	+	-
+	+	-	+	-
+ (in dark)	+	+	-	-

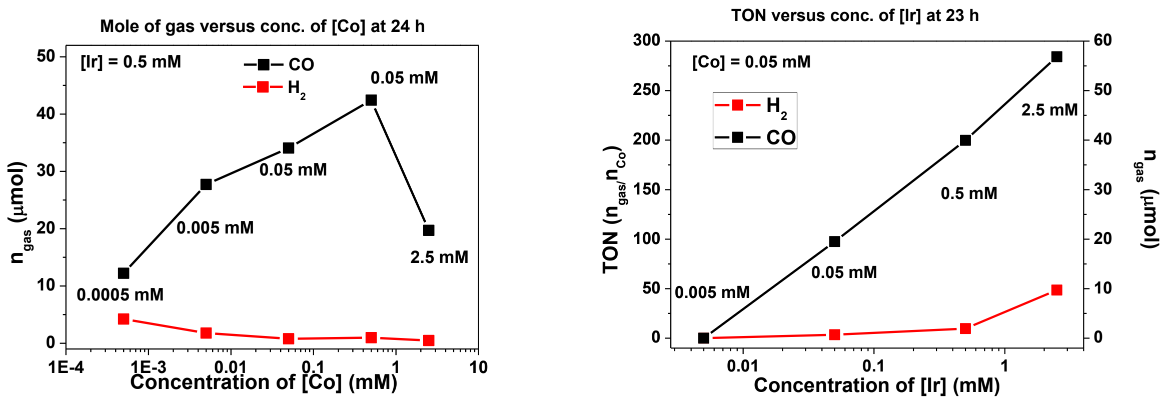

Fig. S11 Plots of moles of gases produced versus concentration of left) **[Co(TPA)Cl]Cl**; right) **4^{Me}b**.

Measurements of quenching constants for [Co(TPA)Cl]Cl to 4^{Me}b:

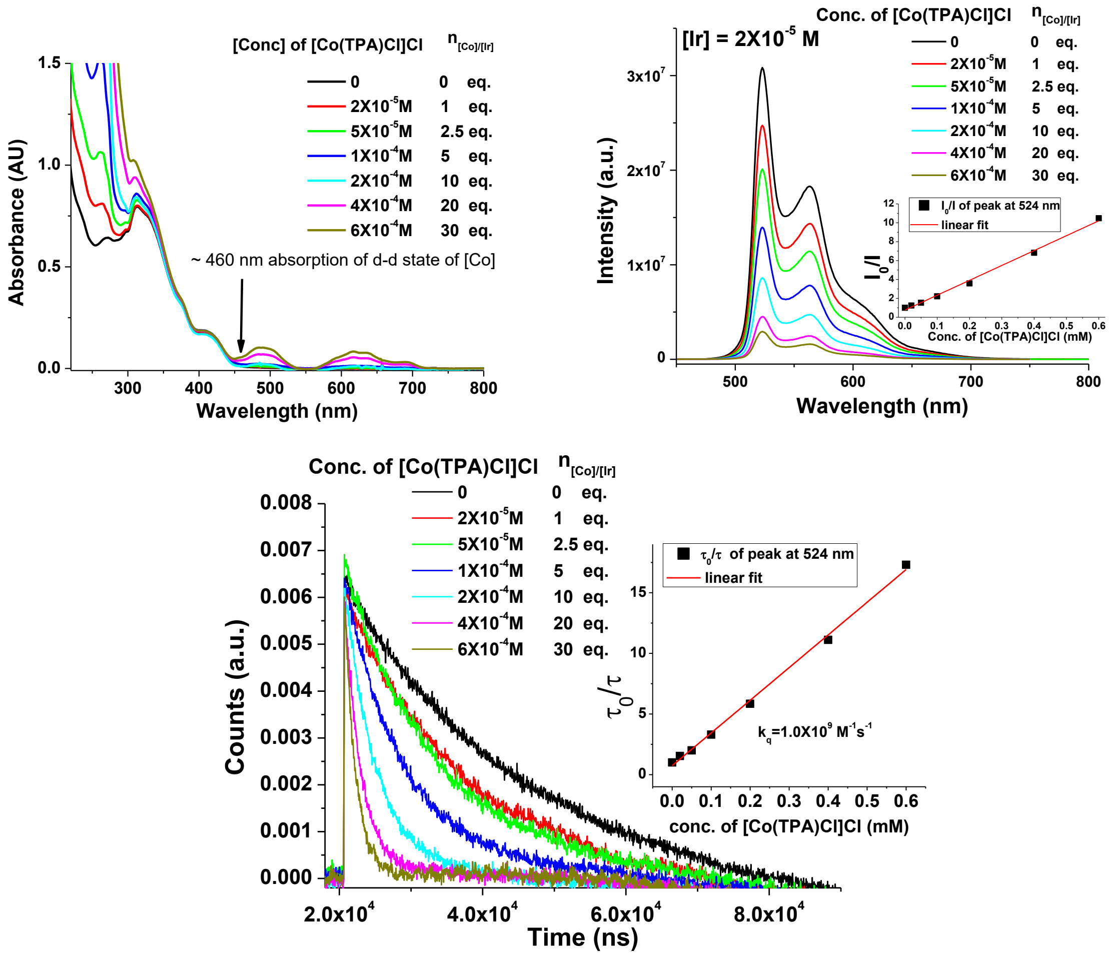

Fig. S12 Stern–Volmer behaviours of **4^{Me}b** (concentration of 2×10^{-5} M for all solutions) in the presence of **[Co(TPA)Cl]Cl** in degassed acetonitrile solution: upper) UV spectra; middle) emission spectra with identical conditions of excitation (insert, linear plots (R^2 = 0.9946) for I_0/I (intensities of λ_{em} = 524 nm for each solution)); bottom) lifetime decay plots (insert, linear plots (R^2 = 0.9969) for τ_0/τ of peaks of λ_{em} = 524 nm for each solution) and quenching constant (K_q) is calculated as $1.0\times10^9 M^{-1}s^{-1}$.

Biological studies:

Anticancer properties:

The cell lines were maintained in cell culture media (minimum essential medium for HeLa) supplemented with 10% fetal bovine serum, 100 U/mL penicillin, and 100 μg/mL streptomycin at 37°C humidified atomsphere with 5% CO_2. Cell growth inhibitory effects of the iridium(III) complexes and cisplatin were determined by MTT cytotoxicity assay. Drug treated cells were incubated with MTT for 12h at 37°C in a humidified atmosphere of 5% CO_2 and were subsequently lysed in solubilizing solution. Cells were then maintained in a dark, humidified chamber overnight. The formation of formazan was measured by using a microtitre plate reader at 580 nm. Growth inhibition by a drug was evaluated by IC_{50} (concentration of a drug causing 50% inhibition of cell growth). Each growth inhibition experiment was repeated at least three times and the results were expressed as means ± standard deviation (SD).

Table S6. IC50 (μM) values[a] of test compounds towards HeLa cell line.

Complex	**1a**	**2a**	**1b**	**6^H^b**	**6^F^b**	**7b**	**8b**	**1c**	**6^H^c**	**7c**	**9c**	cisplatin
**HeLa**	14.4 ± 3.5	8.3 ± 1.2	1.6 ± 0.3	1.7 ± 0.4	1.8 ± 0.7	1.7 ± 0.5	0.5 ± 0.3	15.2 ± 5.8	56.1 ± 24.7	53.4 ± 10.1	36.9 ± 15.8	20 ± 7.6

[a] IC_{50} values are drug concentrations necessary for 50% inhibition of cell viability. Cells are treated with the complexes for 72 h.

Fluorescence microscopic examination

HeLa cells (2×10^5 cells) were seeded in a one chamber slide (Nalgene; Nunc) with culture medium (2 mL per well) and incubated at 37°C in a humidified atmosphere of 5% CO_2/95% air for 24 h. A stock solution of iridium complex was prepared in DMSO and then diluted to 5μM into the cells glass coverlips (Mattek 35mm glass bottom dish) for imaging experiments. After treating with Ir complexes only or the mixture of Ir complexes with ER-TrackerTM (1μM) or Lysotracker® (100 nM) or Mitotracker® (50 nM) for 15 mins, cells were directly exposed for fluorescent imaging without removing the old medium. The images were taken on Carl Zeiss LSM 710 microscope.

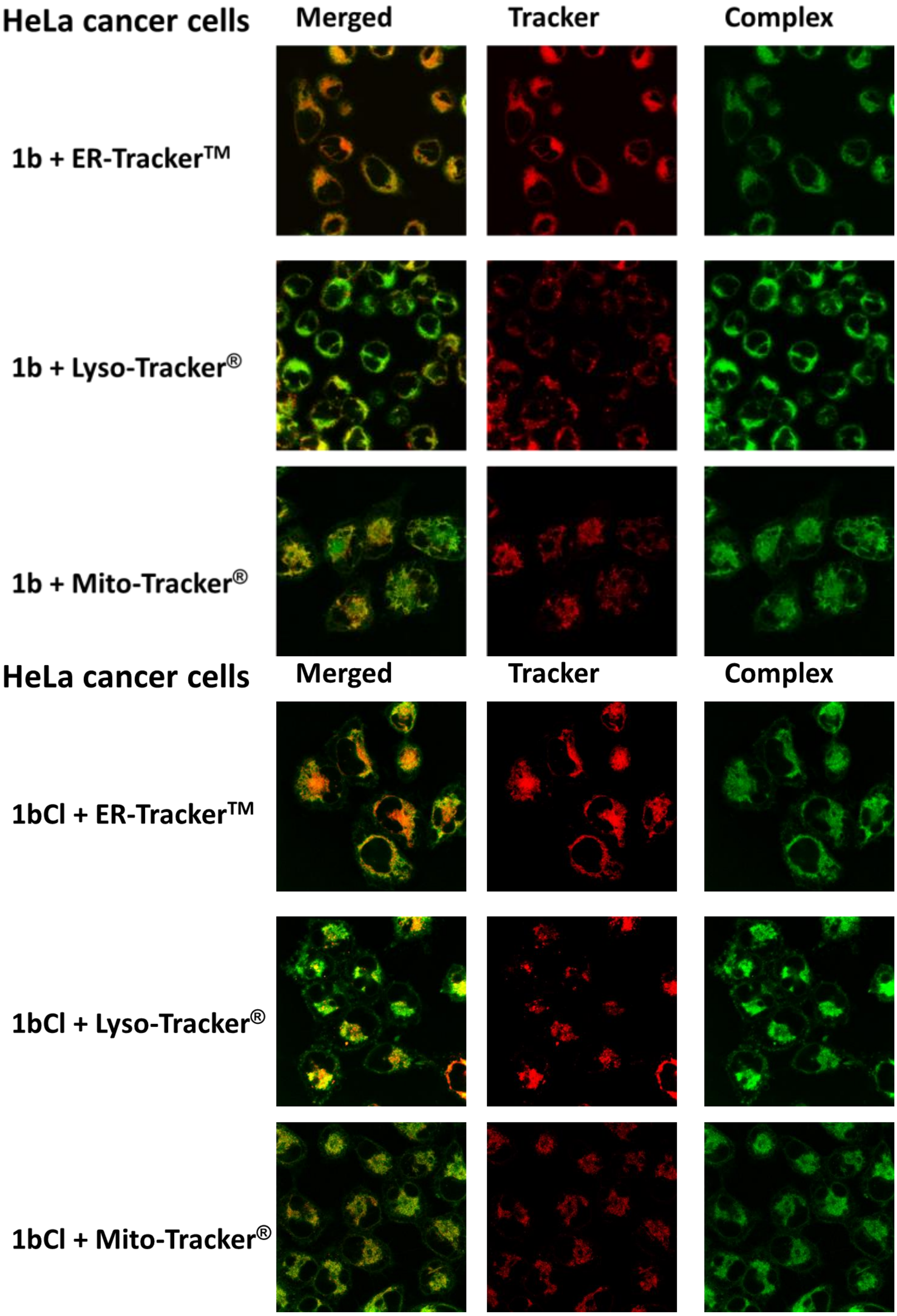
HeLa cancer cells
Merged
Tracker
Complex
1b + ER-Tracker™
1b + Lyso-Tracker®
1b + Mito-Tracker®
HeLa cancer cells
Merged
Tracker
Complex
1bCl + ER-Tracker™
1bCl + Lyso-Tracker®
1bCl + Mito-Tracker®

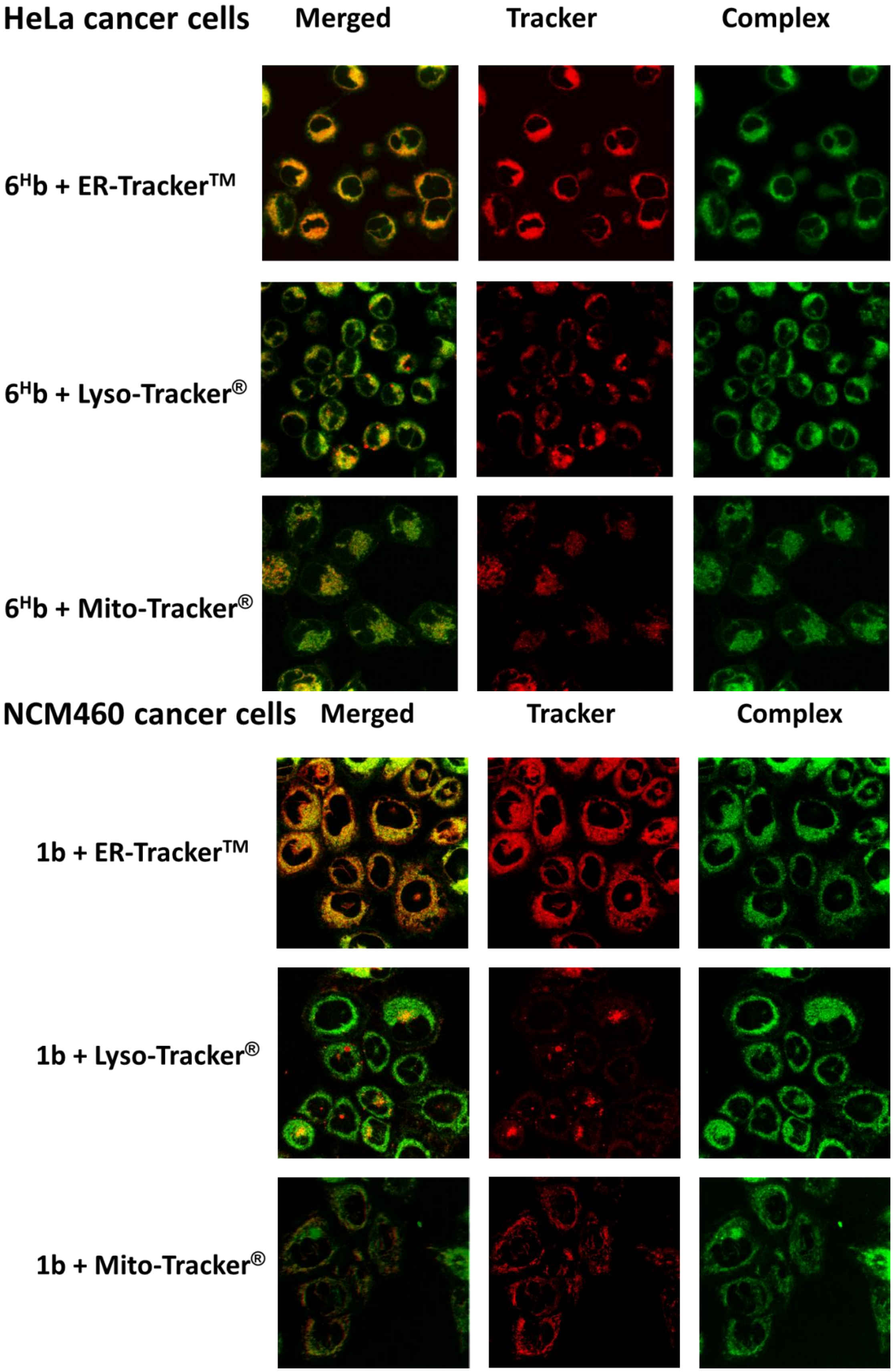
HeLa cancer cells
Merged
Tracker
Complex
6Hb + ER-Tracker™
6Hb + Lyso-Tracker®
6Hb + Mito-Tracker®
NCM460 cancer cells
Merged
Tracker
Complex
1b + ER-Tracker™
1b + Lyso-Tracker®
1b + Mito-Tracker®

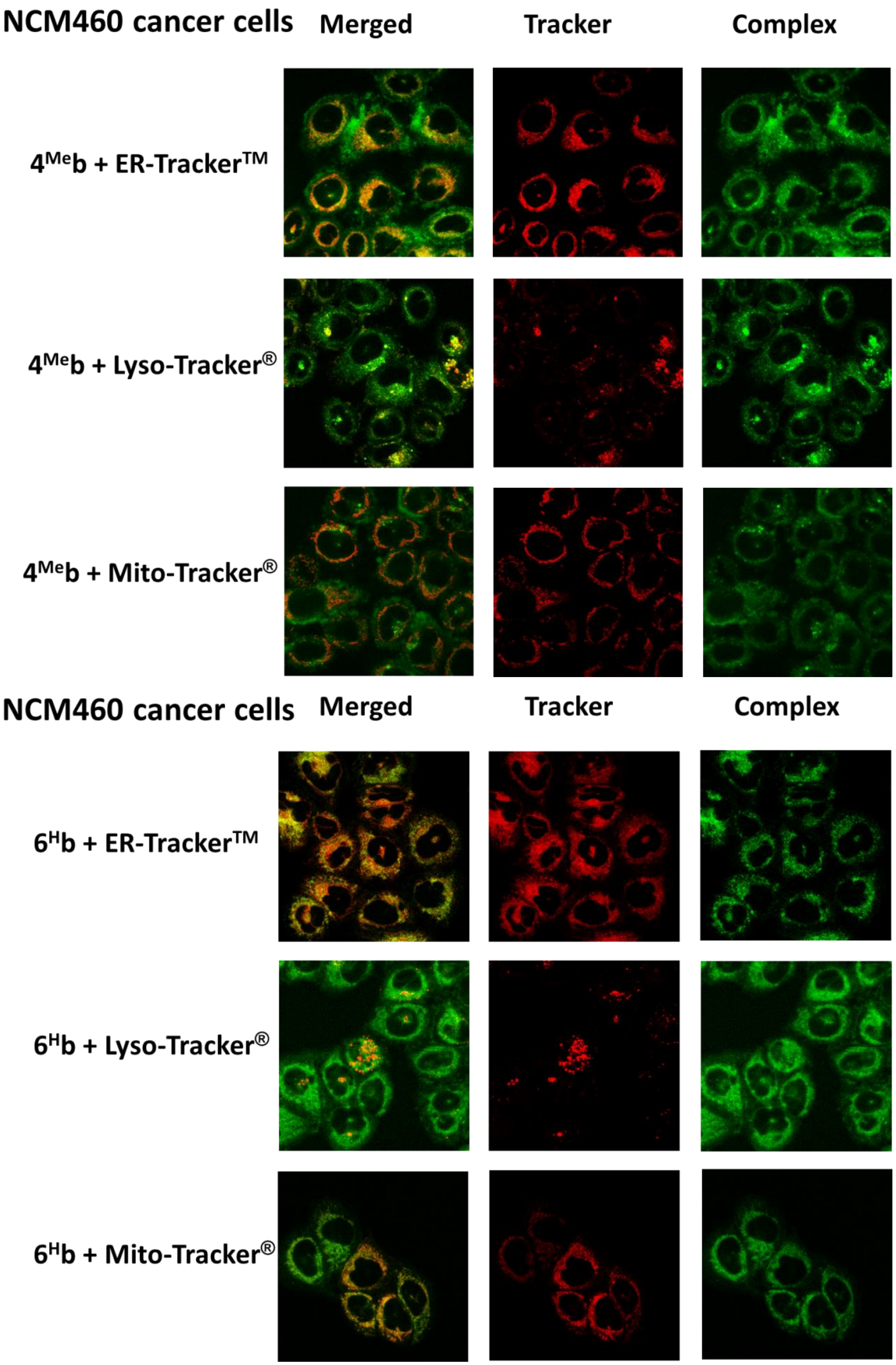
NCM460 cancer cells
Merged
Tracker
Complex
4^Meb + ER-Tracker™
4^Meb + Lyso-Tracker®
4^Meb + Mito-Tracker®
NCM460 cancer cells
Merged
Tracker
Complex
6^Hb + ER-Tracker™
6^Hb + Lyso-Tracker®
6^Hb + Mito-Tracker®

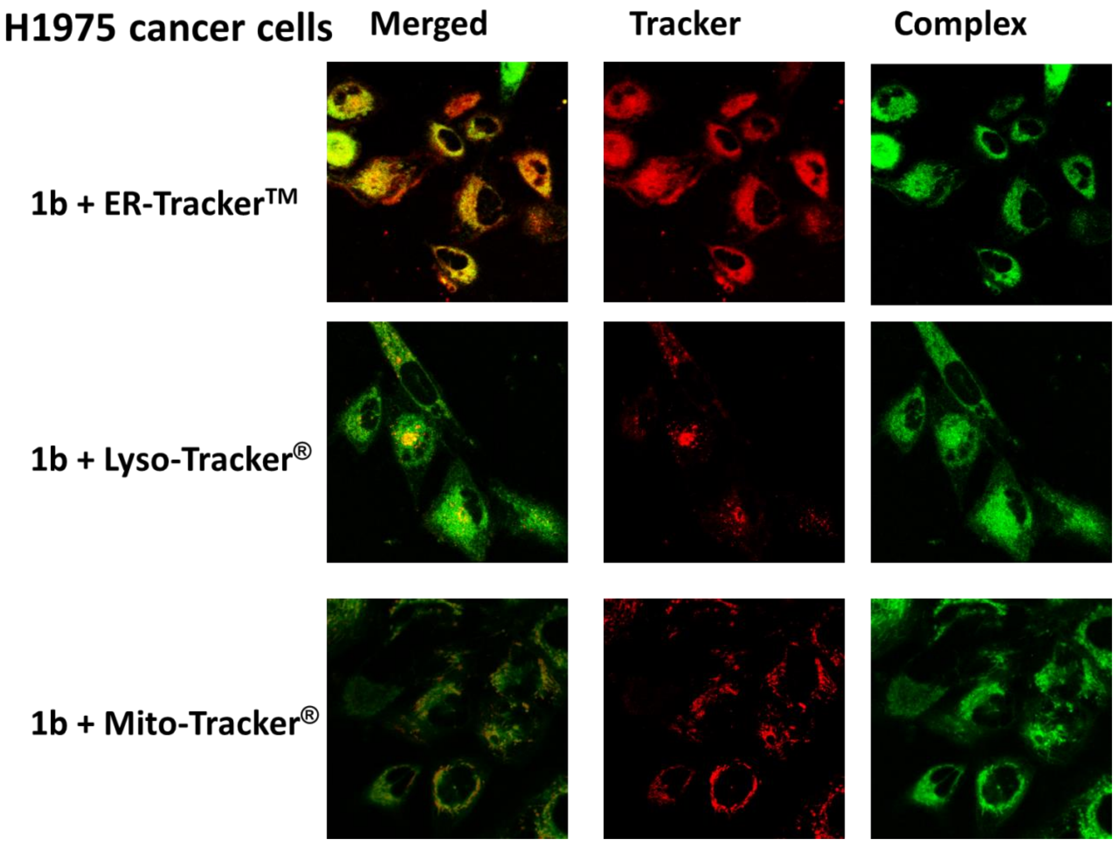
H1975 cancer cells
Merged
Tracker
Complex
1b + ER-Tracker™
1b + Lyso-Tracker®
1b + Mito-Tracker®

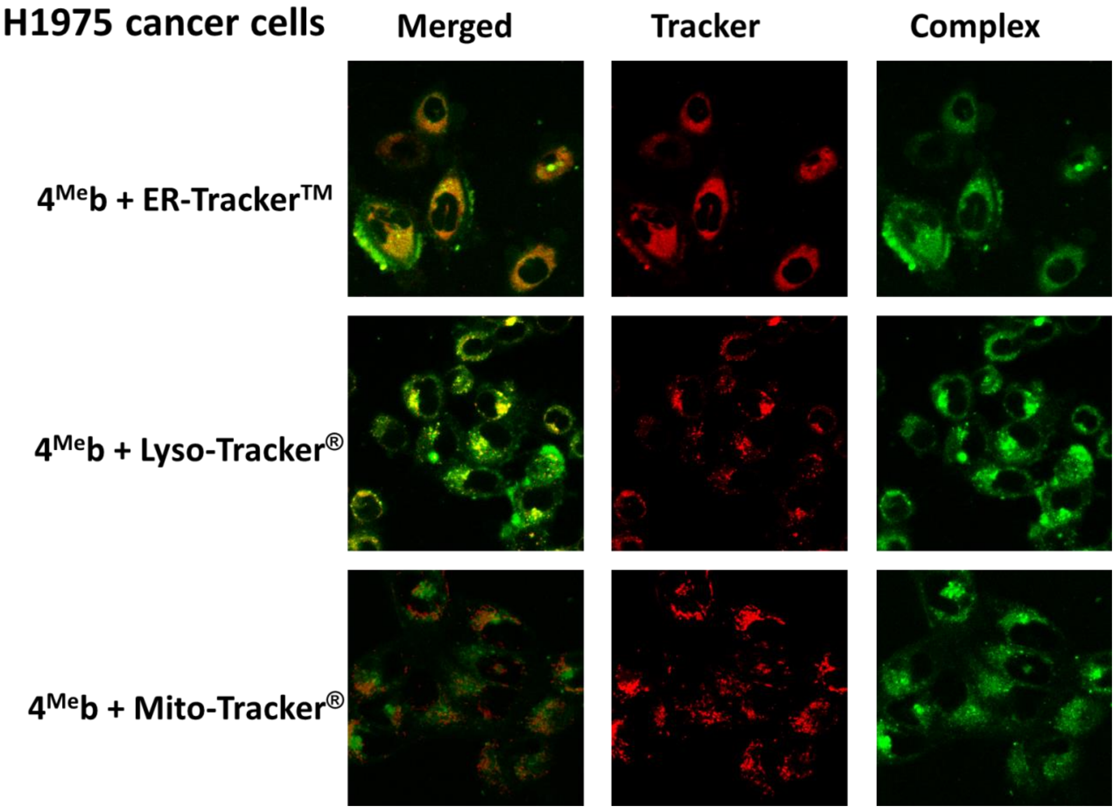
H1975 cancer cells
Merged
Tracker
Complex
4Meb + ER-Tracker™
4Meb + Lyso-Tracker®
4Meb + Mito-Tracker®

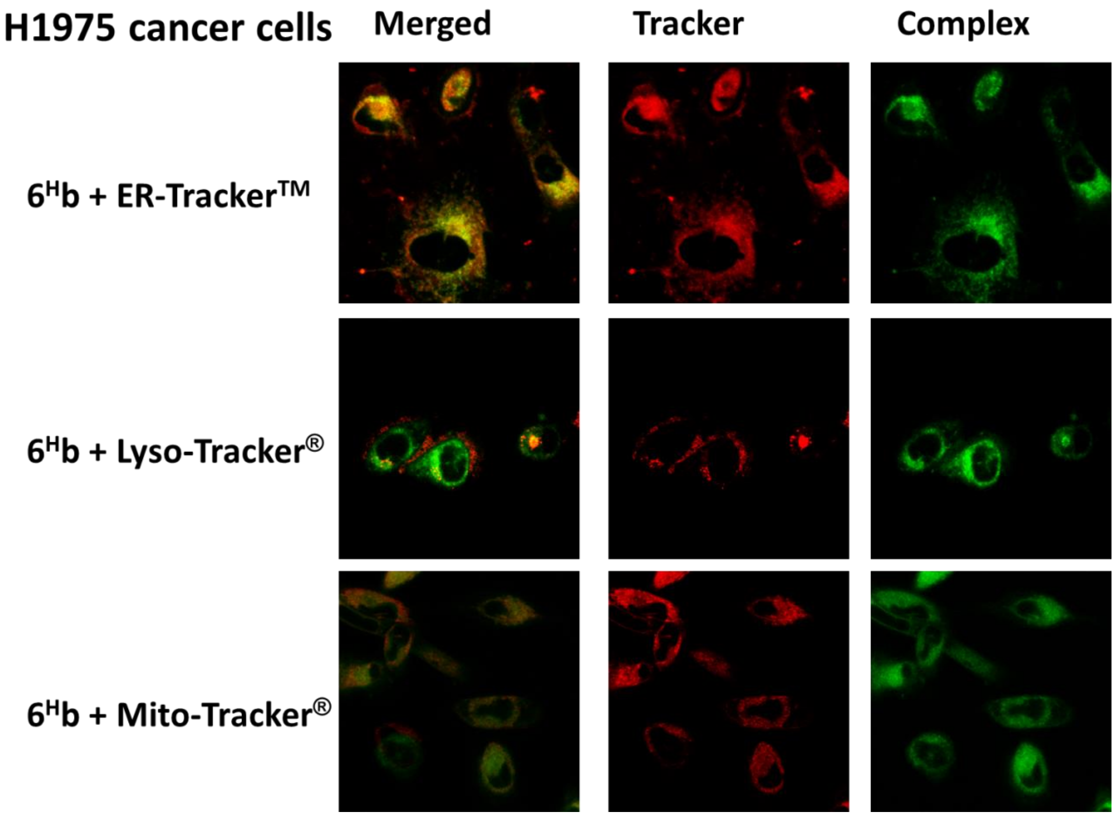

Fig. S13. Intracellular localization of **1b, 1bCl, 4^{Me}b** and **6^{H}b** into HeLa, NCM460 and H1975 cancer cells. Complexes were excited at 340 nm using an emission filter of 510 nm. ER-tracker^{EM} was excited at 546 nm using an emission filter of >580 nm.

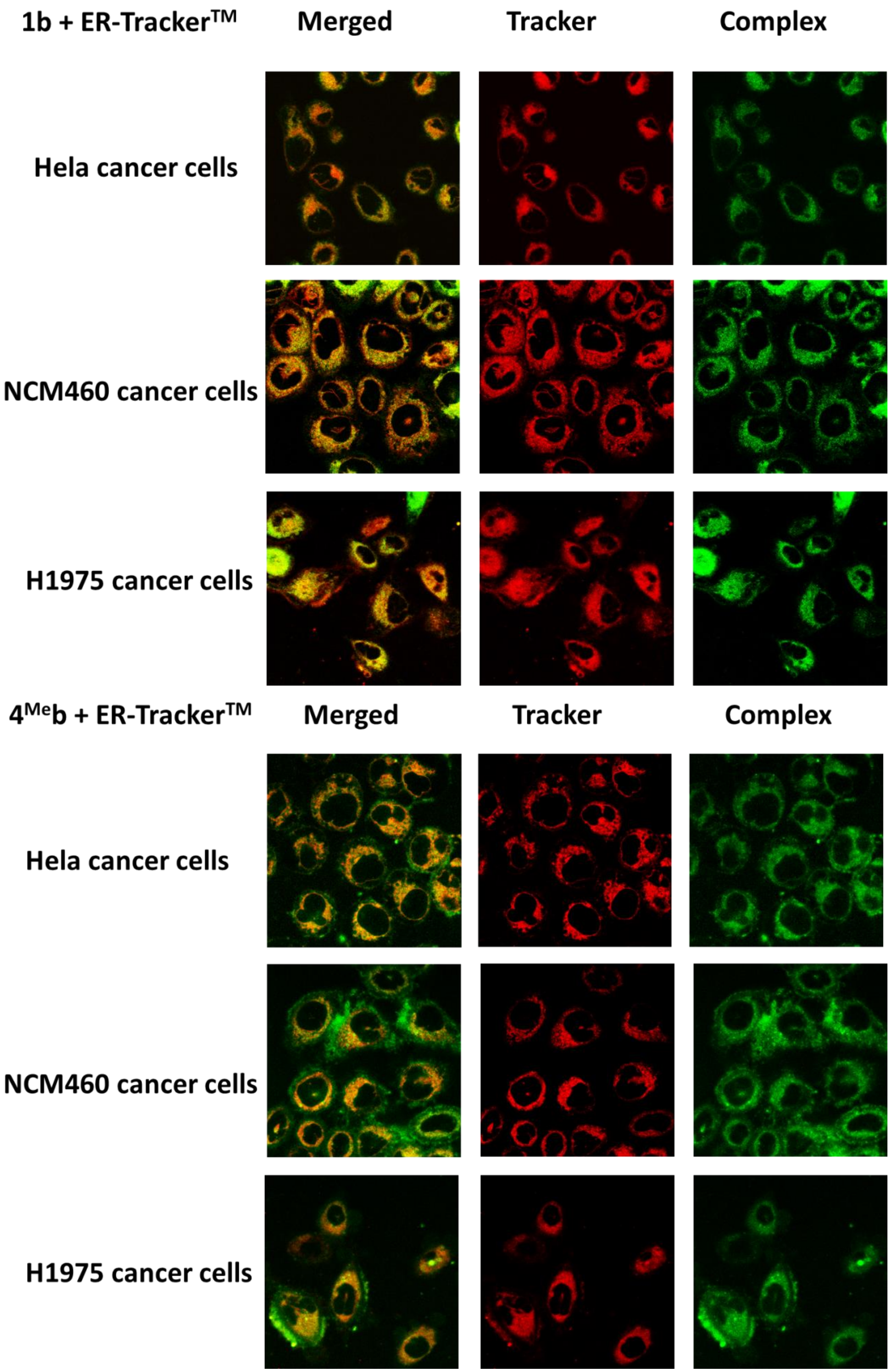
1b + ER-Tracker™
Merged
Tracker
Complex
Hela cancer cells
NCM460 cancer cells
H1975 cancer cells
4^{Me}b + ER-Tracker™
Merged
Tracker
Complex
Hela cancer cells
NCM460 cancer cells
H1975 cancer cells

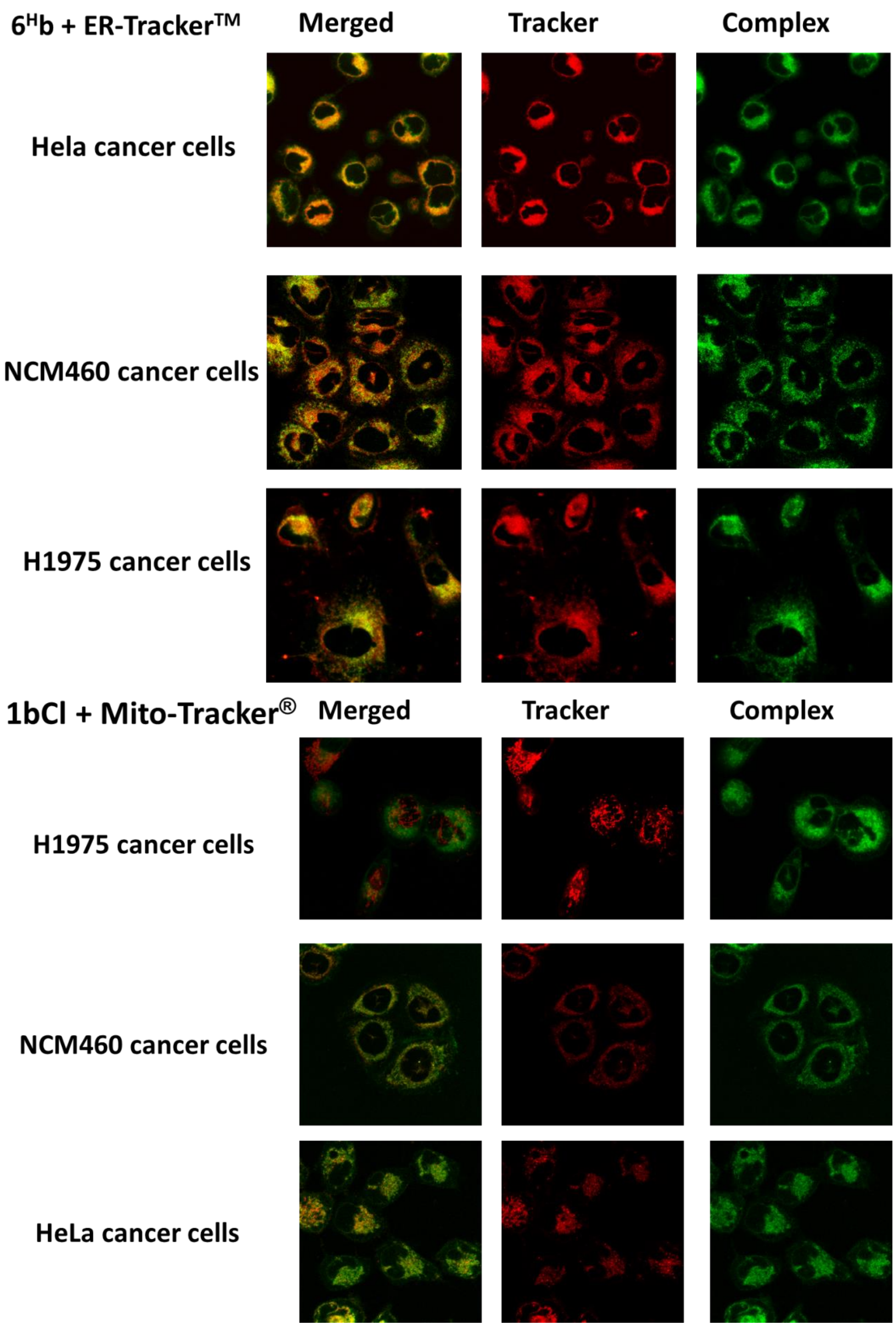

Fig. S14. Intracellular localization of **1b, 4^{Me}b** and **6^{H}b** with addition of ER-tracker™, as well as **1bCl** with Mito-tracker® into HeLa, NCM460 and H1975 cancer cells. Complexes were excited at 340 nm using an emission filter of 510 nm. ER-tracker^{EM} was excited at 546 nm using an emission filter of >580 nm.

1. H. Kim and C. Lee, *Angewandte Chemie International Edition*, 2012, **51**, 12303-12306.
2. J. W. Tucker and C. R. J. Stephenson, *The Journal of Organic Chemistry*, 2012, **77**, 1617-1622.
3. C. Shi, H. Sun, Q. Jiang, Q. Zhao, J. Wang, W. Huang and H. Yan, *Chemical Communications*, 2013, **49**, 4746-4748.
4. G. A. Crosby and J. N. Demas, *The Journal of Physical Chemistry*, 1971, **75**, 991-1024.
5. O. V. Dolomanov, L. J. Bourhis, R. J. Gildea, J. A. K. Howard and H. Puschmann, *Journal of Applied Crystallography*, 2009, **42**, 339-341.
6. G. Sheldrick, *Acta Crystallographica Section A*, 2008, **64**, 112-122.
7. R. Wang, D. Liu, H. Ren, T. Zhang, H. Yin, G. Liu and J. Li, *Advanced Materials*, 2011, **23**, 2823-2827.
8. C.-H. Yang, J. Beltran, V. Lemaur, J. r. m. Cornil, D. Hartmann, W. Sarfert, R. Fröhlich, C. Bizzarri and L. De Cola, *Inorganic Chemistry*, 2010, **49**, 9891-9901.
9. M. Ghiaci and J. Asghari, *Bulletin of the Chemical Society of Japan*, 2001, **74**, 1151-1152.
10. Q. Zheng, S. K. Gupta, G. S. He, L.-S. Tan and P. N. Prasad, *Advanced Functional Materials*, 2008, **18**, 2770-2779.
11. A. Pulsipher and M. N. Yousaf, *Chemical Communications*, 2011, **47**, 523-525.
12. P.-K. Chow, G. Cheng, G. S. M. Tong, W.-P. To, W.-L. Kwong, K.-H. Low, C.-C. Kwok, C. Ma and C.-M. Che, *Angewandte Chemie International Edition*, 2015, **54**, 2084-2089.
13. W. Li and X.-F. Wu, *The Journal of Organic Chemistry*, 2014, **79**, 10410-10416.
14. O. René, D. Lapointe and K. Fagnou, *Organic Letters*, 2009, **11**, 4560-4563.
15. C. M. Le, P. J. C. Menzies, D. A. Petrone and M. Lautens, *Angewandte Chemie International Edition*, 2015, **54**, 254-257.
16. X.-L. Zhang, W.-H. Zhang and H.-X. O. Yang, *Asia-Pacific Journal of Chemical Engineering*, 2009, **4**, 821-825.
17. G. Revol, T. McCallum, M. Morin, F. Gagosz and L. Barriault, *Angewandte Chemie International Edition*, 2013, **52**, 13342-13345.
18. K.-B. Wang, R.-Q. Ran, S.-D. Xiu and C.-Y. Li, *Organic Letters*, 2013, **15**, 2374-2377.

NMR Spectra

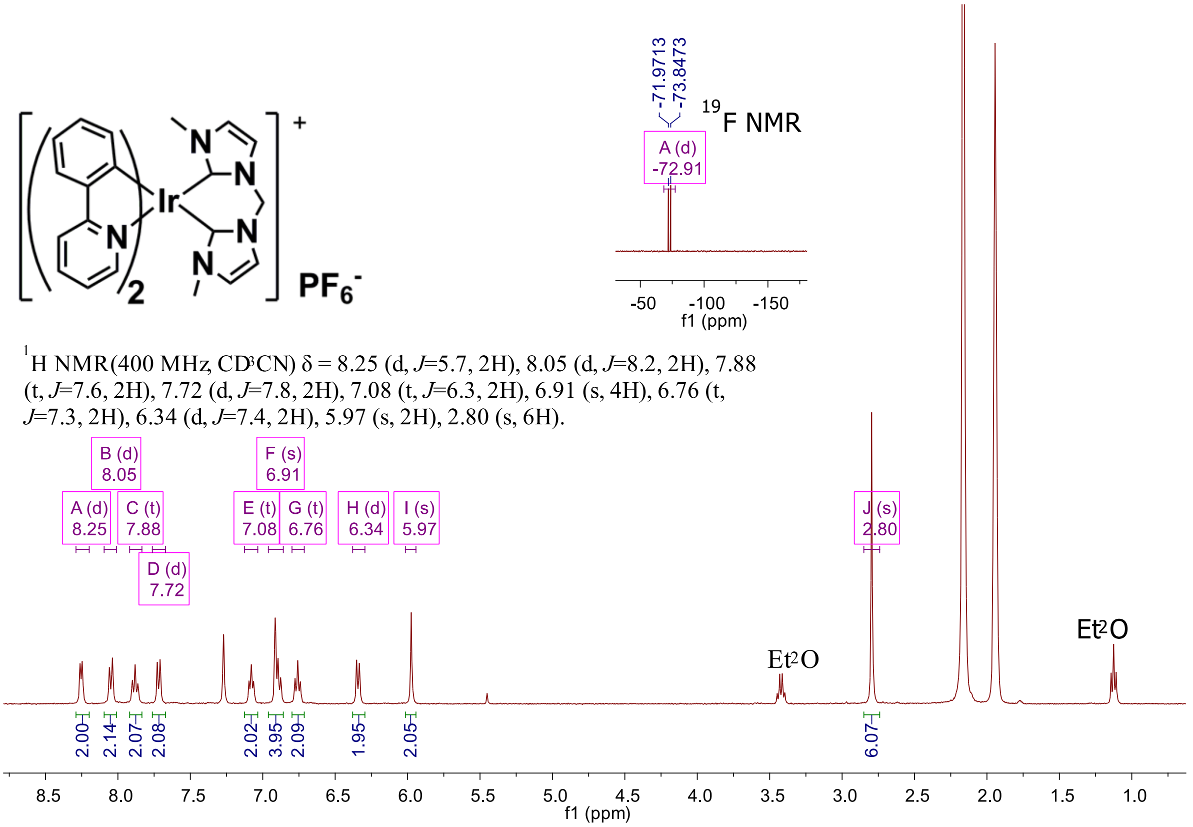
PF6-
19F NMR
A (d)
-72.91
-71.9713
-73.8473
f1 (ppm)
1H NMR(400 MHz, CD3CN) δ = 8.25 (d, J=5.7, 2H), 8.05 (d, J=8.2, 2H), 7.88 (t, J=7.6, 2H), 7.72 (d, J=7.8, 2H), 7.08 (t, J=6.3, 2H), 6.91 (s, 4H), 6.76 (t, J=7.3, 2H), 6.34 (d, J=7.4, 2H), 5.97 (s, 2H), 2.80 (s, 6H).
Et2O
Et2O
f1 (ppm)

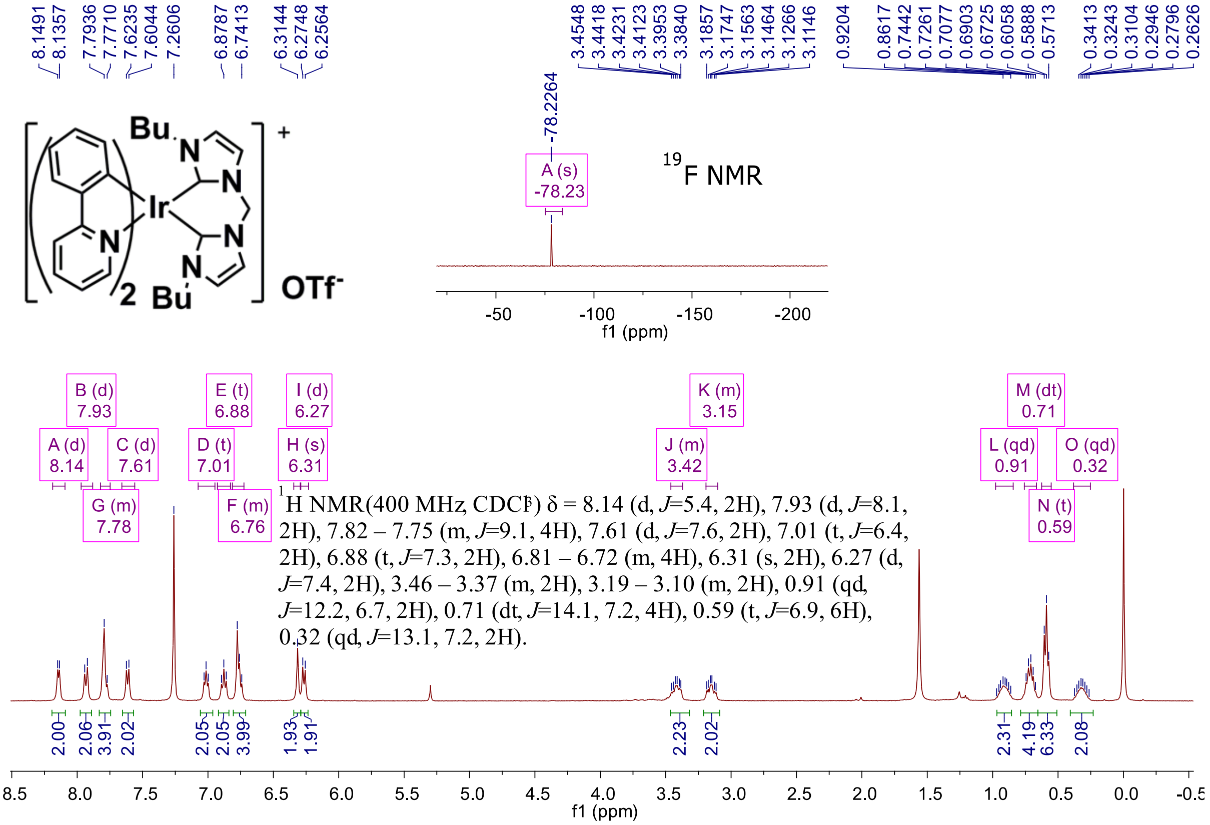
OTf-
19F NMR
A (s)
-78.23
-78.2264
f1 (ppm)
1H NMR(400 MHz, CDCl3) δ = 8.14 (d, J=5.4, 2H), 7.93 (d, J=8.1, 2H), 7.82 – 7.75 (m, J=9.1, 4H), 7.61 (d, J=7.6, 2H), 7.01 (t, J=6.4, 2H), 6.88 (t, J=7.3, 2H), 6.81 – 6.72 (m, 4H), 6.31 (s, 2H), 6.27 (d, J=7.4, 2H), 3.46 – 3.37 (m, 2H), 3.19 – 3.10 (m, 2H), 0.91 (qd, J=12.2, 6.7, 2H), 0.71 (dt, J=14.1, 7.2, 4H), 0.59 (t, J=6.9, 6H), 0.32 (qd, J=13.1, 7.2, 2H).
f1 (ppm)

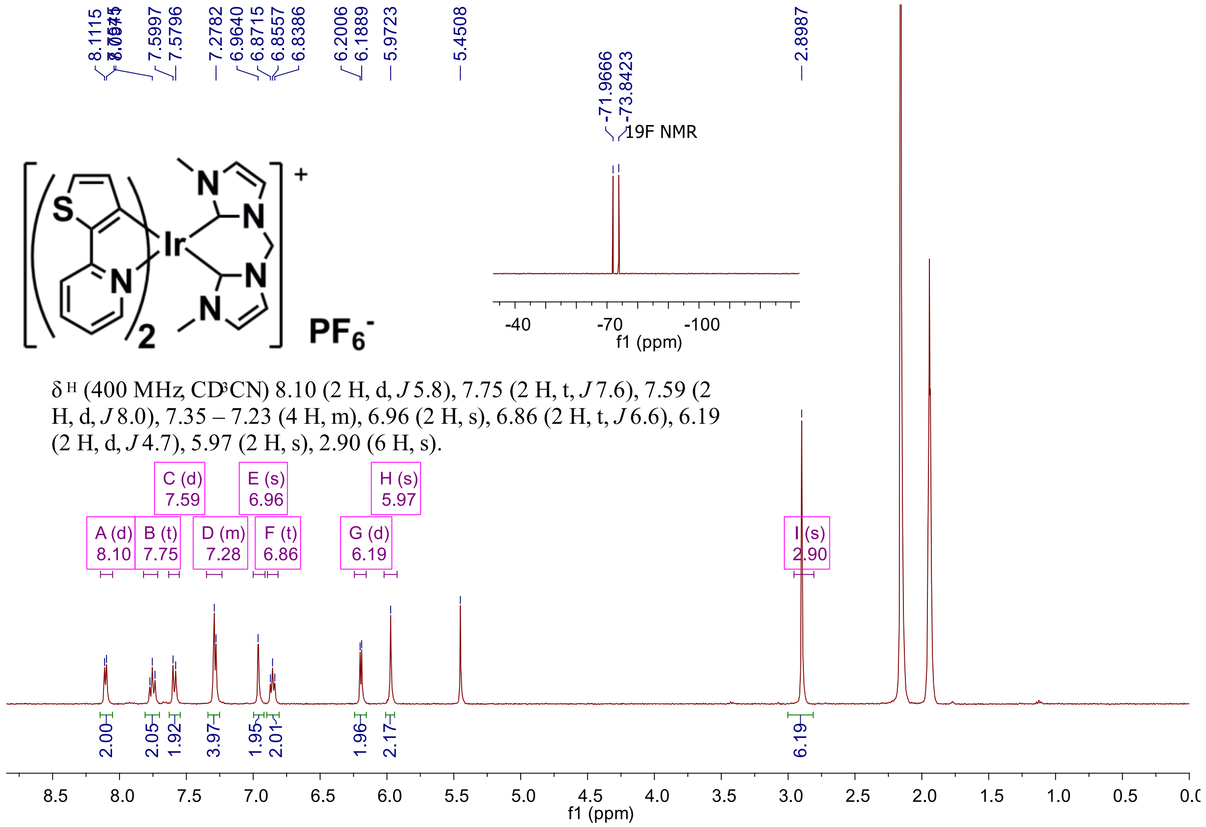
19F NMR
PF6-
f1 (ppm)
δH (400 MHz, CD3CN) 8.10 (2 H, d, J 5.8), 7.75 (2 H, t, J 7.6), 7.59 (2 H, d, J 8.0), 7.35 – 7.23 (4 H, m), 6.96 (2 H, s), 6.86 (2 H, t, J 6.6), 6.19 (2 H, d, J 4.7), 5.97 (2 H, s), 2.90 (6 H, s).

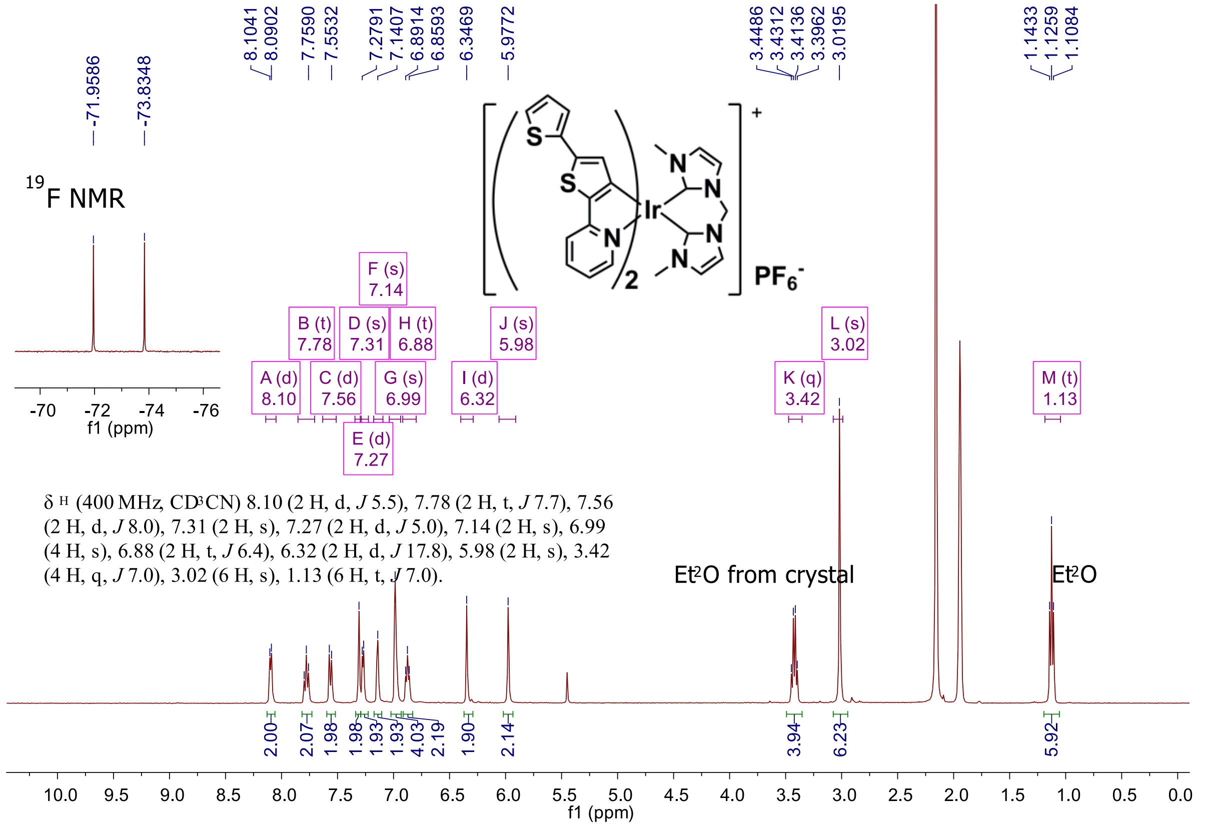
19F NMR
PF6-
f1 (ppm)
δH (400 MHz, CD3CN) 8.10 (2 H, d, J 5.5), 7.78 (2 H, t, J 7.7), 7.56 (2 H, d, J 8.0), 7.31 (2 H, s), 7.27 (2 H, d, J 5.0), 7.14 (2 H, s), 6.99 (4 H, s), 6.88 (2 H, t, J 6.4), 6.32 (2 H, d, J 17.8), 5.98 (2 H, s), 3.42 (4 H, q, J 7.0), 3.02 (6 H, s), 1.13 (6 H, t, J 7.0).
Et2O from crystal
Et2O

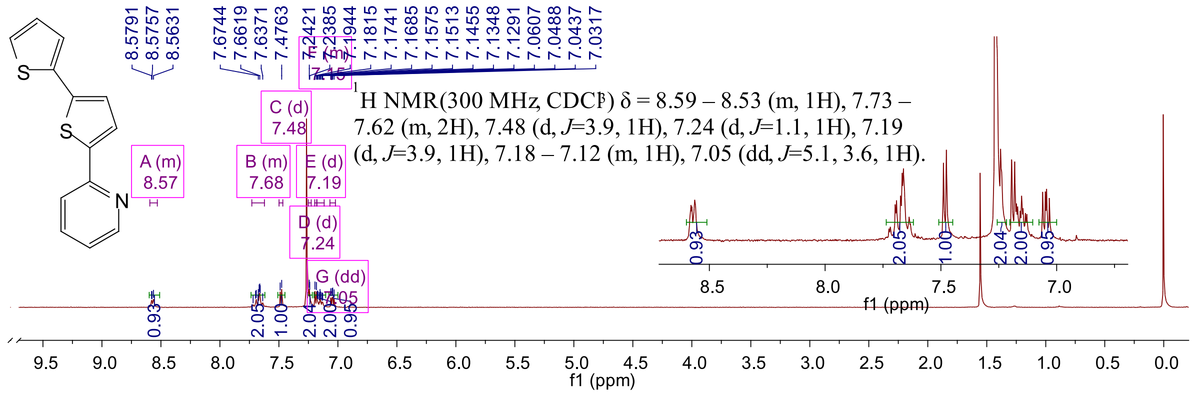

^{1}H NMR(300 MHz, $CDCl_3$) δ = 8.59 – 8.53 (m, 1H), 7.73 – 7.62 (m, 2H), 7.48 (d, *J*=3.9, 1H), 7.24 (d, *J*=1.1, 1H), 7.19 (d, *J*=3.9, 1H), 7.18 – 7.12 (m, 1H), 7.05 (dd, *J*=5.1, 3.6, 1H).

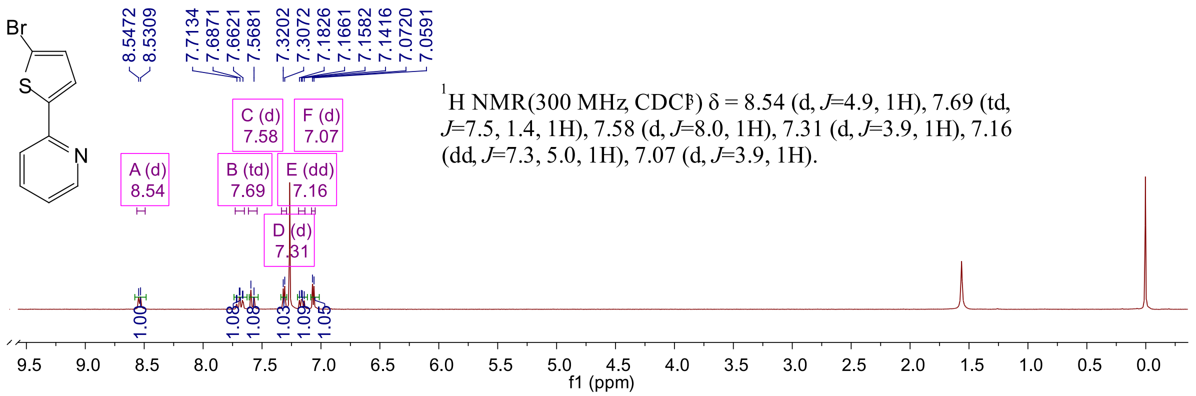

^{1}H NMR(300 MHz, $CDCl_3$) δ = 8.54 (d, *J*=4.9, 1H), 7.69 (td, *J*=7.5, 1.4, 1H), 7.58 (d, *J*=8.0, 1H), 7.31 (d, *J*=3.9, 1H), 7.16 (dd, *J*=7.3, 5.0, 1H), 7.07 (d, *J*=3.9, 1H).

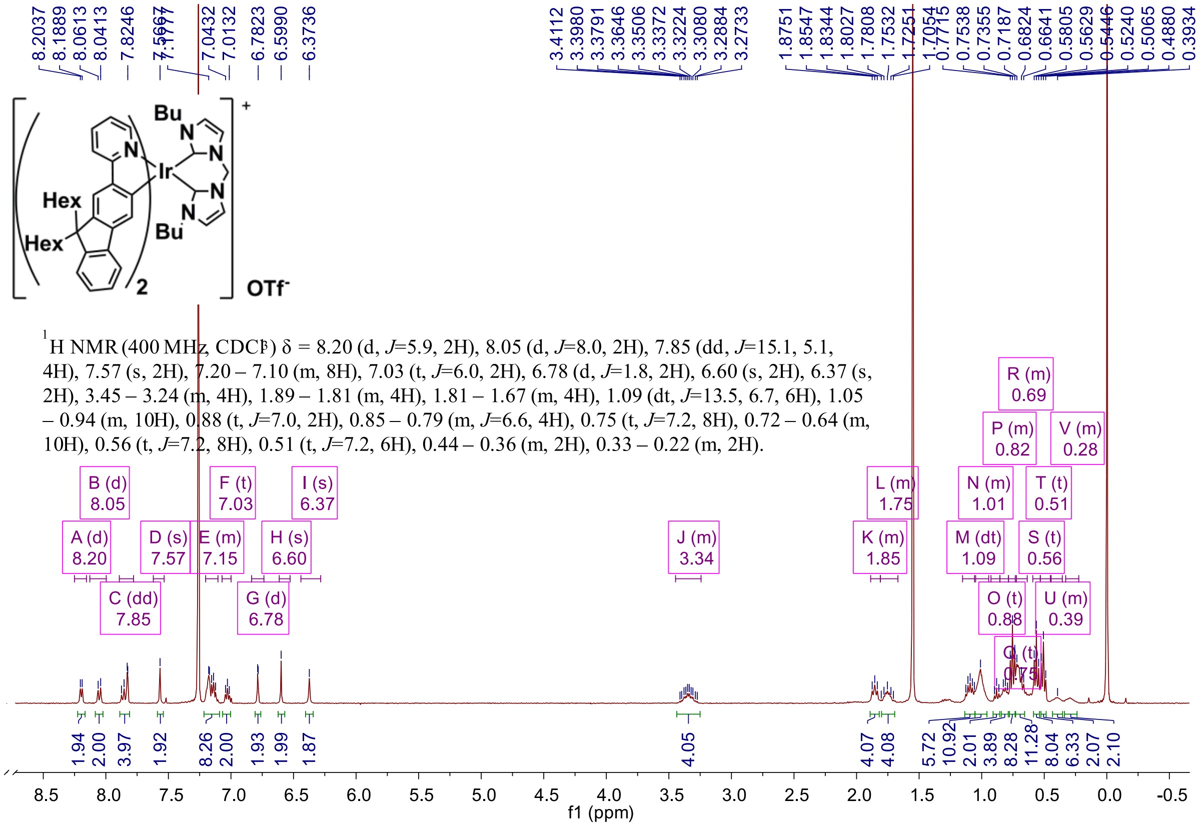

^{1}H NMR (400 MHz, $CDCl_3$) δ = 8.20 (d, *J*=5.9, 2H), 8.05 (d, *J*=8.0, 2H), 7.85 (dd, *J*=15.1, 5.1, 4H), 7.57 (s, 2H), 7.20 – 7.10 (m, 8H), 7.03 (t, *J*=6.0, 2H), 6.78 (d, *J*=1.8, 2H), 6.60 (s, 2H), 6.37 (s, 2H), 3.45 – 3.24 (m, 4H), 1.89 – 1.81 (m, 4H), 1.81 – 1.67 (m, 4H), 1.09 (dt, *J*=13.5, 6.7, 6H), 1.05 – 0.94 (m, 10H), 0.88 (t, *J*=7.0, 2H), 0.85 – 0.79 (m, *J*=6.6, 4H), 0.75 (t, *J*=7.2, 8H), 0.72 – 0.64 (m, 10H), 0.56 (t, *J*=7.2, 8H), 0.51 (t, *J*=7.2, 6H), 0.44 – 0.36 (m, 2H), 0.33 – 0.22 (m, 2H).

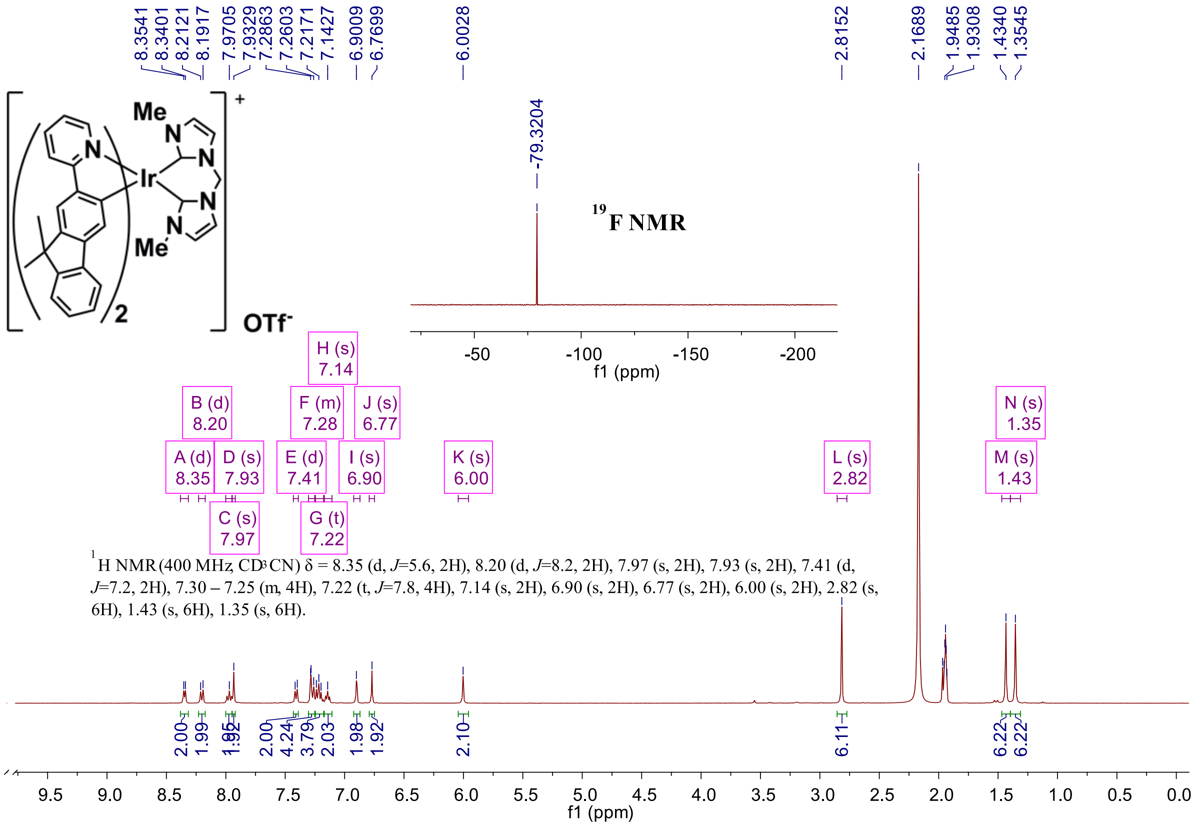
Me
Ir
N
Me
2
+
OTf-
19F NMR
f1 (ppm)
1H NMR (400 MHz, CD3CN) δ = 8.35 (d, J=5.6, 2H), 8.20 (d, J=8.2, 2H), 7.97 (s, 2H), 7.93 (s, 2H), 7.41 (d, J=7.2, 2H), 7.30 – 7.25 (m, 4H), 7.22 (t, J=7.8, 4H), 7.14 (s, 2H), 6.90 (s, 2H), 6.77 (s, 2H), 6.00 (s, 2H), 2.82 (s, 6H), 1.43 (s, 6H), 1.35 (s, 6H).
f1 (ppm)

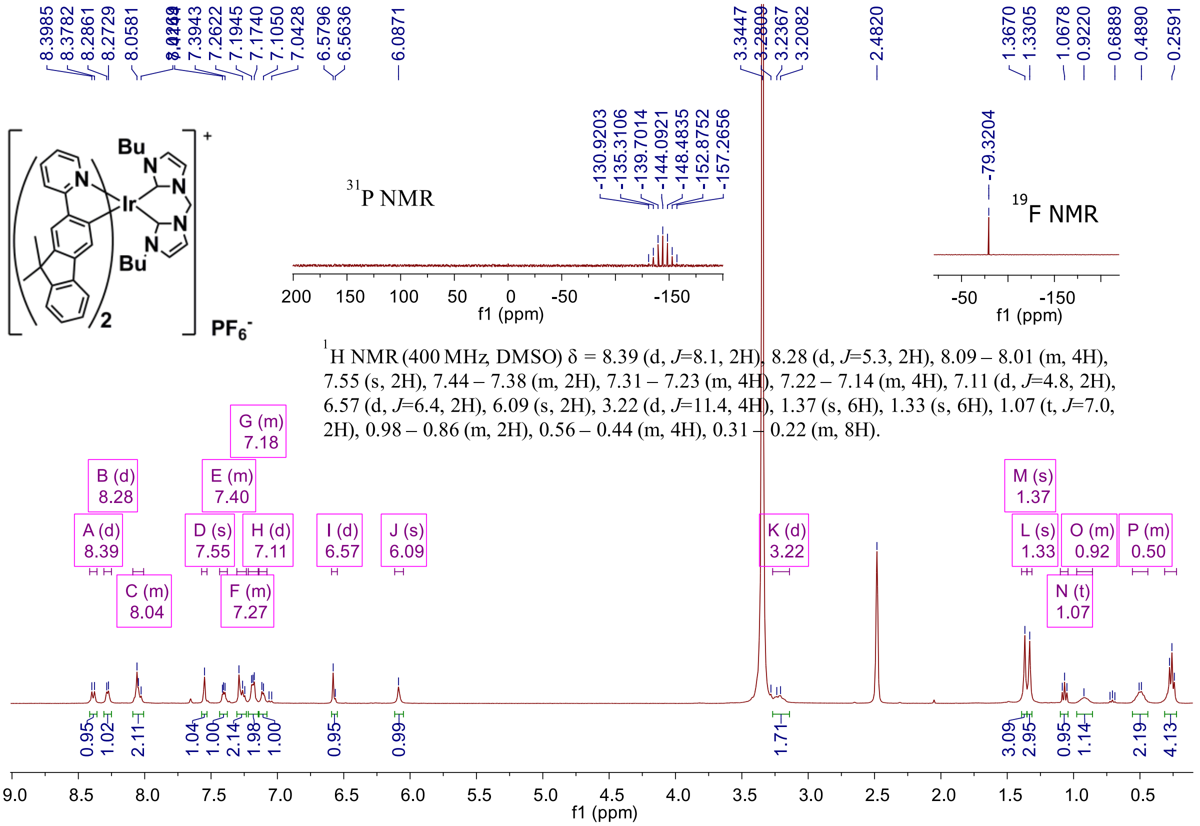
Bu
Ir
N
Bu
2
+
PF6-
31P NMR
f1 (ppm)
19F NMR
f1 (ppm)
1H NMR (400 MHz, DMSO) δ = 8.39 (d, J=8.1, 2H), 8.28 (d, J=5.3, 2H), 8.09 – 8.01 (m, 4H), 7.55 (s, 2H), 7.44 – 7.38 (m, 2H), 7.31 – 7.23 (m, 4H), 7.22 – 7.14 (m, 4H), 7.11 (d, J=4.8, 2H), 6.57 (d, J=6.4, 2H), 6.09 (s, 2H), 3.22 (d, J=11.4, 4H), 1.37 (s, 6H), 1.33 (s, 6H), 1.07 (t, J=7.0, 2H), 0.98 – 0.86 (m, 2H), 0.56 – 0.44 (m, 4H), 0.31 – 0.22 (m, 8H).
f1 (ppm)

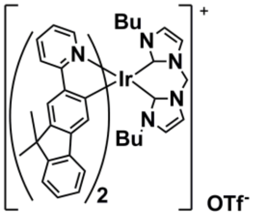

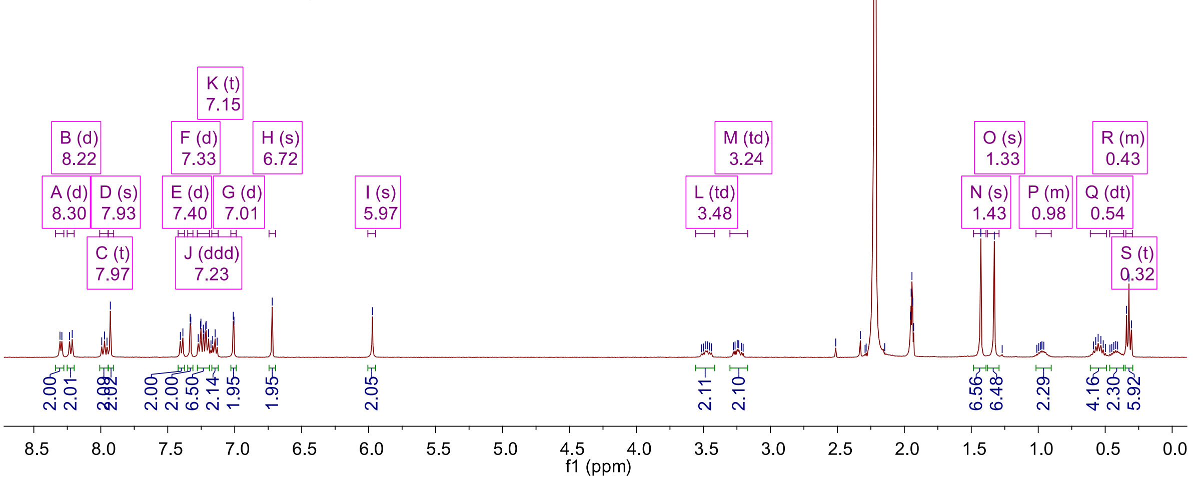

^{1}H NMR (400 MHz, CD_3CN) δ = 8.30 (d, *J*=5.7, 2H), 8.22 (d, *J*=8.1, 2H), 7.97 (t, *J*=7.8, 2H), 7.93 (s, 2H), 7.40 (d, *J*=7.4, 2H), 7.33 (d, *J*=1.9, 2H), 7.23 (ddd, *J*=13.9, 8.2, 7.1, 6H), 7.15 (t, *J*=6.6, 2H), 7.01 (d, *J*=1.9, 2H), 6.72 (s, 2H), 5.97 (s, 2H), 3.48 (td, *J*=12.5, 5.3, 2H), 3.24 (td, *J*=12.5, 5.3, 2H), 1.43 (s, 6H), 1.33 (s, 6H), 1.02 – 0.90 (m, 2H), 0.54 (dt, *J*=13.4, 6.4, 4H), 0.47 – 0.36 (m, 2H), 0.32 (t, *J*=7.2, 6H).

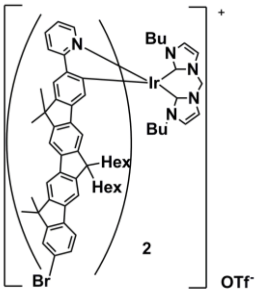

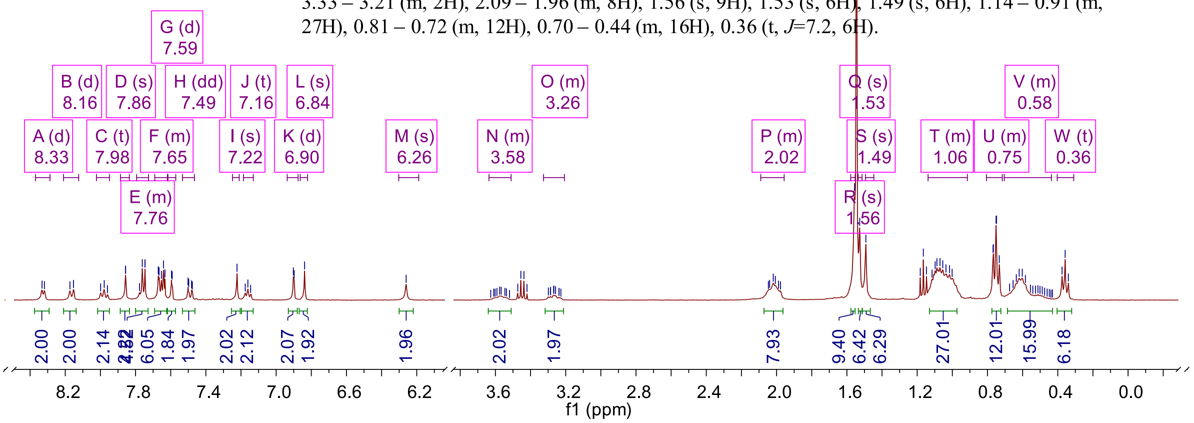

^{1}H NMR (400 MHz, CD_3OD) δ = 8.33 (d, *J*=5.3, 2H), 8.16 (d, *J*=8.0, 2H), 7.98 (t, *J*=7.7, 2H), 7.86 (s, 2H), 7.79 – 7.73 (m, 4H), 7.69 – 7.62 (m, 6H), 7.59 (d, *J*=1.6, 2H), 7.49 (dd, *J*=8.1, 1.7, 2H), 7.22 (s, 2H), 7.16 (t, *J*=6.1, 2H), 6.90 (d, *J*=1.8, 2H), 6.84 (s, 2H), 6.26 (s, 2H), 3.64 – 3.51 (m, 2H), 3.33 – 3.21 (m, 2H), 2.09 – 1.96 (m, 8H), 1.56 (s, 9H), 1.53 (s, 6H), 1.49 (s, 6H), 1.14 – 0.91 (m, 27H), 0.81 – 0.72 (m, 12H), 0.70 – 0.44 (m, 16H), 0.36 (t, *J*=7.2, 6H).

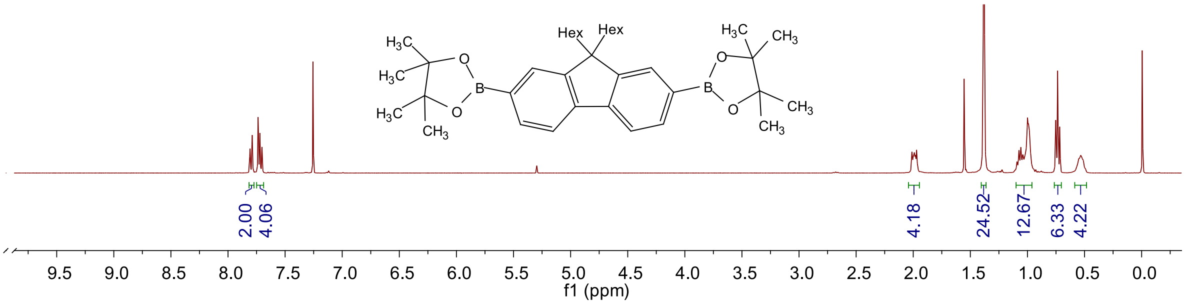

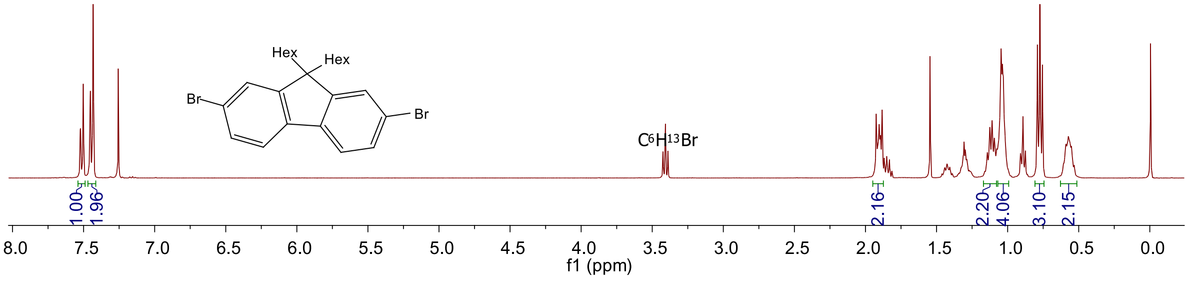

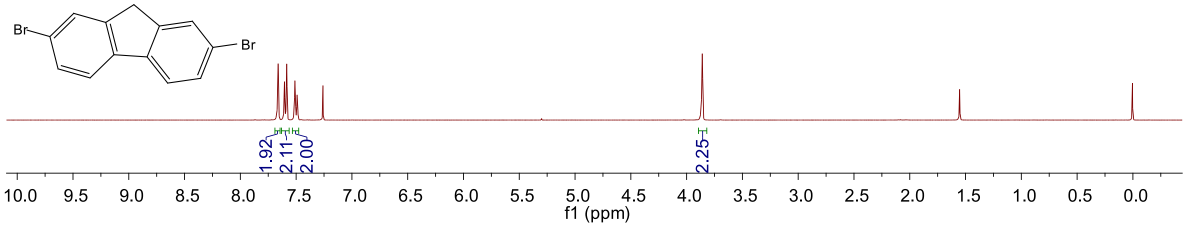

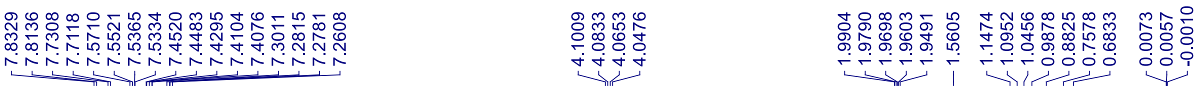

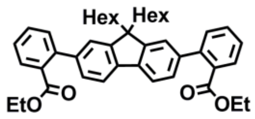

^{1}H NMR (400 MHz, CDCl$_3$) δ = 7.82 (d, *J*=7.7, 2H), 7.72 (d, *J*=7.6, 2H), 7.55 (t, *J*=4.4, 2H), 7.46 – 7.40 (m, 4H), 7.32 – 7.27 (m, 4H), 4.07 (q, *J*=7.1, 4H), 2.02 – 1.92 (m, 4H), 1.17 – 0.95 (m, 18H), 0.80 – 0.67 (m, 10H).

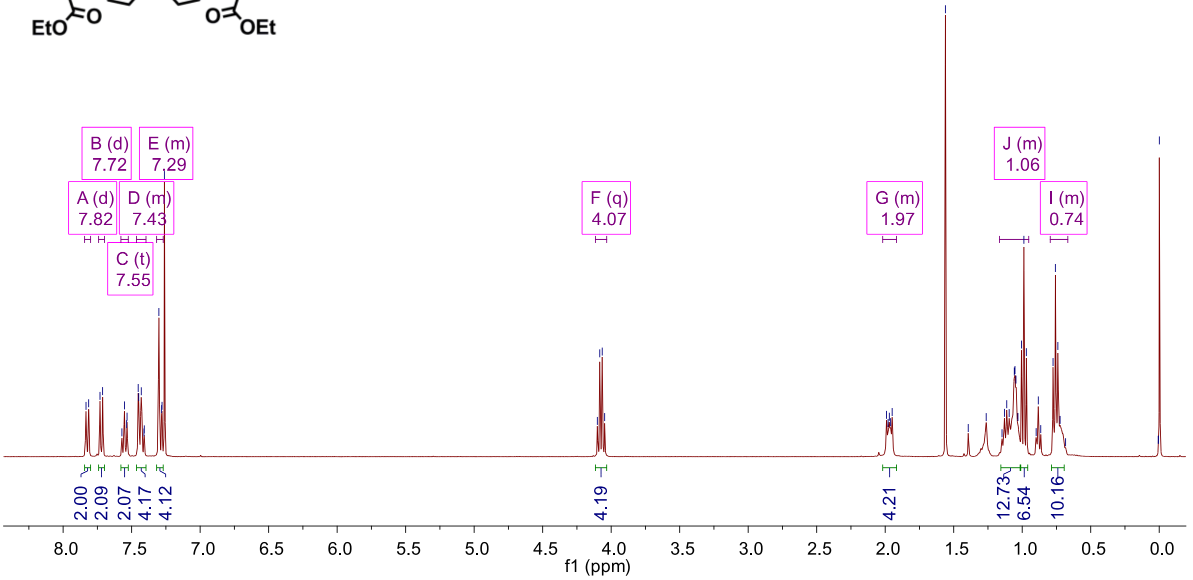

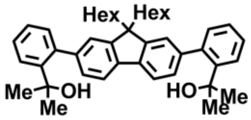

^{1}H NMR (400 MHz, $CDCl_3$) δ = 7.74 (dd, J=8.4, 1.6, 4H), 7.39 (td, J=7.3, 1.4, 2H), 7.32 – 7.27 (m, 6H), 7.16 (dd, J=7.5, 1.2, 2H), 1.99 – 1.92 (m, 4H), 1.84 (s, 2H), 1.47 (s, 12H), 1.16 – 1.08 (m, 4H), 1.07 – 0.99 (m, 8H), 0.77 (t, J=7.1, 6H), 0.73 – 0.61 (m, 4H).

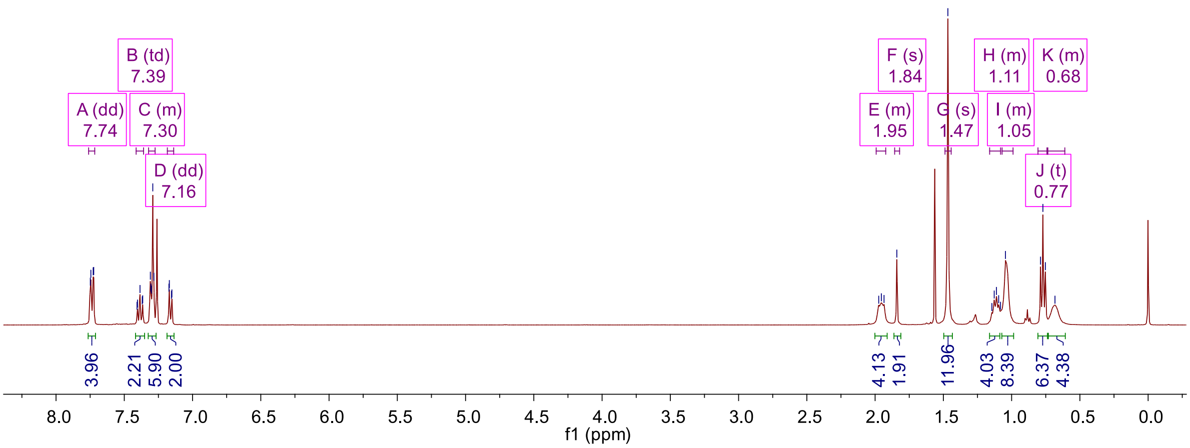

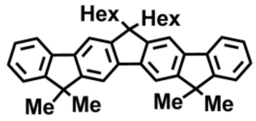

^{1}H NMR (400 MHz, $CDCl_3$) δ = 7.82 – 7.75 (m, 4H), 7.65 (s, 2H), 7.46 (d, J=7.3, 2H), 7.37 (td, J=7.3, 1.1, 2H), 7.31 (td, J=7.3, 1.0, 2H), 2.11 – 2.01 (m, 4H), 1.58 (s, 12H), 1.15 – 0.97 (m, 12H), 0.77 – 0.66 (m, 10H).

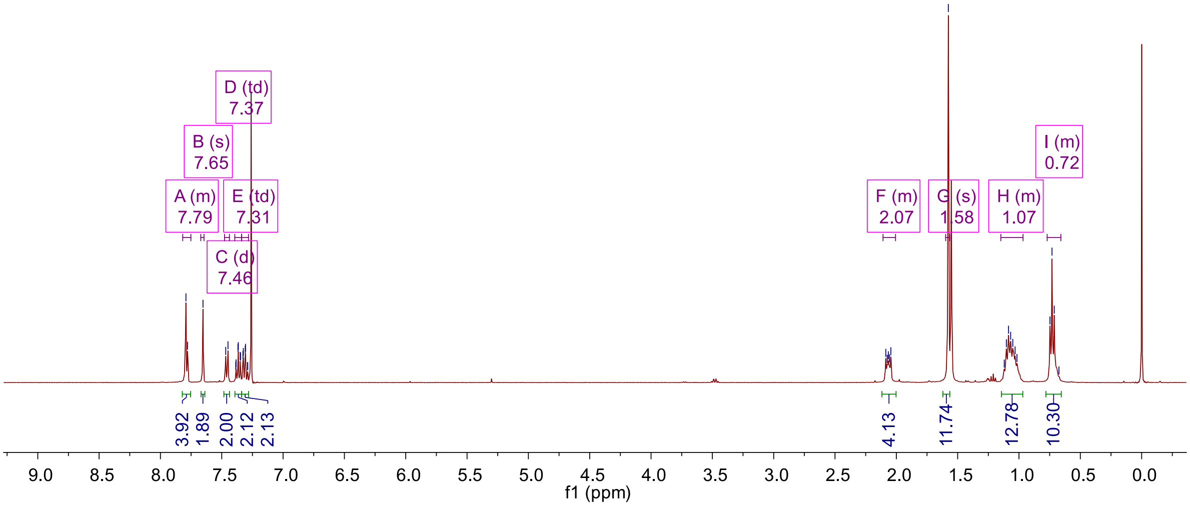

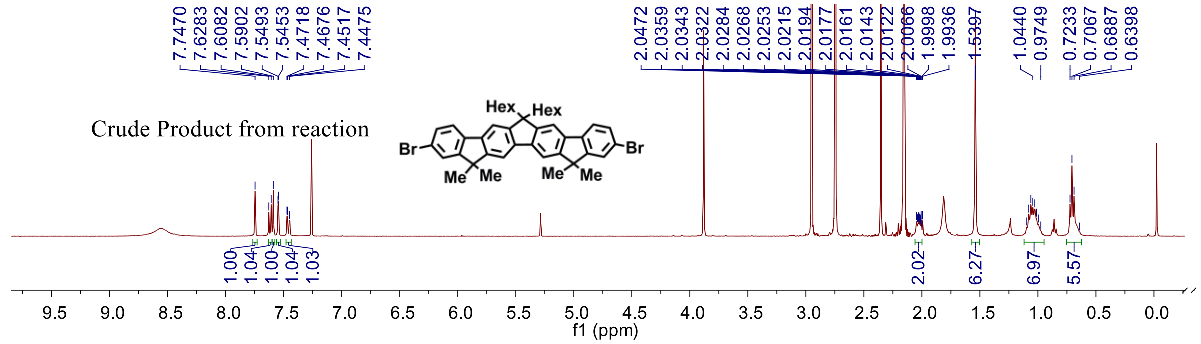
Crude Product from reaction
Hex
Hex
Br
Br
Me
Me
Me
Me
f1 (ppm)

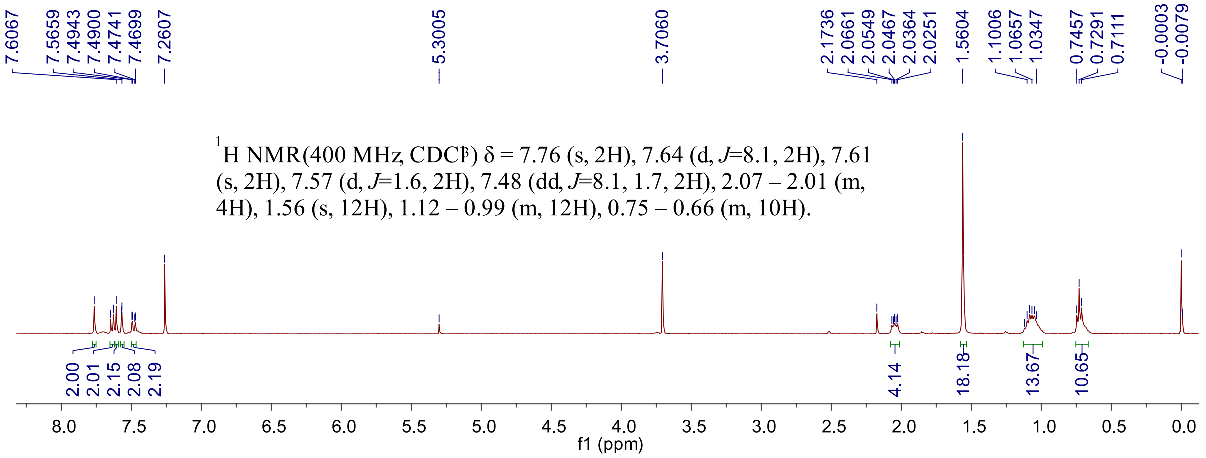
1H NMR(400 MHz, CDCl3) δ = 7.76 (s, 2H), 7.64 (d, J=8.1, 2H), 7.61 (s, 2H), 7.57 (d, J=1.6, 2H), 7.48 (dd, J=8.1, 1.7, 2H), 2.07 – 2.01 (m, 4H), 1.56 (s, 12H), 1.12 – 0.99 (m, 12H), 0.75 – 0.66 (m, 10H).
f1 (ppm)

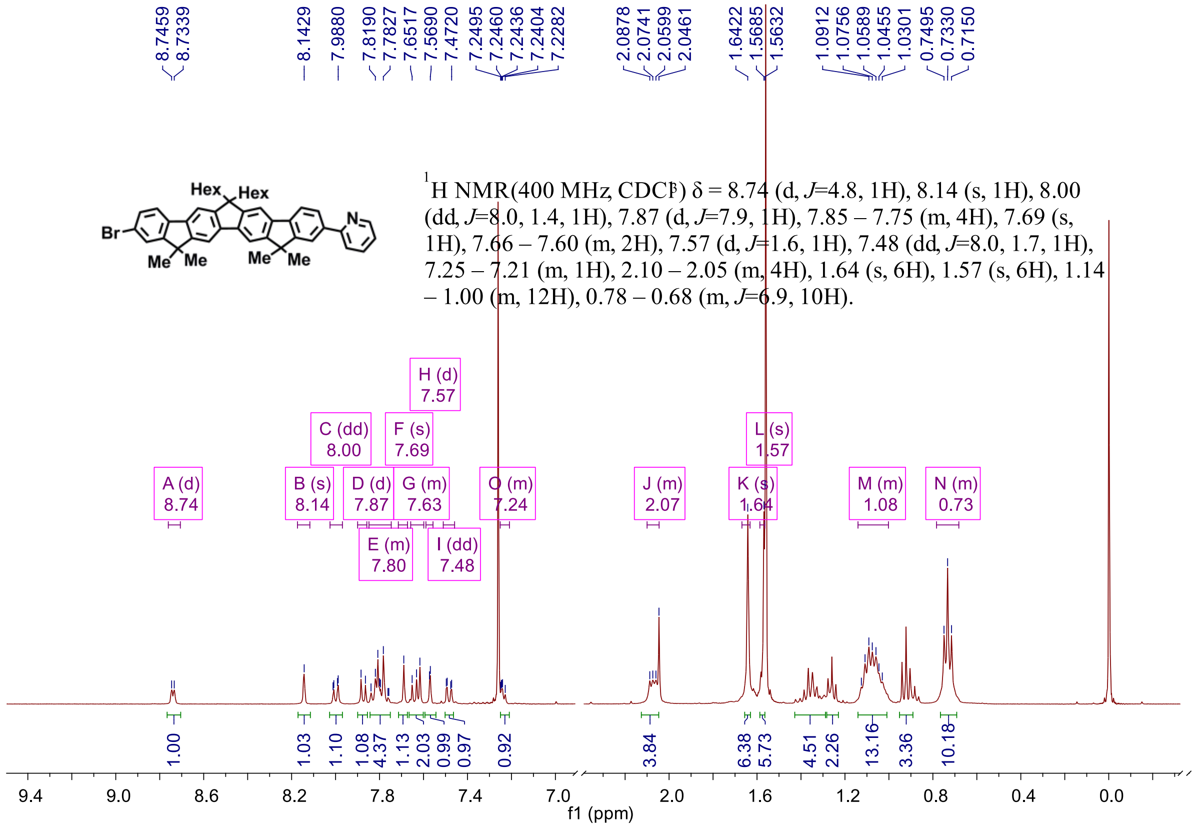
Hex
Hex
Br
N
Me
Me
Me
Me
1H NMR(400 MHz, CDCl3) δ = 8.74 (d, J=4.8, 1H), 8.14 (s, 1H), 8.00 (dd, J=8.0, 1.4, 1H), 7.87 (d, J=7.9, 1H), 7.85 – 7.75 (m, 4H), 7.69 (s, 1H), 7.66 – 7.60 (m, 2H), 7.57 (d, J=1.6, 1H), 7.48 (dd, J=8.0, 1.7, 1H), 7.25 – 7.21 (m, 1H), 2.10 – 2.05 (m, 4H), 1.64 (s, 6H), 1.57 (s, 6H), 1.14 – 1.00 (m, 12H), 0.78 – 0.68 (m, J=6.9, 10H).
f1 (ppm)

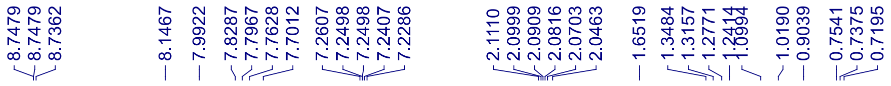

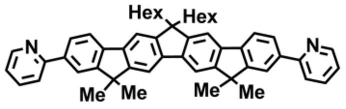

^{1}H NMR (400 MHz, CDCl$_3$) δ = 8.74 (d, *J*=4.7, 2H), 8.15 (s, 2H), 8.00 (dd, *J*=7.9, 1.4, 2H), 7.88 (d, *J*=8.0, 2H), 7.85 – 7.81 (m, 4H), 7.78 (td, *J*=7.7, 1.6, 2H), 7.70 (s, 2H), 7.26 – 7.21 (m, 2H), 2.12 – 2.06 (m, 4H), 1.65 (s, 12H), 1.15 – 1.02 (m, 12H), 0.80 – 0.69 (m, 10H).

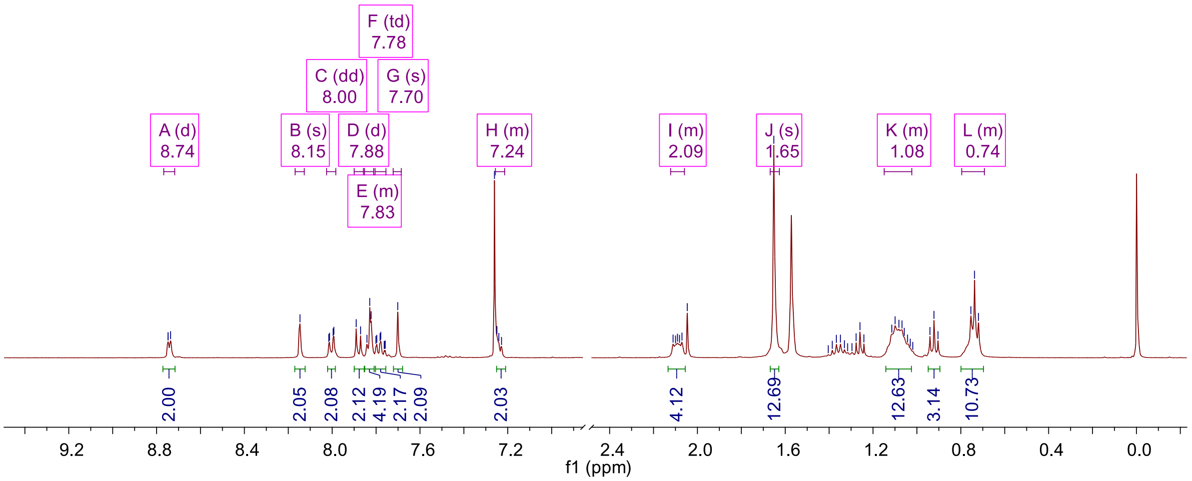

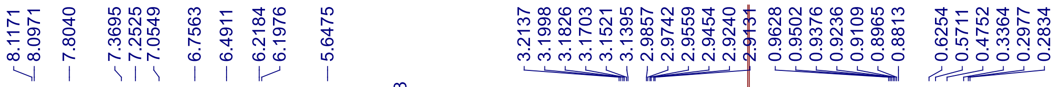

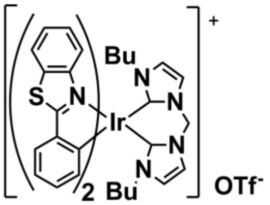

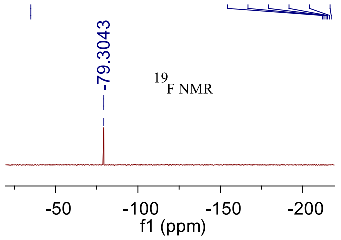

^{1}H NMR (400 MHz, CD$_3$CN) δ = 8.11 (d, *J*=8.0, 2H), 7.81 (d, *J*=7.6, 2H), 7.49 (t, *J*=7.5, 2H), 7.37 (s, 2H), 7.27 (t, *J*=7.7, 2H), 7.05 (s, 2H), 6.95 (t, *J*=7.4, 2H), 6.77 (t, *J*=7.4, 2H), 6.50 (d, *J*=7.5, 2H), 6.21 (d, *J*=8.3, 2H), 5.65 (s, 2H), 3.18 (td, *J*=12.4, 5.3, 2H), 2.95 (td, *J*=12.3, 4.2, 2H), 1.01 – 0.85 (m, 2H), 0.71 – 0.55 (m, 4H), 0.49 (t, *J*=7.0, 6H), 0.39 – 0.22 (m, 2H).

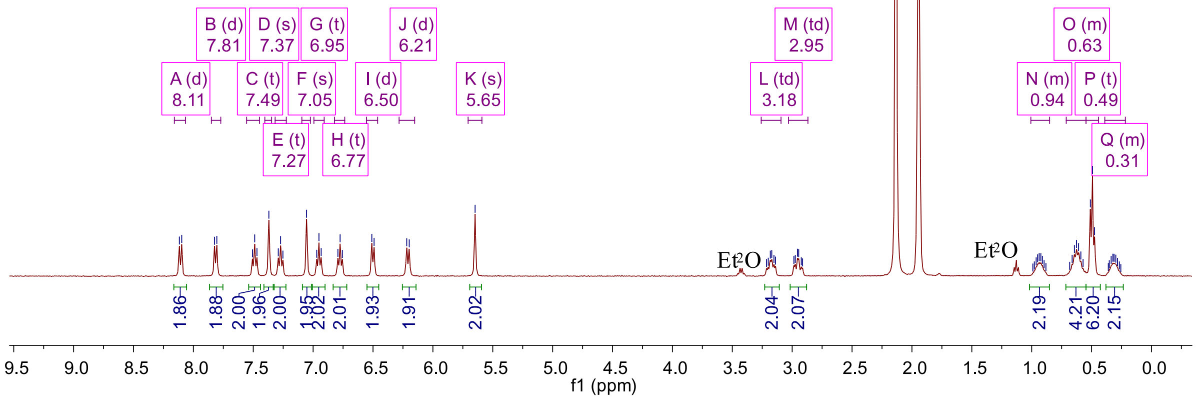

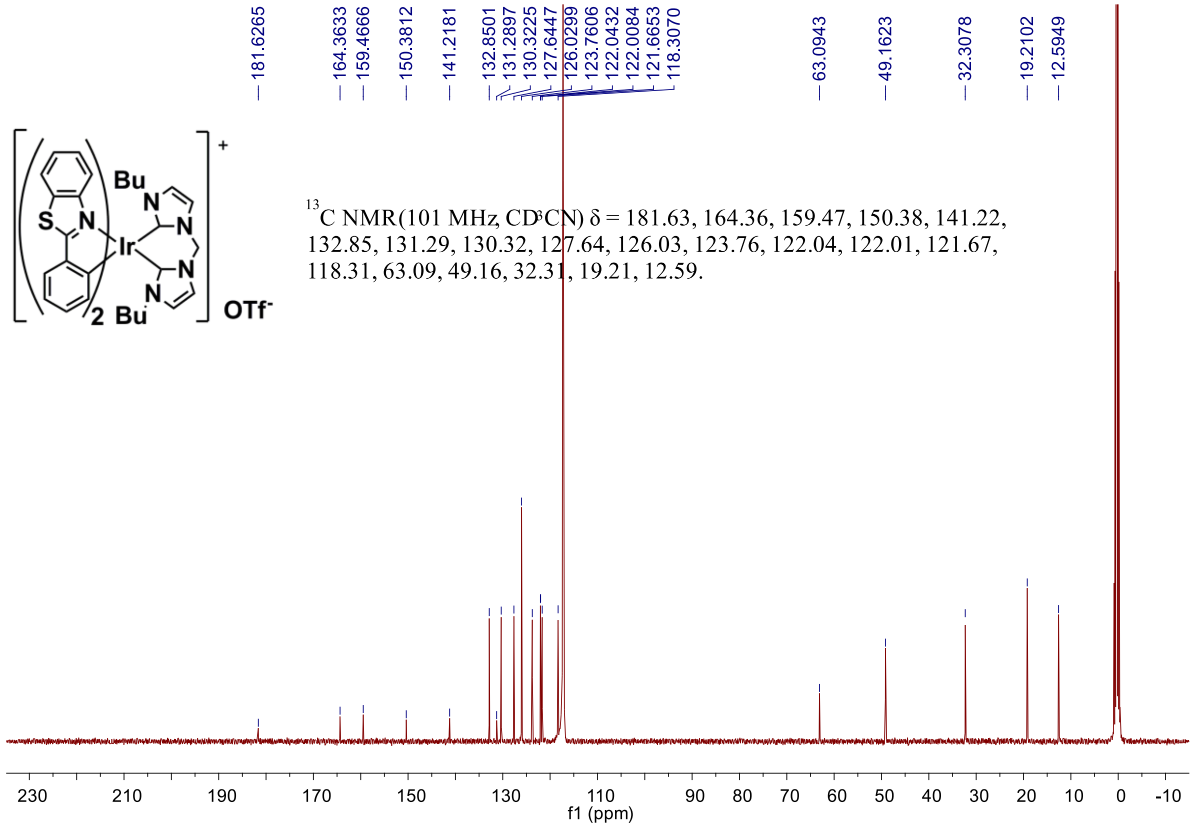

^{13}C NMR(101 MHz, CD_3CN) δ = 181.63, 164.36, 159.47, 150.38, 141.22, 132.85, 131.29, 130.32, 127.64, 126.03, 123.76, 122.04, 122.01, 121.67, 118.31, 63.09, 49.16, 32.31, 19.21, 12.59.

cis-6^{H}b

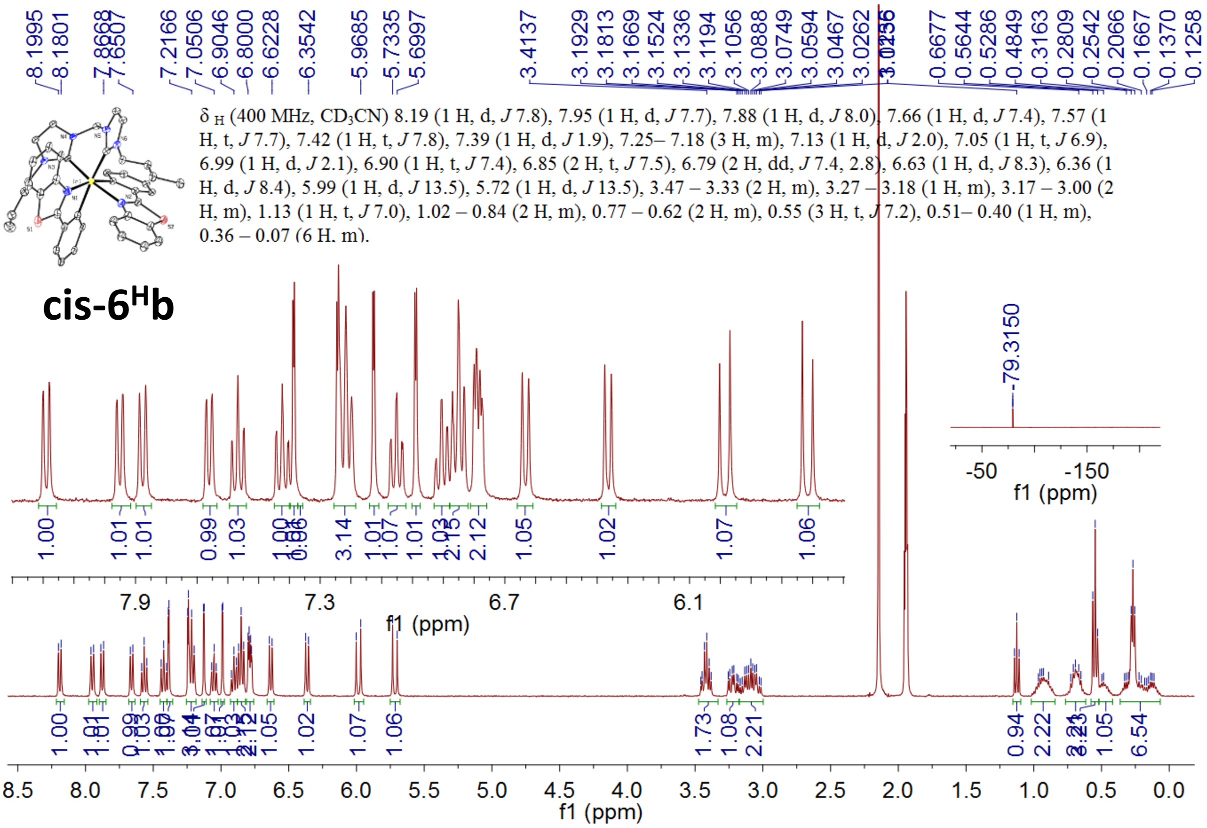

δ_H (400 MHz, CD_3CN) 8.19 (1 H, d, *J* 7.8), 7.95 (1 H, d, *J* 7.7), 7.88 (1 H, d, *J* 8.0), 7.66 (1 H, d, *J* 7.4), 7.57 (1 H, t, *J* 7.7), 7.42 (1 H, t, *J* 7.8), 7.39 (1 H, d, *J* 1.9), 7.25– 7.18 (3 H, m), 7.13 (1 H, d, *J* 2.0), 7.05 (1 H, t, *J* 6.9), 6.99 (1 H, d, *J* 2.1), 6.90 (1 H, t, *J* 7.4), 6.85 (2 H, t, *J* 7.5), 6.79 (2 H, dd, *J* 7.4, 2.8), 6.63 (1 H, d, *J* 8.3), 6.36 (1 H, d, *J* 8.4), 5.99 (1 H, d, *J* 13.5), 5.72 (1 H, d, *J* 13.5), 3.47 – 3.33 (2 H, m), 3.27 – 3.18 (1 H, m), 3.17 – 3.00 (2 H, m), 1.13 (1 H, t, *J* 7.0), 1.02 – 0.84 (2 H, m), 0.77 – 0.62 (2 H, m), 0.55 (3 H, t, *J* 7.2), 0.51– 0.40 (1 H, m), 0.36 – 0.07 (6 H, m).

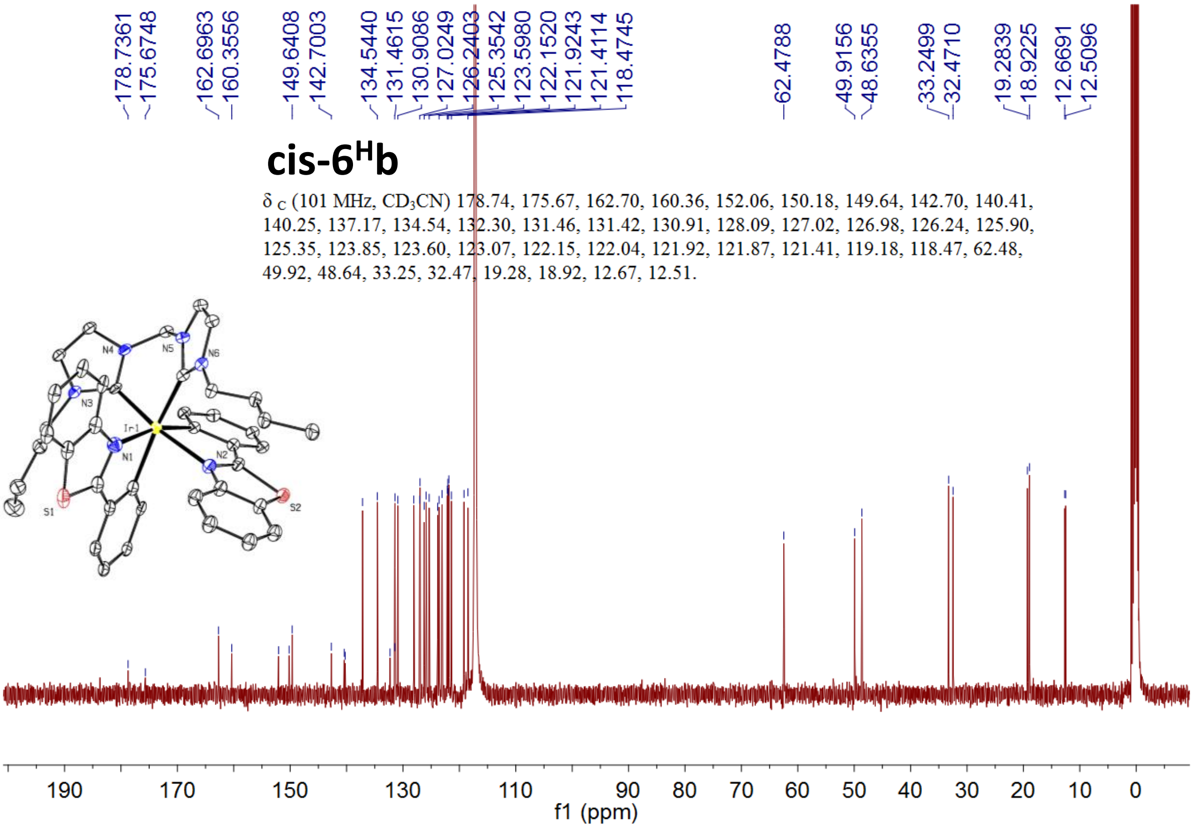

cis-6^{H}b

δ_C (101 MHz, CD_3CN) 178.74, 175.67, 162.70, 160.36, 152.06, 150.18, 149.64, 142.70, 140.41, 140.25, 137.17, 134.54, 132.30, 131.46, 131.42, 130.91, 128.09, 127.02, 126.98, 126.24, 125.90, 125.35, 123.85, 123.60, 123.07, 122.15, 122.04, 121.92, 121.87, 121.41, 119.18, 118.47, 62.48, 49.92, 48.64, 33.25, 32.47, 19.28, 18.92, 12.67, 12.51.

δ_H (400 MHz, CD^{3}CN) 8.10 (2 H, d, *J* 8.1), 7.82 (2 H, d, *J* 7.7), 7.49 (2 H, t, *J* 7.7), 7.32 (2 H, d, *J* 1.5), 7.28 (2 H, t, *J* 7.8), 6.99 – 6.91 (4 H, m), 6.76 (2 H, t, *J* 7.4), 6.54 (2 H, d, *J* 7.6), 6.30 (2 H, d, *J* 8.4), 5.68 (2 H, s), 2.62 (6 H, s).

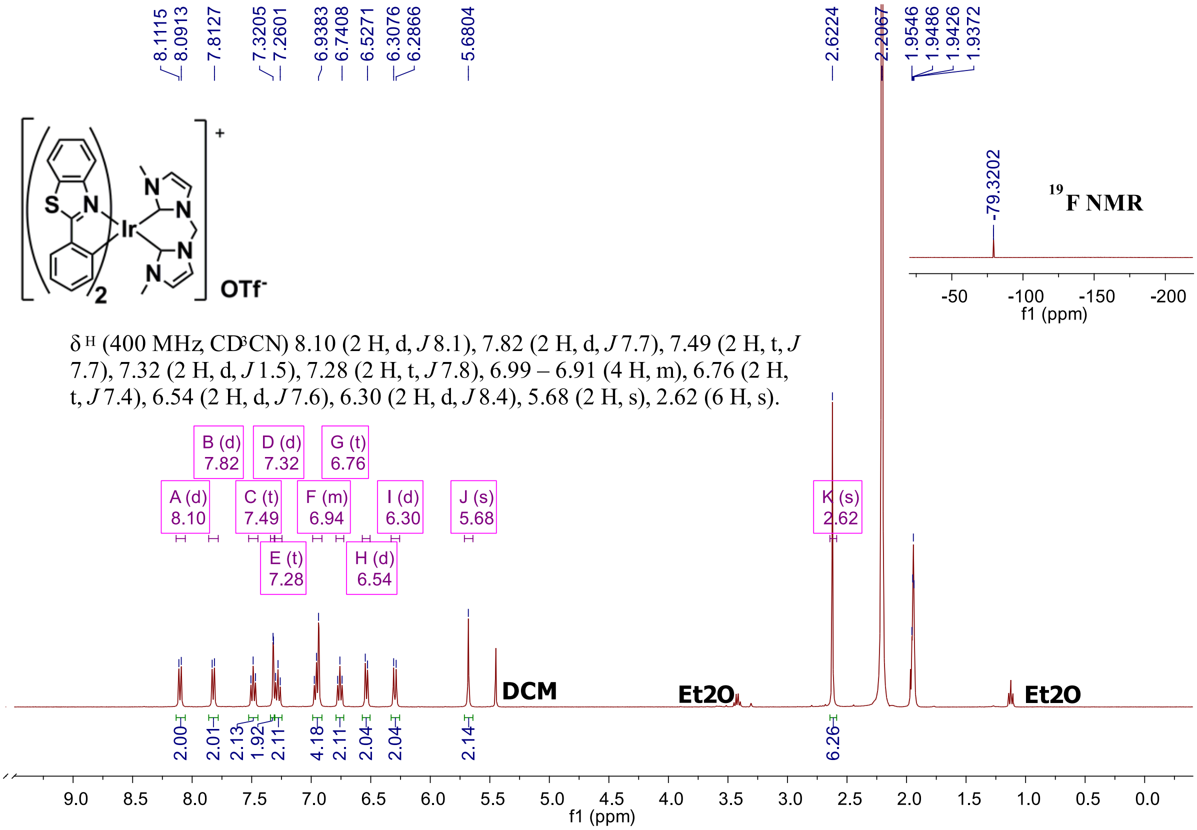

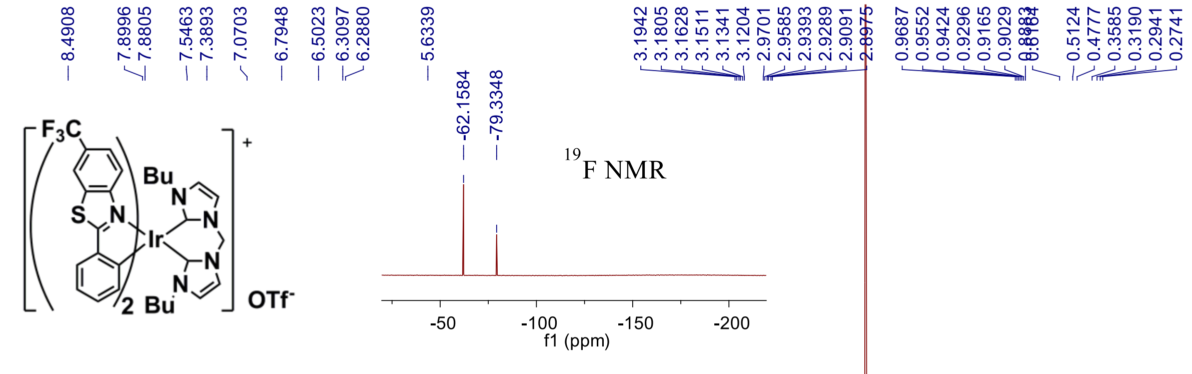

^{1}H NMR (400 MHz, DMSO) δ = 8.49 (s, 2H), 7.89 (d, J=7.6, 2H), 7.56 (d, J=8.8, 2H), 7.39 (s, 2H), 7.07 (s, 2H), 6.99 (t, J=7.4, 2H), 6.81 (t, J=7.3, 2H), 6.51 (d, J=7.5, 2H), 6.30 (d, J=8.7, 2H), 5.63 (s, 2H), 3.16 (td, J=12.4, 5.5, 2H), 2.93 (td, J=12.4, 4.6, 2H), 1.04 – 0.81 (m, 2H), 0.74 – 0.53 (m, 4H), 0.50 (t, J=6.9, 6H), 0.40 – 0.21 (m, 2H).

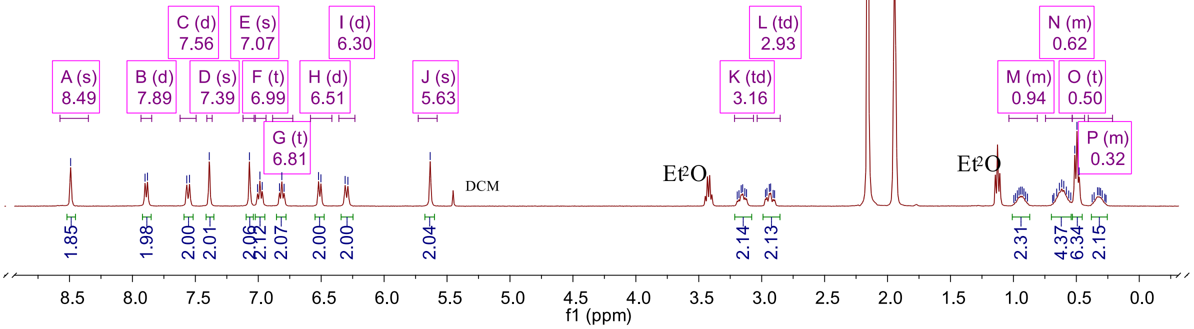

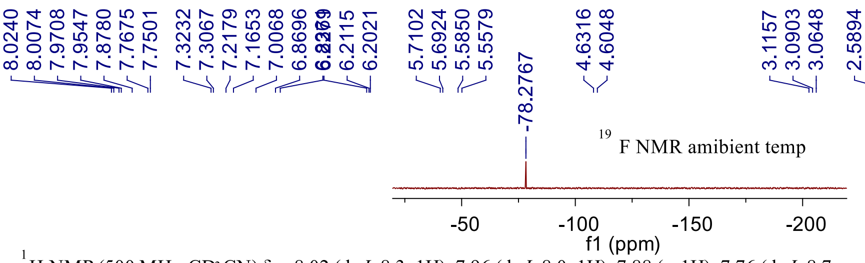

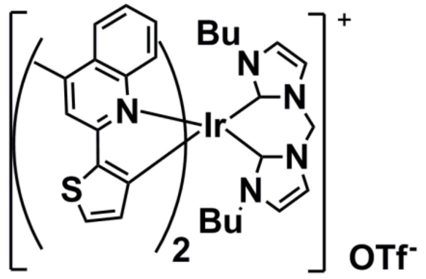

^{1}H NMR (500 MHz, CD_3CN) δ = 8.02 (d, J=8.3, 1H), 7.96 (d, J=8.0, 1H), 7.88 (s, 1H), 7.76 (d, J=8.7, 1H), 7.53 (t, J=7.5, 1H), 7.46 (s, 1H), 7.37 – 7.29 (m, 4H), 7.22 (d, J=1.7, 1H), 7.17 (d, J=1.7, 1H), 7.01 (d, J=1.7, 1H), 6.89 – 6.82 (m, 2H), 6.22 (dd, J=12.9, 4.7, 2H), 5.70 (d, J=8.9, 1H), 5.57 (d, J=13.6, 1H), 4.62 (d, J=13.4, 1H), 3.16 – 3.02 (m, 2H), 2.88 (s, 3H), 2.75 (s, 3H), 2.21 (t, J=10.9, 1H), 1.10 – 1.01 (m, 1H), 0.88 – 0.79 (m, 1H), 0.71 (t, J=7.2, 1H), 0.66 – 0.41 (m, 8H), 0.36 (t, J=7.1, 3H), 0.26 – 0.14 (m, 1H), -0.26 (s, 1H).

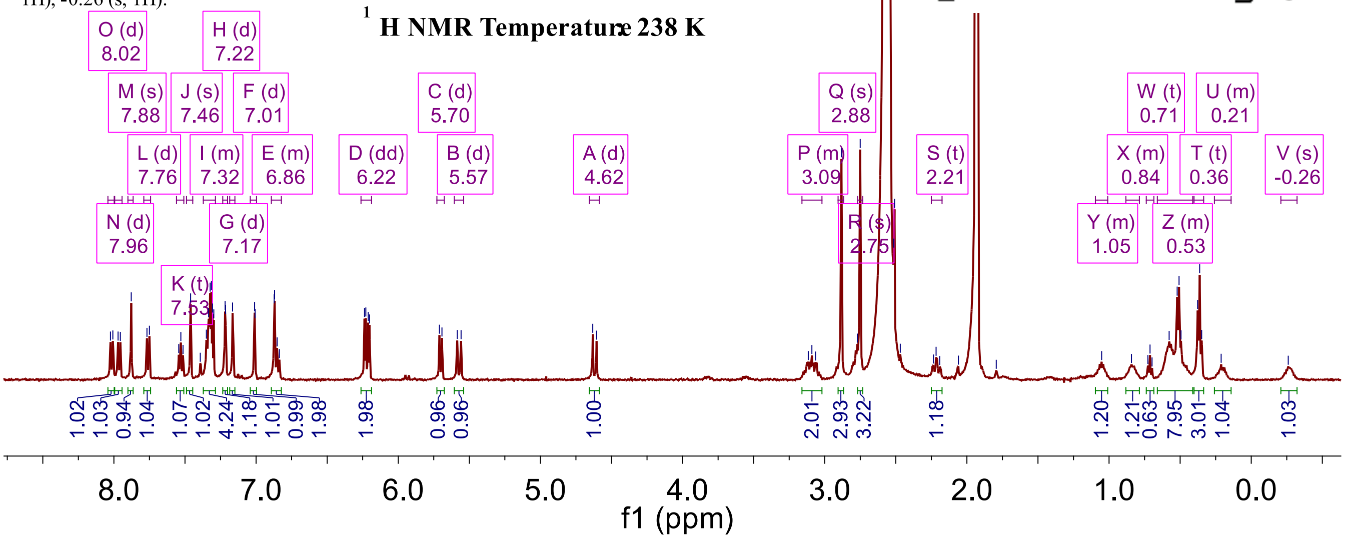

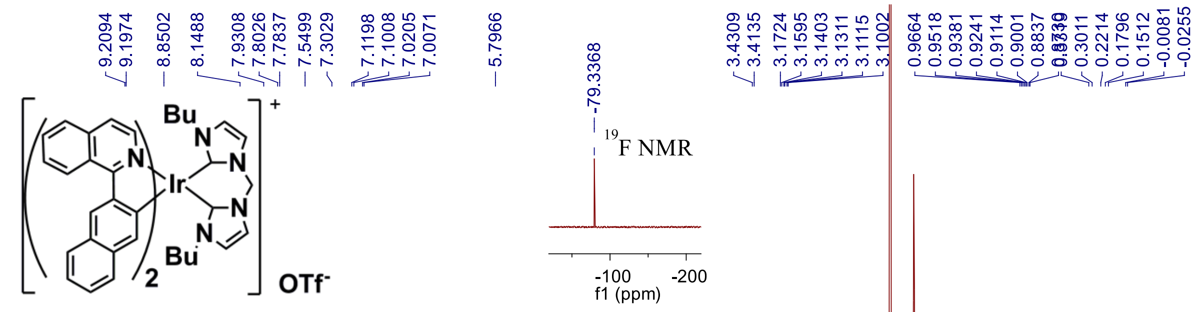

^{1}H NMR (400 MHz, CD_3CN) δ = 9.20 (d, *J*=4.8, 2H), 8.85 (s, 2H), 8.15 (t, *J*=8.0, 4H), 7.94 (d, *J*=7.7, 4H), 7.79 (d, *J*=7.6, 2H), 7.56 (d, *J*=6.4, 2H), 7.30 (s, 2H), 7.18 (dd, *J*=14.5, 7.1, 4H), 7.11 (d, *J*=7.6, 2H), 7.01 (d, *J*=5.4, 4H), 5.80 (s, 2H), 3.46 – 3.37 (m, 2H), 3.14 (td, *J*=12.6, 4.8, 2H), 1.01 – 0.82 (m, 2H), 0.40 – 0.26 (m, 2H), 0.25 – 0.10 (m, 4H), -0.03 (t, *J*=7.0, 6H).

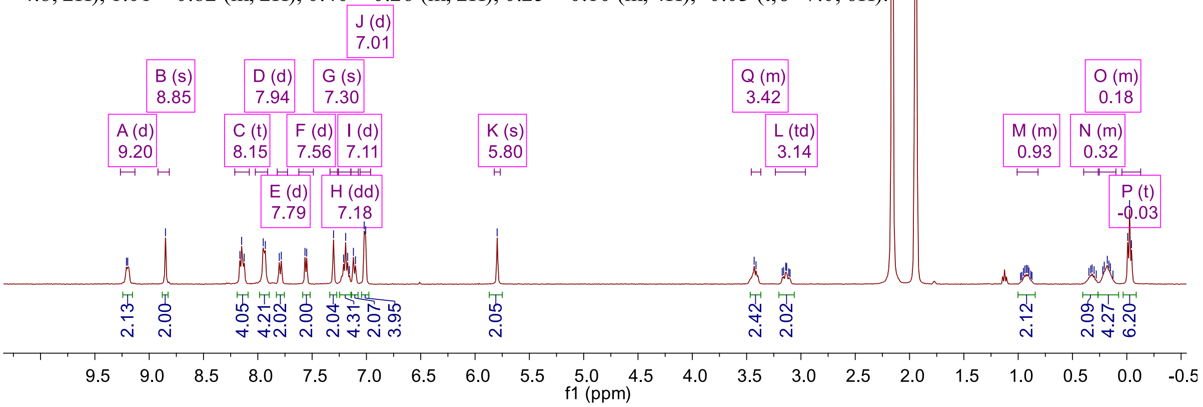

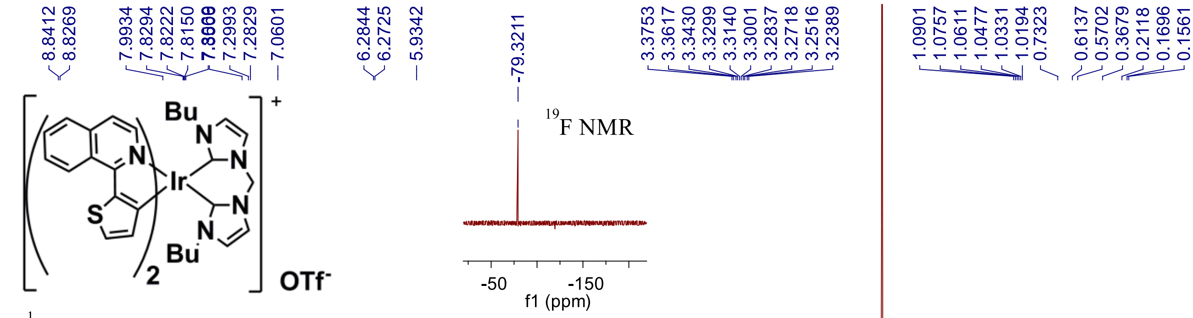

^{1}H NMR (400 MHz, CD_3CN) δ = 8.83 (d, *J*=5.7, 2H), 8.00 (d, *J*=6.2, 4H), 7.82 (dd, *J*=6.0, 3.1, 4H), 7.44 (d, *J*=4.7, 2H), 7.35 (s, 2H), 7.29 (d, *J*=6.6, 2H), 7.06 (s, 2H), 6.28 (d, *J*=4.8, 2H), 5.93 (s, 2H), 3.31 (pd, *J*=13.0, 5.3, 4H), 1.11 – 0.99 (m, 2H), 0.82 – 0.68 (m, 2H), 0.61 (ddd, *J*=17.4, 11.5, 6.1, 2H), 0.39 (t, *J*=7.3, 6H), 0.26 – 0.09 (m, 2H).

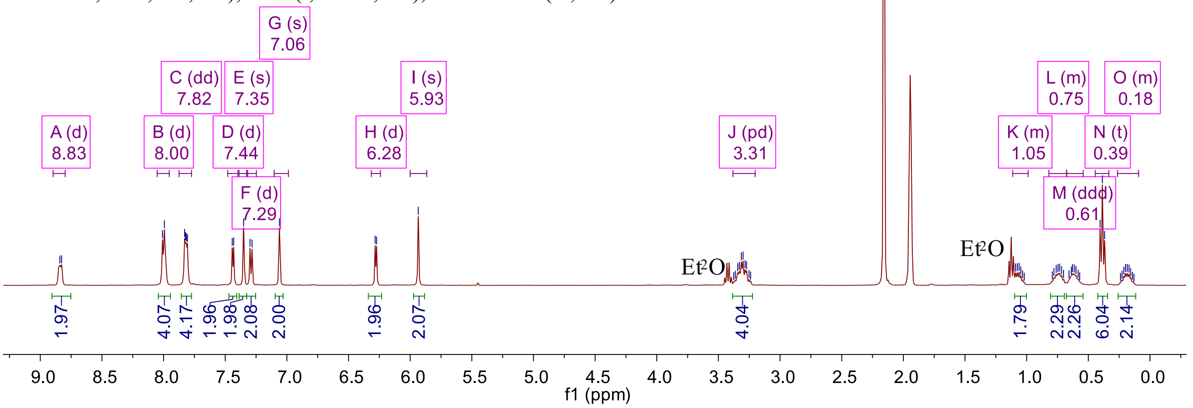

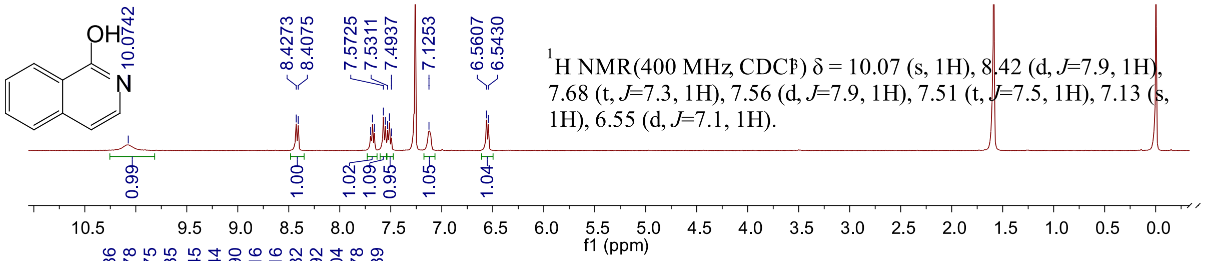

^{1}H NMR(400 MHz, $CDCl_3$) δ = 10.07 (s, 1H), 8.42 (d, *J*=7.9, 1H), 7.68 (t, *J*=7.3, 1H), 7.56 (d, *J*=7.9, 1H), 7.51 (t, *J*=7.5, 1H), 7.13 (s, 1H), 6.55 (d, *J*=7.1, 1H).

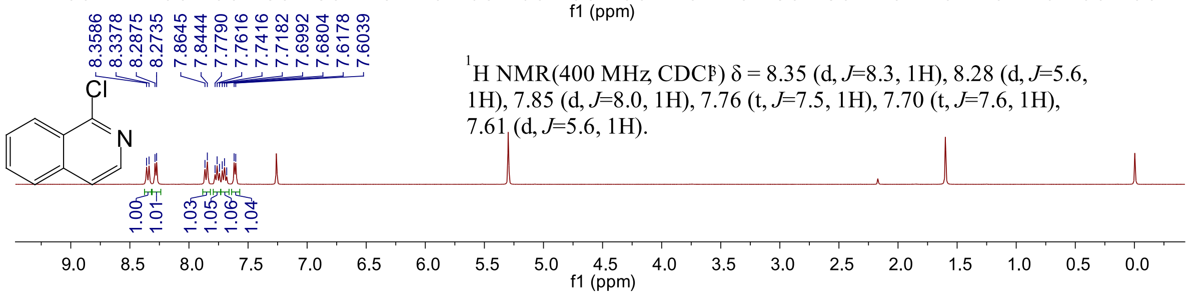

^{1}H NMR(400 MHz, $CDCl_3$) δ = 8.35 (d, *J*=8.3, 1H), 8.28 (d, *J*=5.6, 1H), 7.85 (d, *J*=8.0, 1H), 7.76 (t, *J*=7.5, 1H), 7.70 (t, *J*=7.6, 1H), 7.61 (d, *J*=5.6, 1H).

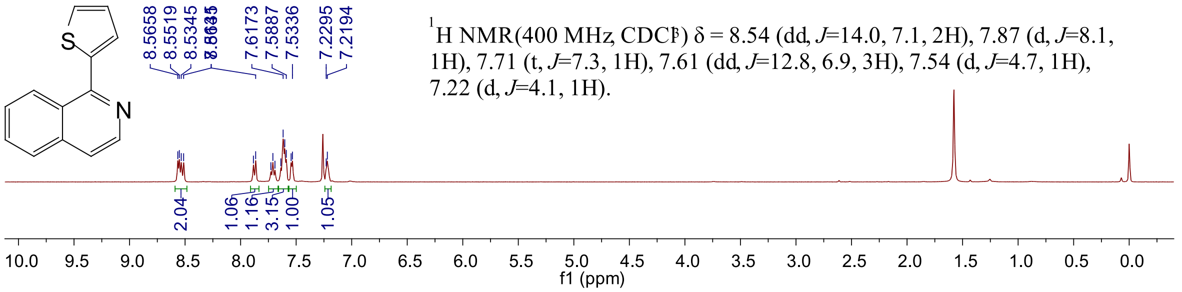

^{1}H NMR(400 MHz, $CDCl_3$) δ = 8.54 (dd, *J*=14.0, 7.1, 2H), 7.87 (d, *J*=8.1, 1H), 7.71 (t, *J*=7.3, 1H), 7.61 (dd, *J*=12.8, 6.9, 3H), 7.54 (d, *J*=4.7, 1H), 7.22 (d, *J*=4.1, 1H).

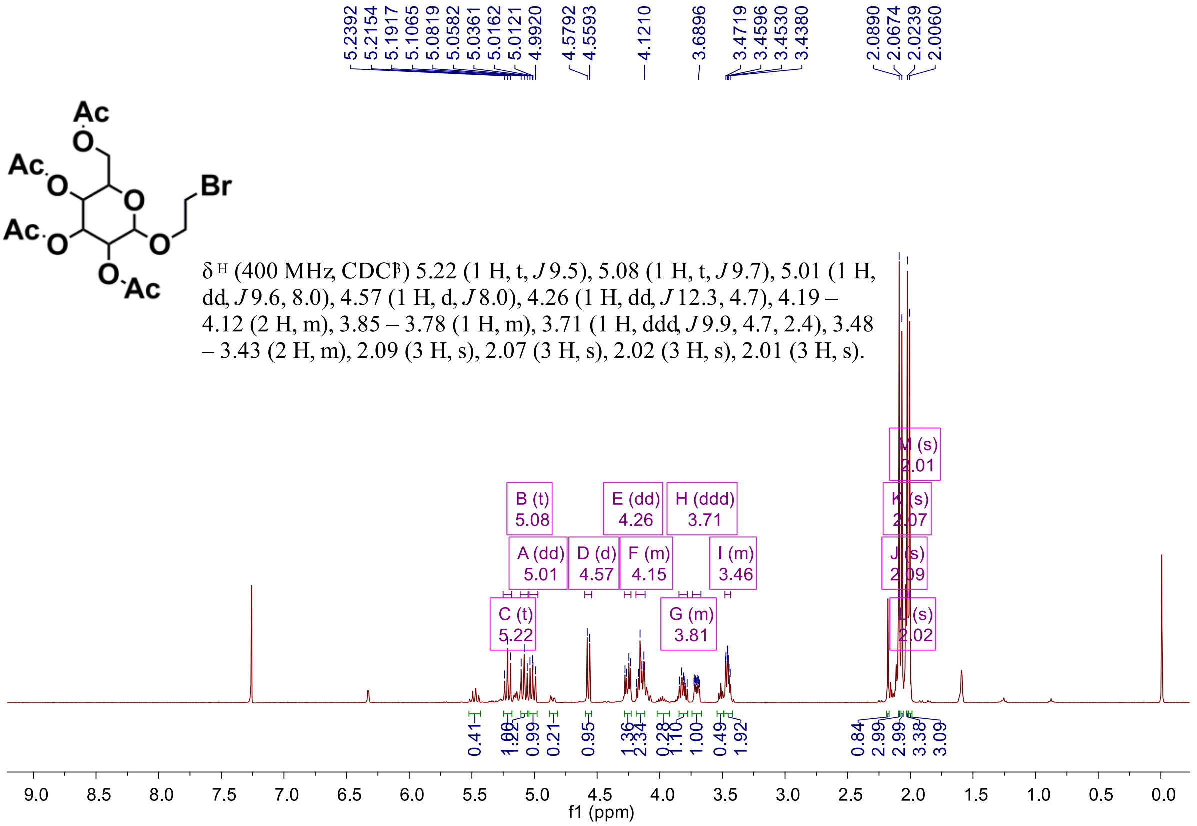

δ H (400 MHz, CDCl$_3$) 5.22 (1 H, t, *J* 9.5), 5.08 (1 H, t, *J* 9.7), 5.01 (1 H, dd, *J* 9.6, 8.0), 4.57 (1 H, d, *J* 8.0), 4.26 (1 H, dd, *J* 12.3, 4.7), 4.19 – 4.12 (2 H, m), 3.85 – 3.78 (1 H, m), 3.71 (1 H, ddd, *J* 9.9, 4.7, 2.4), 3.48 – 3.43 (2 H, m), 2.09 (3 H, s), 2.07 (3 H, s), 2.02 (3 H, s), 2.01 (3 H, s).

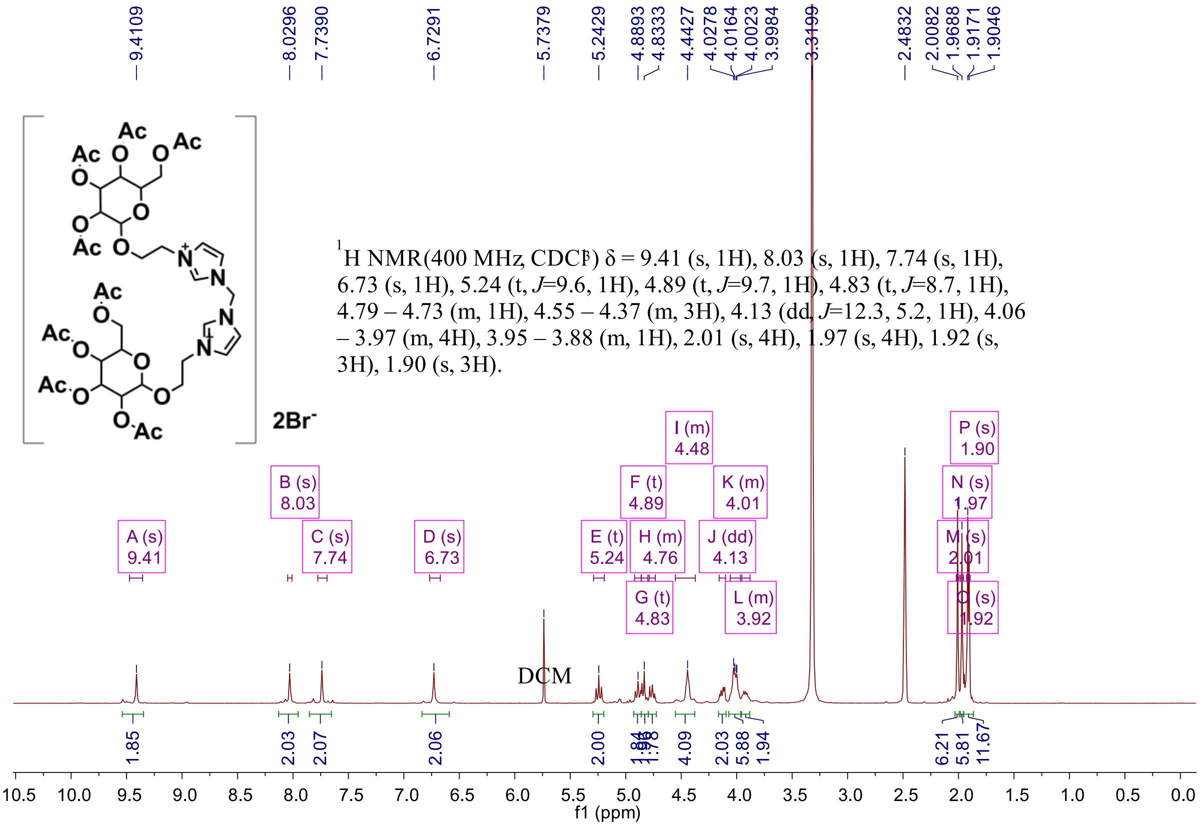

^{1}H NMR(400 MHz, CDCl$_3$) δ = 9.41 (s, 1H), 8.03 (s, 1H), 7.74 (s, 1H), 6.73 (s, 1H), 5.24 (t, *J*=9.6, 1H), 4.89 (t, *J*=9.7, 1H), 4.83 (t, *J*=8.7, 1H), 4.79 – 4.73 (m, 1H), 4.55 – 4.37 (m, 3H), 4.13 (dd, *J*=12.3, 5.2, 1H), 4.06 – 3.97 (m, 4H), 3.95 – 3.88 (m, 1H), 2.01 (s, 4H), 1.97 (s, 4H), 1.92 (s, 3H), 1.90 (s, 3H).

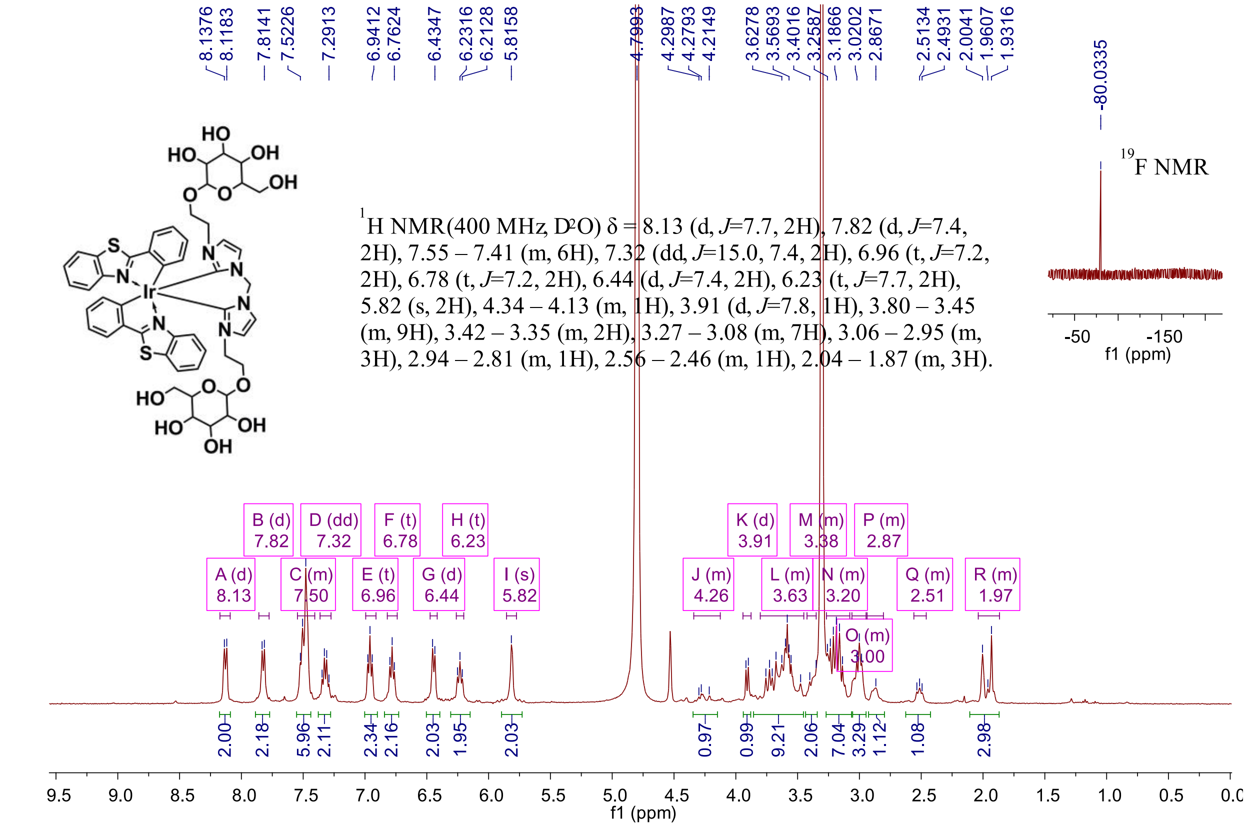

1H NMR(400 MHz, D_2O) δ = 8.13 (d, J=7.7, 2H), 7.82 (d, J=7.4, 2H), 7.55 – 7.41 (m, 6H), 7.32 (dd, J=15.0, 7.4, 2H), 6.96 (t, J=7.2, 2H), 6.78 (t, J=7.2, 2H), 6.44 (d, J=7.4, 2H), 6.23 (t, J=7.7, 2H), 5.82 (s, 2H), 4.34 – 4.13 (m, 1H), 3.91 (d, J=7.8, 1H), 3.80 – 3.45 (m, 9H), 3.42 – 3.35 (m, 2H), 3.27 – 3.08 (m, 7H), 3.06 – 2.95 (m, 3H), 2.94 – 2.81 (m, 1H), 2.56 – 2.46 (m, 1H), 2.04 – 1.87 (m, 3H).
19F NMR
-80.0335
f1 (ppm)

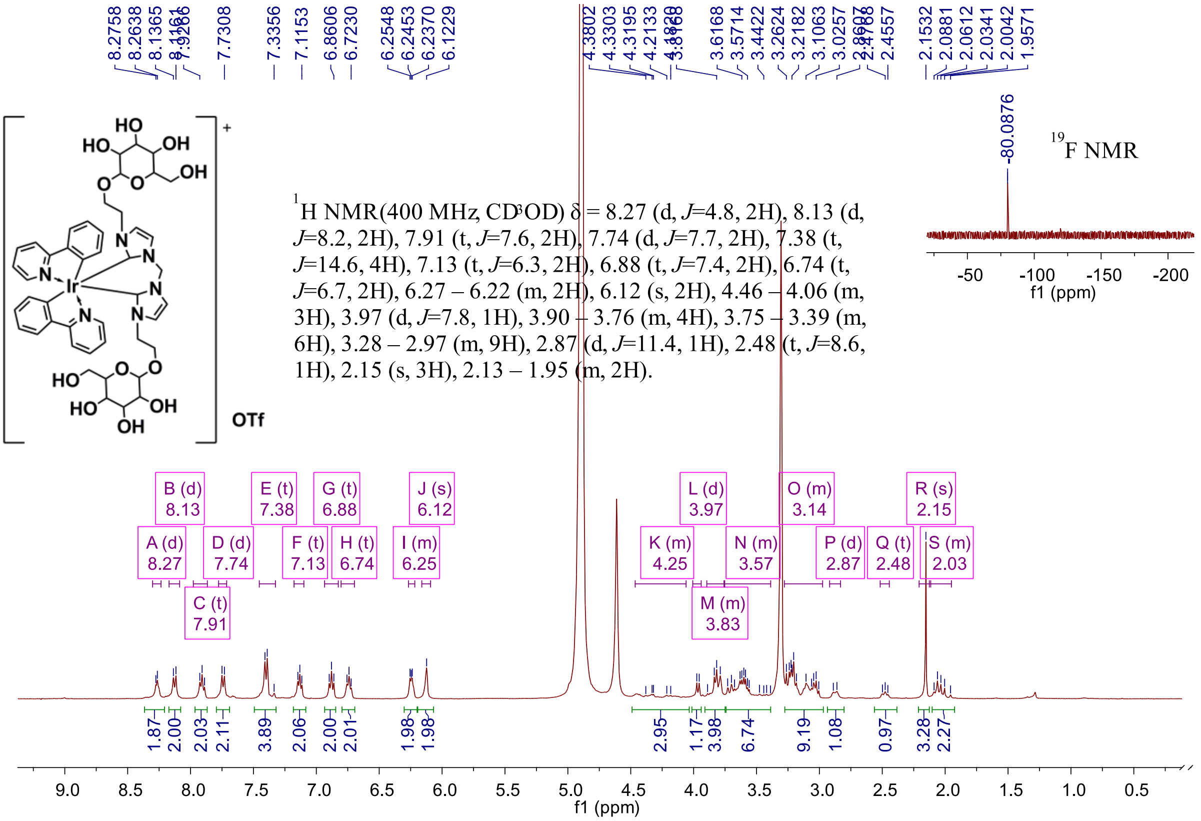

1H NMR(400 MHz, CD_3OD) δ = 8.27 (d, J=4.8, 2H), 8.13 (d, J=8.2, 2H), 7.91 (t, J=7.6, 2H), 7.74 (d, J=7.7, 2H), 7.38 (t, J=14.6, 4H), 7.13 (t, J=6.3, 2H), 6.88 (t, J=7.4, 2H), 6.74 (t, J=6.7, 2H), 6.27 – 6.22 (m, 2H), 6.12 (s, 2H), 4.46 – 4.06 (m, 3H), 3.97 (d, J=7.8, 1H), 3.90 – 3.76 (m, 4H), 3.75 – 3.39 (m, 6H), 3.28 – 2.97 (m, 9H), 2.87 (d, J=11.4, 1H), 2.48 (t, J=8.6, 1H), 2.15 (s, 3H), 2.13 – 1.95 (m, 2H).
19F NMR
-80.0876
OTf
f1 (ppm)

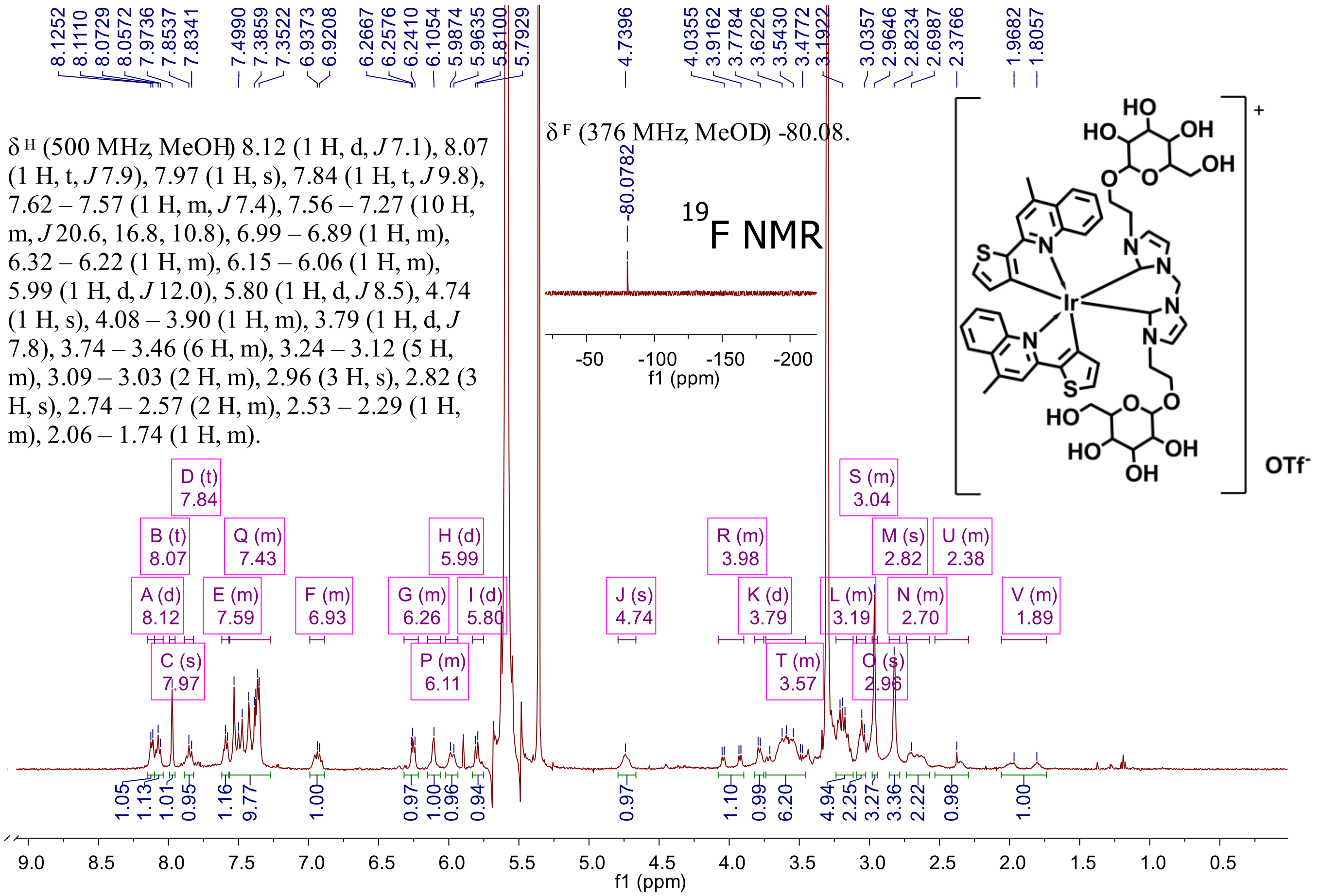
δH (500 MHz, MeOH) 8.12 (1 H, d, J 7.1), 8.07 (1 H, t, J 7.9), 7.97 (1 H, s), 7.84 (1 H, t, J 9.8), 7.62 – 7.57 (1 H, m, J 7.4), 7.56 – 7.27 (10 H, m, J 20.6, 16.8, 10.8), 6.99 – 6.89 (1 H, m), 6.32 – 6.22 (1 H, m), 6.15 – 6.06 (1 H, m), 5.99 (1 H, d, J 12.0), 5.80 (1 H, d, J 8.5), 4.74 (1 H, s), 4.08 – 3.90 (1 H, m), 3.79 (1 H, d, J 7.8), 3.74 – 3.46 (6 H, m), 3.24 – 3.12 (5 H, m), 3.09 – 3.03 (2 H, m), 2.96 (3 H, s), 2.82 (3 H, s), 2.74 – 2.57 (2 H, m), 2.53 – 2.29 (1 H, m), 2.06 – 1.74 (1 H, m).
δF (376 MHz, MeOD) -80.08.
19F NMR
OTf-
f1 (ppm)

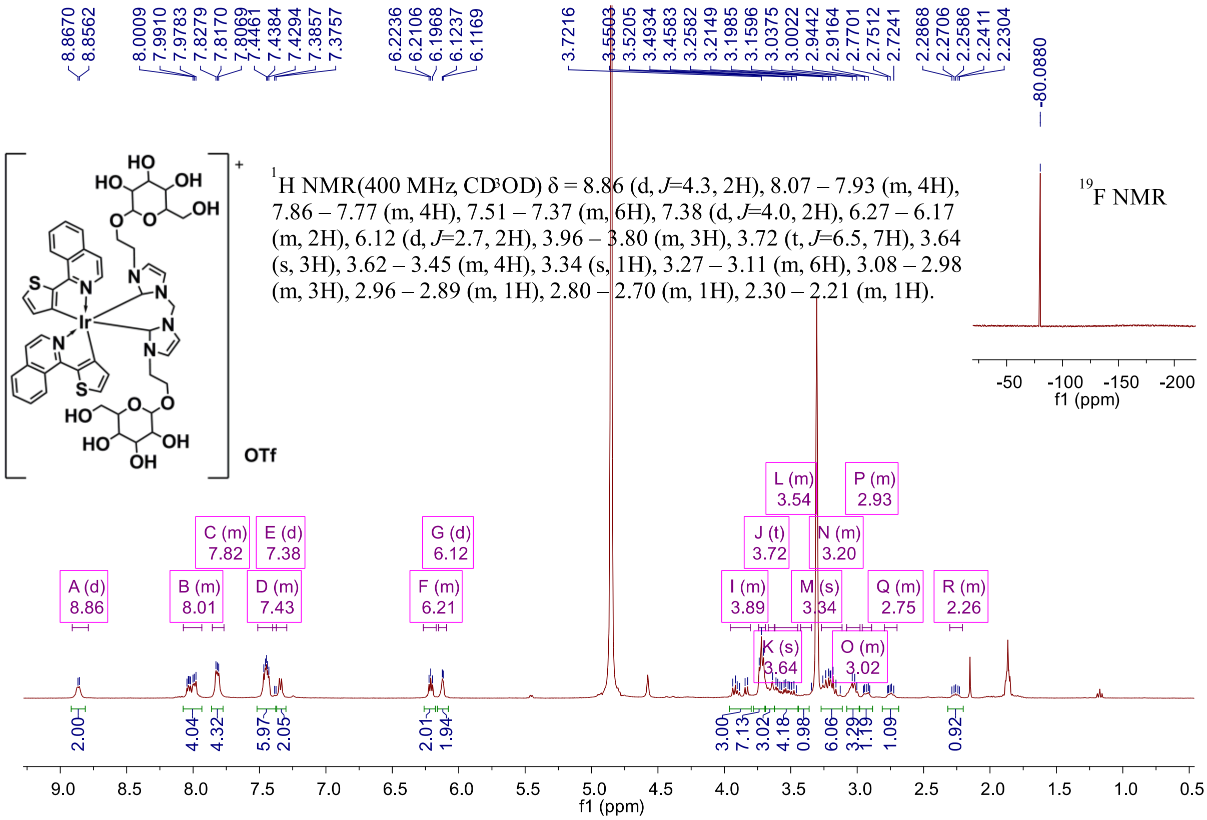
1H NMR(400 MHz, CD3OD) δ = 8.86 (d, J=4.3, 2H), 8.07 – 7.93 (m, 4H), 7.86 – 7.77 (m, 4H), 7.51 – 7.37 (m, 6H), 7.38 (d, J=4.0, 2H), 6.27 – 6.17 (m, 2H), 6.12 (d, J=2.7, 2H), 3.96 – 3.80 (m, 3H), 3.72 (t, J=6.5, 7H), 3.64 (s, 3H), 3.62 – 3.45 (m, 4H), 3.34 (s, 1H), 3.27 – 3.11 (m, 6H), 3.08 – 2.98 (m, 3H), 2.96 – 2.89 (m, 1H), 2.80 – 2.70 (m, 1H), 2.30 – 2.21 (m, 1H).
19F NMR
OTf
f1 (ppm)
